# Links between nucleolar activity, rDNA stability, aneuploidy and chronological aging in the yeast *Saccharomyces cerevisiae*

**DOI:** 10.1007/s10522-014-9499-y

**Published:** 2014-04-08

**Authors:** Anna Lewinska, Beata Miedziak, Klaudia Kulak, Mateusz Molon, Maciej Wnuk

**Affiliations:** 1 Department of Biochemistry and Cell Biology, University of Rzeszow, Rzeszow, Poland; 2 Centre of Applied Biotechnology and Basic Sciences, University of Rzeszow, Kolbuszowa, Poland; 3 Department of Genetics, University of Rzeszow, Rejtana 16C, 35-959 Rzeszow, Poland

**Keywords:** Yeast, Chronological aging, rDNA stability, Nucleolus, Aneuploidy, Cell cycle checkpoint control

## Abstract

The nucleolus is speculated to be a regulator of cellular senescence in numerous biological systems (Guarente, Genes Dev 11(19):2449–2455, 1997; Johnson et al., Curr Opin Cell Biol 10(3):332–338, 1998). In the budding yeast *Saccharomyces cerevisiae*, alterations in nucleolar architecture, the redistribution of nucleolar protein and the accumulation of extrachromosomal ribosomal DNA circles (ERCs) during replicative aging have been reported. However, little is known regarding rDNA stability and changes in nucleolar activity during chronological aging (CA), which is another yeast aging model used. In the present study, the impact of aberrant cell cycle checkpoint control (knock-out of *BUB1*, *BUB2*, *MAD1* and *TEL1* genes in haploid and diploid hemizygous states) on CA-mediated changes in the nucleolus was studied. Nucleolus fragmentation, changes in the nucleolus size and the nucleolus/nucleus ratio, ERC accumulation, expression pattern changes and the relocation of protein involved in transcriptional silencing during CA were revealed. All strains examined were affected by oxidative stress, aneuploidy (numerical rather than structural aberrations) and DNA damage. However, the *bub1* cells were the most prone to aneuploidy events, which may contribute to observed decrease in chronological lifespan. We postulate that chronological aging may be affected by redox imbalance-mediated chromosome XII instability leading to both rDNA instability and whole chromosome aneuploidy. CA-mediated nucleolus fragmentation may be a consequence of nucleolus enlargement and/or Nop2p upregulation. Moreover, the rDNA content of chronologically aging cells may be a factor determining the subsequent replicative lifespan. Taken together, we demonstrated that the nucleolus state is also affected during CA in yeast.

**Electronic supplementary material:**

The online version of this article (doi:10.1007/s10522-014-9499-y) contains supplementary material, which is available to authorized users.

## Introduction

The budding yeast *Saccharomyces cerevisiae* is considered an important model system with which to study the molecular mechanisms of physiological and pathophysiological processes of higher eukaryotes, especially aging (Kaeberlein 2010; Gershon and Gershon 2000). Two ways to measure aging have been established in yeast: replicative lifespan (RLS) and chronological lifespan (CLS) (Mortimer and Johnston 1959; Longo et al. 1996, 2012). RLS, reflects cellular replicative potential and indicates the number of daughter cells produced by a mother cell before senescence and replicative aging (RA) is a model of aging of mitotically active cells of higher eukaryotes (Kaeberlein 2010; Longo et al. 2012). In contrast, CLS is the survival time of cells in a non-dividing state in a stationary culture after transferring to fresh medium and chronological aging (CA) is a model of aging for post-mitotic cells of higher eukaryotes (Fabrizio and Longo 2003; MacLean et al. 2001; Chen et al. 2005). It is widely accepted that molecular damage accumulates (e.g. mitochondrial damage, oxidatively damaged/aggregated proteins) (Kaeberlein 2010) during both types of aging. Additionally, certain shared determinants of RLS and CLS have been reported (Kaeberlein 2010; Longo et al. 2012). Dietary restriction (DR) and reduced TOR signalling may contribute to the extension of both RLS and CLS (Jiang et al. 2000; Lin et al. 2000; Kaeberlein et al. 2004, 2005; Smith et al. 2007; Fabrizio et al. 2001; Powers et al. 2006). Several genetic and environmental factors that modulate replicative and chronological aging in yeast can also extend lifespan in other model organisms, which indicates that yeast aging shares conservation with aging in evolutionally divergent species (Longo et al. 2012).

Conversely, certain private mechanisms regulating RLS and CLS have been proposed (Sinclair and Guarente 1997; Burtner et al. 2009). The accumulation of extrachromosomal rDNA circles (ERCs), a sign of rDNA instability, is suggested to be a cause of replicative aging in yeast (Sinclair and Guarente 1997), whilst acetic acid accumulation in the medium is considered to be the primary cause of chronological aging in yeast (Burtner et al. 2009). The overexpression of Sir2p, histone deacetylase, and the knock-out of *FOB1* gene, which encodes replication fork blocking protein, resulted in rDNA stability and in turn contributed to RLS extension (Defossez et al. 1999; Kaeberlein et al. 1999). However, the yeast aging mechanism is much more complicated than previously thought. The results that are not consistent with the ERC theory have been reported (Heo et al. 1999; Kim et al. 1999; Hoopes et al. 2002; Merker and Klein 2002). Mutations in genes involved in DNA replication, DNA repair and transcription elongation result in rDNA instability and lifespan shortening (e.g. the *hpr1* or *dna2* cells lacking a component of the RNA polymerase II complex, Hpr1p and a replicative yeast helicase/nuclease, Dna2p, respectively) were reported to not result in ERC accumulation (Hoopes et al. 2002; Merker and Klein 2002). Thus, these findings may suggest that reduced lifespan is more associated with increased rDNA instability than ERC accumulation (Kobayashi 2008). Moreover, there are doubts about the primary interpretations of data on medium acidification during chronological aging (Longo et al. 2012). It seems that ethanol and acetic acid, at physiological concentrations, may act as carbon sources, which may block dietary restriction conditions. Medium acidification may accelerate chronological aging by nutrient sensing pathway activation and oxidative stress stimulation (Longo et al. 2012).

Because yeast chronological aging is a multifactorial phenomenon and its detailed mechanisms remain unknown, we decided to evaluate the role of rDNA stability and nucleolus state during CA in yeast. A comprehensive analysis of the nucleolus state during CA is lacking. There is one paper on aging phenotype characteristics, attributed solely to replicatively aging yeast cells (e.g., changes in nucleolar architecture, redistribution of transcriptional silencing complex, ERC accumulation), of stationary phase cells (Ashrafi et al. 1999). A chronological age-dependent decrease in RLS was shown, which was mediated by an unknown mechanism (Ashrafi et al. 1999). The majority (80–85 %) of the nucleoli in cells maintained for 21 days in rich YPD medium, containing 2 % of glucose, were unaffected and ERC accumulation was not observed (Ashrafi et al. 1999). However, within a subpopulation of chronologically aging cells (15–20 % of total cells examined), the redistribution of Sir3p and abnormal nucleoli were revealed (Ashrafi et al. 1999). Cells with an aging-like phenotype were unable to produce buds and re-enter the cell cycle after their transfer to fresh, rich medium (Ashrafi et al. 1999).

The most commonly used protocol with which to study CA in yeast was introduced by Longo (Fabrizio and Longo 2003; Longo et al. 2012). Cell survival is monitored after cell culture in flasks (growth and postdiauxic phases) in synthetic dextrose complete (SDC) medium containing 2 % of glucose and a four-fold excess of the supplements, followed by transfer to fresh solid YPD medium to retain cell growth. Cell growth is typically expressed in terms of colony forming units (CFUs) (Fabrizio and Longo 2003; Longo et al. 2012). During the chronological aging experiment, cells were kept in the glucose-depleted medium with ethanol as a primary carbon source (Longo et al. 2012). In the present study, CA-mediated changes in the nucleolus were monitored using the methodology described above. Surprisingly, we found that the yeast nucleolus was affected by chronological aging. Nucleoli were fragmented and the relocation of proteins involved in transcriptional silencing from telomeres to the nucleolus was revealed in both the haploid and diploid state. The expression pattern of nucleolar proteins was also changed: Nop2p and Sir3p were upregulated, whilst Rap1p was downregulated during CA. Moreover, we used genomic instability-promoting conditions, namely the cells devoid of four genes involved in cell cycle checkpoint control, *BUB1*, *BUB2*, *MAD1* and *TEL1*, and used chromosome specific probes from a panel of whole chromosome paining probes (WCPPs) to study a link between aneuploidy, nucleolar activity, rDNA stability and chronological aging in more detail. In general, haploid mutants were more affected by rDNA and multimer ERC accumulation during CA than the diploid mutants. Extensive aneuploidy in the *bub1* mutant may contribute to a decrease in CLS. Taken together, these findings indicate that changes in the nucleolus that affect genome integrity may modulate CLS in yeast.

## Materials and methods

### Chemicals

Dihydroethidium and rhodamine G6 were obtained from Molecular Probes (Leiden, Netherlands) and phosphate-buffered saline (PBS) (1.54 mM potassium phosphate monobasic, KH_2_PO_4_, 155.17 mM sodium chloride, NaCl, 2.71 mM sodium phosphate dibasic, Na_2_HPO_4_ × 7H_2_O, pH 7.2) was obtained from Gibco, Invitrogen Corporation (Grand Island, NY, USA). All other reagents, if not stated otherwise, were purchased from Sigma (Poznan, Poland) and were of analytical grade.

### Strains and growth conditions

All yeast strains used in this study are listed in Table 1. Yeast from one single colony was grown overnight on liquid YPD medium (1 % w/v Difco Yeast Extract, 2 % w/v Difco Yeast Bacto-Peptone, 2 % w/v dextrose). The yeast were then washed and transferred to liquid synthetic dextrose complete (SDC) medium (0.67 % Difco Yeast Nitrogen Base with ammonium sulphate and without amino acids, 2 % dextrose and with a four-fold excess of essential amino acids and nucleotide supplementation) and cultured with shaking in a water bath incubator at 28 °C for up to 4 weeks. Cells were cultured in flasks with a volume-to-medium ratio of 5:1. The maximum population density is reached after the first 2 days of growth in SDC medium (approximately 1 × 10^8^ cells/ml) and thus the number of colony forming units (CFUs) on day 2 (assuming that the dilution day is day 0) is considered the initial survival (100 % survival). Typically, at the indicated time points, appropriate aliquots were taken for analysis. For aneuploidy analysis, chronologically aging cells were transferred to fresh YPD medium to continue mitotic growth, after which the cells were subjected to fluorescence in situ hybridisation (FISH).

**Table 1:** Strains used in this study

Strain	Genotype	Source
BY4741	*MATa his3 leu2 met15 ura3*	EUROSCARF
BY4743	*MATa/MATα his3/his3 leu2/leu2 met15/MET15 lys2/LYS2 ura3/ura3*	EUROSCARF
*bub1*	BY4741 *YGR188c::kanMX4*	EUROSCARF
*bub2*	BY4741 *YMR055c::kanMX4*	EUROSCARF
*mad1*	BY4741 *YGL086w::kanMX4*	EUROSCARF
*tel1*	BY4741 *YBL088c::kanMX4*	EUROSCARF
*BUB1/bub1*	BY4743 *YGR188c::kanMX4/YGR188c*	EUROSCARF
*BUB2/bub2*	BY4743 *YMR055c::kanMX4/YMR055c*	EUROSCARF
*MAD1/mad1*	BY4743 *YGL086w::kanMX4/YGL086w*	EUROSCARF
*TEL1/tel1*	BY4743 *YBL088c::kanMX4/YBL088c*	EUROSCARF

Haploid and diploid hemizygous mutants were confirmed on YPD Petri dishes containing 200 μg/ml geneticin (G418) sulphate and SDC plates +His +Leu +Ura −Met −Lys, respectively.

### Chronological life span (CLS) assays

For the quantitative measurement of survival, the CFU assay and the kinetics of growth assay were used. For the semiquantitative measurement of survival, the spot assay was used.

For the CFU assay, at the indicated time points (day 2, 7, 14, 21 and 28), cells were removed, washed, diluted and spread onto YPD agar plates. After 48 h, the CFUs/ml were counted (Lewinska et al. 2011).

For the kinetics of growth assay (Lewinska et al. 2011), at the indicated time points (day 2, 7, 14, 21 and 28), cells were removed, washed, diluted, suspended in YPD medium (a total volume of 150 μl with working concentration of 5 × 10^6^ cells/ml) and cultured in a 96-well format shaker at 900 rpm at 28 °C. Their growth was monitored turbidimetrically at 600 nm in a Thermo Scientific microplate reader every 2 h during a 12 h period.

For the spot assay (Lewinska et al. 2011), at the indicated time points (day 2, 7, 14, 21 and 28), cells were removed, washed, diluted (10^7^, 10^6^, 10^5^, 10^4^, 10^3^ cells/ml) in a volume of 2 μl, inoculated on solid YPD medium and inspected after 48 h.

Cell viability was estimated with a LIVE/DEAD^®^ Yeast Viability Kit (Molecular Probes, Netherlands) using the standard protocol according to the manufacturer’s instructions. Briefly, at the indicated time points (day 2, 7, 14, 21 and 28), cells were washed and stained with a mixture of FUN^®^ 1 and Calcofluor^®^ White M2R and inspected under an Olympus BX61 fluorescence microscope equipped with a DP72 CCD camera and Olympus CellF software. Typically, a total of 300 cells were used for the analysis.

### Chronological lifespan to replicative lifespan assay

The reproductive potential of chronologically aging cells was estimated with routine RLS procedure using a micromanipulator. Briefly, 5 µl of the chronological aging culture was dropped onto YPD plates containing 10 μg/ml Phloxine B (Minois et al. 2005). For each experiment, Phloxine B-negative cells were selected and 40 viable cells were micromanipulated. During the RLS experiment, the plates were kept at 28 °C for 16 h, later at 4 °C overnight.

### Oxidative stress parameters

At the indicated time points (day 2, 7, 14, 21 and 28), the steady-state level of reactive oxygen species (ROS) in the cell culture medium and intracellular superoxide production were measured with 2′,7′-dichlorodihydrofluorescein diacetate (H_2_DCF-DA) and dihydroethidium, respectively. Briefly, 5 µM H_2_DCF-DA was added to the medium (supernatant obtained after cell centrifugation) and the fluorescence of the 2′,7′-dichlorofluorescein (DCF) formed was monitored in a Tecan Infinite^®^ M200 fluorescence mode microplate reader. The measurement conditions were: λ_ex_ = 495 nm and λ_em_ = 525 nm. The steady-state level of ROS is presented as RFU (relative fluorescence unit). For superoxide kinetics (Lewinska et al. 2011), cells (10^8^ cells/ml) were washed and suspended in PBS containing 0.1 % glucose, 0.5 mM EDTA and 20 μM dihydroethidium. Fluorescence intensity due to the oxidation of dihydroethidium to ethidium was monitored in a Tecan Infinite^®^ M200 fluorescence mode microplate reader. Measurement conditions were: λ_ex_ = 518 nm and λ_em_ = 605 nm; temperature 28 °C. Data are presented as RFU (relative fluorescence unit) per minute.

CA-mediated protein carbonylation was estimated with an OxyBlot™ Protein Oxidation Detection Kit (Millipore Corporation, USA) using the standard protocol according to the manufacturer’s instructions. Briefly, protein cell extracts were obtained via glass bead lysis, and the protein concentration was estimated with Bradford reagent using bovine serum albumin (BSA) as the standard (Sigma, Poland). Then, proteins (20 µg) were derivatised with 2,4-dinitrophenylhydrazine (DNPH) and were resolved by 10 % SDS–PAGE and electroblotted onto an Immobilon polivinylidene difluoride (PVDF) membrane (Millipore Corporation, USA). The membrane was blocked with 1 % BSA in TBST (20 mM Tris–HCl, pH 7.5, 137 mM NaCl containing 0.1 % Tween 20) at room temperature for 1 h and protein carbonylation (proteins with DNP residues) was detected using rabbit anti-DNP antibody (diluted 1:150 in 1 % BSA in TBST, overnight incubation at 4 °C) (Millipore Corporation, USA) and goat anti-rabbit HRP-conjugated antibody (diluted 1:300 in 1 % BSA in TBST, 1 h incubation at room temperature) (Millipore Corporation, USA). For each oxyblot analysis, a negative control (no DNPH derivatisation) and a positive control with a mixture of standard proteins with attached DNP residues were applied. The chemiluminescence signal was detected with ECL Plus Western Blotting Detection System (GE Healthcare, Freiburg, Germany) and a G:BOX imaging system (Syngene, Cambridge, UK).

### Mitochondrial membrane potential (Δ_ψm_)

Yeast cells (1 × 10^8^ cells/ml) were incubated with 5 µM rhodamine G6 in PBS containing 0.1 % glucose and 0.5 mM EDTA at 28 °C for 15 min. The cells were then washed and the fluorescence intensity reflecting the mitochondrial membrane potential was monitored in a Tecan Infinite^®^ M200 fluorescence mode microplate reader. The measurement conditions were: λ_ex_ = 528 nm and λ_em_ = 551 nm; temperature 28 °C. Mitochondrial membrane potential is presented as relative fluorescence units, RFUs. Additionally, after rhodamine G6 staining, cells were inspected under an Olympus BX61 fluorescence microscope equipped with a DP72 CCD camera and Olympus CellF software.

### Nucleolus morphology and size

To visualise the nucleolus, silver staining of nucleolar organiser regions (AgNOR) was performed. Silver staining of nucleolar argyrophilic proteins was conducted according to Howell and Black (1980) by incubating the cell slides with a colloidal developer containing 50 % AgNO_3_ in the dark at 37 °C for 15 min. After washing in tap water, the preparations were stained with 5 % Giemsa for 10 s and nucleoli were captured with an Olympus BX61 light microscope equipped with a DP72 CCD camera and Olympus CellF software. A total of 100 cells were analysed and their nucleolus morphological type was determined (normal, increased or fragmented nucleolus) [%]. Nucleolus size and nucleolus/nucleus ratio were calculated using ImageJ software http://rsbweb.nih.gov/ij/.

### Nop2p, Sir3p and Rap1p immunostaining

Chronologically aging yeast were diluted to 10^7^ cells/ml in PBS and fixed with 37 % formaldehyde (9:1, v/v, 3.7 % final formaldehyde concentration). After a 1-h incubation, cells were washed with PBS and suspended in a spheroplast buffer (1.2 M sorbitol in sterile PBS). β-mercaptoethanol (1.42 M), 16 µl and zymolyase 100T (5 mg/ml), 25 µl were added to 1 ml of the cell suspension and incubated with shaking at 28 °C for 1 h. After sedimentation, the cells were washed, suspended in 500 µl of the spheroplast buffer, spread onto poly-l-lysine-coated slides and permeabilised with PBS containing 0.1 % Triton X-100. Spheroplast-coated slides were blocked with 1 % BSA in TBST (20 mM Tris–HCl, pH 7.5, 137 mM NaCl containing 0.1 % Tween 20) at room temperature for 30 min and:

1. Nop2p was detected using a mouse monoclonal antibody against Nop2p (diluted 1:1,000 in 1 % BSA in TBST, overnight incubation at 4 °C) (Santa Cruz Biotechnology, Germany) and anti-mouse Texas Red-conjugated antibody (diluted 1:500 in 1 % BSA in TBST, 1 h incubation at room temperature) (Santa Cruz Biotechnology, Germany);
2. Rap1p was detected using a goat polyclonal antibody against Rap1p (diluted 1:200 in 1 % BSA in TBST, overnight incubation at 4 °C) (Santa Cruz Biotechnology, Germany) and anti-goat FITC-conjugated antibody (diluted 1:500 in 1 % BSA in TBST, 1 h incubation at room temperature) (Santa Cruz Biotechnology, Germany);
3. Sir3p was detected using a rabbit polyclonal antibody against Sir3p (diluted 1:200 in 1 % BSA in TBST, overnight incubation at 4 °C) (Santa Cruz Biotechnology, Germany) and anti-rabbit FITC-conjugated antibody (diluted 1:500 in 1 % BSA in TBST, 1 h incubation at room temperature) (Santa Cruz Biotechnology, Germany).

For DNA visualisation, the slides were counterstained with a drop of anti-fade mounting medium with 4′,6′-diamino-2-phenylindole (DAPI) (Cambio, UK) and were analysed using an Olympus BX61 fluorescence microscope equipped with a DP72 CCD camera and Olympus CellF software.

### Western blotting

Whole cell extracts were obtained via glass bead lysis in the presence of 20 mM phosphate buffer, pH 7.0, containing 1 mM EDTA and yeast protease inhibitor cocktail (Sigma, Poland). The protein concentration was estimated with Bradford reagent using BSA as the standard (Sigma, Poland). Proteins (50 μg) were resolved by 10 % SDS-PAGE and electroblotted onto an Immobilon polivinylidene difluoride (PVDF) membrane (Millipore Corporation, USA). The membrane was blocked with 1 % BSA in TBST at room temperature for 1 h and:

1. Nop2p was detected using a mouse monoclonal antibody against Nop2p (diluted 1:2,000 in 1 % BSA in TBST, overnight incubation at 4 °C) (Santa Cruz Biotechnology, Germany) and anti-mouse IgG-peroxidase antibody (diluted 1:80,000 in 1 % BSA in TBST, 1 h incubation at room temperature) (Sigma, Poland);
2. Rap1p was detected using a goat polyclonal antibody against Rap1p (diluted 1:200 in 1 % BSA in TBST, overnight incubation at 4 °C) (Santa Cruz Biotechnology, Germany) and anti-goat IgG-peroxidase antibody (diluted 1:80,000 in 1 % BSA in TBST, 1 h incubation at room temperature) (Sigma, Poland);
3. Sir3p was detected using a rabbit polyclonal antibody against Sir3p (diluted 1:200 in 1 % BSA in TBST, overnight incubation at 4 °C) (Santa Cruz Biotechnology, Germany) and anti-rabbit IgG-peroxidase antibody (diluted 1:80,000 in 1 % BSA in TBST, 1 h incubation at room temperature) (Sigma, Poland).

For the loading control, rabbit polyclonal antibody against actin (diluted 1:1,000 in 1 % BSA in TBST, overnight incubation at 4 °C) (Abcam, UK) and anti-rabbit IgG-peroxidase antibody (diluted 1:80,000 in 1 % BSA in TBST, 1 h incubation at room temperature) (Sigma, Poland) were used. The chemiluminescence signal was detected with an ECL Plus Western Blotting Detection System (GE Healthcare, Freiburg, Germany) and a G:BOX imaging system (Syngene, Cambridge, UK).

### Extrachromosomal rDNA circles (Southern blot analysis)

To create an rDNA specific probe, the pNOY373 plasmid, a derivative of the high copy number plasmid YEp351 carrying rDNA with a promoter starting from –206 with a *XhoI*–*NotI* flanked enhancer, *LEU2*, 2µ, *amp*, was used. The pNOY373 plasmid was kindly provided by Prof. Masayasu Nomura (University of California, USA) (Wai et al. 2000). pNOY373 DNA containing the 18S rRNA coding region [1 µg] was labelled with digoxigenin-11-deoxyuridine 5′-triphosphate (dUTP) using the DIG Nick Translation Mix (Roche) according to manufacturer’s instruction. After PFGE separation (CHEF-DR^®^III Pulsed Field Electrophoresis System, Biorad), yeast chromosomes were transferred onto a nylon membrane (Roche) by capillary transfer. Then, the membrane was hybridised to a digoxigenin (DIG)-labelled rDNA-specific probe and rDNA was detected with an alkaline phosphatase-conjugated anti-DIG antibody. The chemiluminescence signal was detected with the substrate for alkaline phosphatase (CDP-Star) and the G:BOX imaging system (Syngene, Cambridge, UK). rDNA and ERCs were quantified using GelQuantNET software (http://biochemlabsolutions.com/GelQuantNET.html) using the background correction option. The amount of genomic rDNA and multimer ERCs were calculated per mean amount of DNA.

### RNA degradation

RNA was isolated using an RNeasy Mini Kit (Qiagen, USA). To evaluate RNA degradation during CA, RNA chip electrophoresis was performed using an Experion™ Automated Electrophoresis System and an Experion™ RNA StdSens Analysis Kit (Biorad, Germany). Briefly, RNA electrophoresis was conducted in the channels of microchips and the fluorescence of a fluorophore-bound RNA was measured. RQI (an RNA quality indicator) algorithm was used to assess RNA integrity by comparing the electropherogram of RNA samples to a series of standardised degraded RNA samples. RNA electropherograms were transformed to virtual gel images.

### Aneuploidy and nucleolar rDNA quantitation

CA-mediated structural and numerical aberrations were analysed. For gross structural aberrations, PFGE separation of yeast DNA was performed according to the manufacturer’s instructions using a CHEF-DR^®^III Pulsed Field Electrophoresis System (Biorad). For numerical aberrations, fluorescence in situ hybridisation (FISH) with whole chromosome painting probes (WCPPs) was applied. Chronologically aging yeast were transfer to fresh YPD medium to continue mitotic growth. Cell fixation and spheroplast preparation methods are given in the subsection on nucleolar protein immunostaining. The slides were treated with 100 µg/ml RNAse in 2× saline sodium citrate (SSC) buffer (1× SSC buffer: 150 mM sodium chloride in 15 mM sodium citrate, pH 7.0) in a humidified chamber at 37 °C for 1 h for better results. Next, the slides were washed three times in 2× SSC buffer and were treated with 1 % pepsin in 10 mM HCl in a coplin jar at 37 °C for 10 min. Then, the slides were washed twice in PBS, once in PBS supplemented with 50 mM MgCl_2_ and in a graded series of ethanol solutions (70, 80 and 95 %) at room temperature for 3 min each. A biotinylated probe specific to chromosome I, chromosome V or chromosome XII (Polish Patent Office, registration number P.404526) was added to the slide. The production of whole chromosome painting probes (WCPPs) that are fairly specific to each particular chromosome has been previously described in the application number P.404526 (Polish Patent Office) (Wnuk and Lewinska 2013). Briefly, after the separation of each particular yeast chromosome with PFGE and cutting out selected bands with chromosome of interest using a razor blade, chromosomal DNA was extracted using a DNA isolation kit (Biological Industries Israel Beit Haemek Ltd.), dried with a SpeedVac Concentrator (Savant) and suspended in 10 µl of ultra-pure water (UPW). Then, chromosomal DNA was labelled using a Biotin-High Prime DNA Labelling Kit with biotin-16-dUTP and using random oligonucleotides as primers (Roche) according to a standard procedure provided by manufacturer. To precipitate biotin-labelled DNA, 100 µg/ml yeast tRNA, 5 µl of 10 M ammonium acetate and 100 µl of ice-cold absolute ethanol were added. After centrifugation for 30 min (14,000 g, 4 °C), supernatant was discarded and pellet containing labelled DNA complementary to the yeast chromosome of interest was dried using a SpeedVac Concentrator. Whole chromosome painting probes (biotin-labelled chromosome-specific DNA) with yeast tRNA (100 µg/ml) were added to hybridisation buffer (Kreatech). To dissolve WCPPs, biotin-labelled chromosome-specific DNA was incubated at 4 °C for 48 h. WCPPs are stable at 4 °C for 6 months.

Co-denaturation of the sample and probe on a microscope slide was carried out on a hot plate at 80 °C for 7 min. Hybridisation was performed in a humidified chamber at 37 °C for 48 h. The slides were washed with 1× Wash Buffer I (0.4× SSC, pH 7.2, containing 0.3 % Igepal CA-630) at 70 °C for 2 min and were then washed with 1× Wash Buffer II (2× SSC containing 0.1 % Igepal CA-630) at room temperature for 1 min. To detect the biotinylated probe, a Star*FISH^©^ Biotin Painting Kit—FITC Label (Cambio, UK) was used. For DNA visualisation, the slides were counterstained with a drop of anti-fade mounting medium with 4′,6′-diamino-2-phenylindole (DAPI) (Cambio, UK) and were analysed using an Olympus BX61 fluorescence microscope equipped with a DP72 CCD camera and Olympus CellF software. Aneuploidy events were counted and presented as a percentage of 200 total cell scores. Because chromosome XII contains the rDNA locus in yeast, we also quantitated the nucleolar rDNA as fluorescent chromosome XII-specific signals using ImageJ software http://rsbweb.nih.gov/ij/. We evaluated the integrated fluorescence density (green channel), which is the sum of all pixel values within the marked area of each chromosome XII analysed and equivalent to the product of area and the mean gray value. The integrated fluorescence density is presented in relative fluorescence units (RFUs).

### Chromosome comet assay

After PFGE separation, yeast chromosomes were stained with ethidium bromide and selected bands were excised using a razor blade. DNA breaks within chromosome XII were visualised via an alkaline chromosome comet assay (pH > 13) (Lewinska et al. 2014). Briefly, a few layers of agarose were placed on poly-l-lysine-coated microscope slide, namely from the bottom to the top: 0.8 % SeaKem^®^ GTG™ Agarose (Lonza, Switzerland), 0.6 % SeaPlaque™ Low Melting (LM) Agarose (Lonza, Switzerland) and 0.5 % SeaKem^®^ GTG™ Agarose (Lonza, Switzerland). Within the LM agarose layer, holes were created and bands containing chromosome XII after PFGE separation were added. The electrophoretic conditions were as follows: 30 mM NaOH/1 mM EDTA, pH > 13, 30 min, 100 mA. After electrophoresis, the chromosomal DNA was neutralised, precipitated and stained with YOYO-1 (Molecular Probes, Netherlands) staining solution. Immediately after the staining step, DNA breaks and structural aberrations (termed in this report as abnormal chromosome morphology) were visualised using an Olympus BX61 fluorescence microscope equipped with a DP72 CCD camera and Olympus CellF software. The CCD capture conditions were the following: exposure time 350 ms, 100× oil immersion objective. YOYO-1 fluorescent signals were collected using a FITC filter (λ_ex_ = 491 nm and λ_em_ = 509 nm). A total of 100 chromosomes per each sample triplicate were analysed, and the percentage of DNA damage and structural aberrations was calculated.

### Statistical analysis

The results represent the mean ± SD from at least three independent experiments. All microscopic evaluations were performed on randomised and coded slides. The statistical significance of differences in parameters examined between: (i) wild types and corresponding mutants during CA and (ii) different time points of CA within the same strain were assessed with the one-way analysis of variance (ANOVA) with post hoc testing using a Dunnett’s multiple comparison test. A *p* value <0.05 was considered to be significant. The statistical analyses were performed using StatSoft, Inc. (2005), STATISTICA, version 7.0 (http://www.statsoft.com).

## Results

**Fig. 1:**
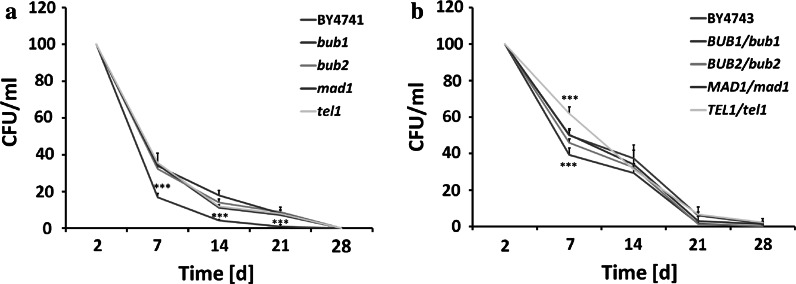
CLS of haploid wild type strain BY4741 and isogenic *bub1*, *bub2*, *mad1* and *tel1* mutants (**a**) and CLS of diploid wild type strain BY4743 and isogenic *BUB1*/*bub1*, *BUB2*/*bub2*, *MAD1*/*mad1* and *TEL1*/*tel1* mutants (**b**). After 2, 7, 14, 21 and 28 days, appropriate aliquots from CA cultures were taken for analysis. The cells were spread onto YPD plates and the ability to form colonies was checked after 48 h. *Bars* indicate SD, *n* = 3, ***p < 0.001 compared to the CFU of wild type strain (ANOVA and Dunnett’s a posteriori test)

Firstly, we addressed the question of whether the CLS of mutant cells lacking the *BUB1*, *BUB2*, *MAD1* and *TEL1* genes may be affected compared to their corresponding wild type cells. We used different methodologies: CFU (colony forming unit), growth kinetics and spot assays. The ability to form colonies on solid rich medium was evidently diminished in the haploid *bub1* background (*p* < 0.001), whilst CLS of other mutants was more or less comparable to wild type CLS (Fig. 1).

In the diploid *BUB1/bub1* state, a statistically significant decrease in CFU was only shown at day 7 of chronological aging (Fig. 1). On the contrary, on day 7, the CFU of the *TEL1/tel1* mutant was augmented compared to the CFU of the BY4743 wild type (*p* < 0.001) (Fig. 1).

The CLS of the *bub1* cells was also shortened compared to wild type as estimated with kinetic growth rate and spot assays (Figs. S1, S2).

Although CFU (Fig. 1) and spot assays (Fig. S2) did not reveal significant differences in CLS between cells devoid of Bub2p, Mad1p and Tel1p in haploid and hemizygous diploid states and wild types, a delay in the growth rate was observed when chronologically aging cells were transferred to fresh rich liquid medium and cultured up to 12 h (Fig. S1). The *bub1* and *mad1* cells were the most affected mutants (*p* < 0.001), whilst the growth rate of the *tel1* cells was augmented compared to the growth rate of wild type at day 21 and day 28 of chronological culture (*p* < 0.001) (Fig. S1). In the diploid state, delayed mutant growth was observed at day 7 of chronological aging culture, whilst at day 14, the growth kinetics of some mutants, namely the *BUB2/bub2* and *MAD1/mad1* mutants, was improved compared to the growth rate of the BY4743 wild type strain (*p* < 0.001) (Fig. S1). Such discrepancies may result from the duration of the experiment (48 h for the CFU and spot assay versus 12 h for the kinetic growth assay). An initial growth delay of mutant cells (Fig. S1), with the exception of the *bub1* cells, may be masked when cells are cultured for up to two days (Figs. 1, S2).

We were able to observe the regrowth/“gasping” phenomenon, especially when chronologically aging cells were taken for analysis after day 21 and day 28 of culture. Regrowth is considered an adaptive response that enables cell subpopulation to escape from quiescence and re-enter the cell cycle. The capability of cells to re-enter the cell cycle during chronological aging culture (day 21 and day 28, spot assay) was evidently observed for the *mad1* and *tel1* mutant cells as well as for the *MAD1/mad1* and *TEL1/tel1* cells, which resulted in growth improvement (an increase in survival) late in the CLS experiment (Fig. S3).

**Fig. 2:**
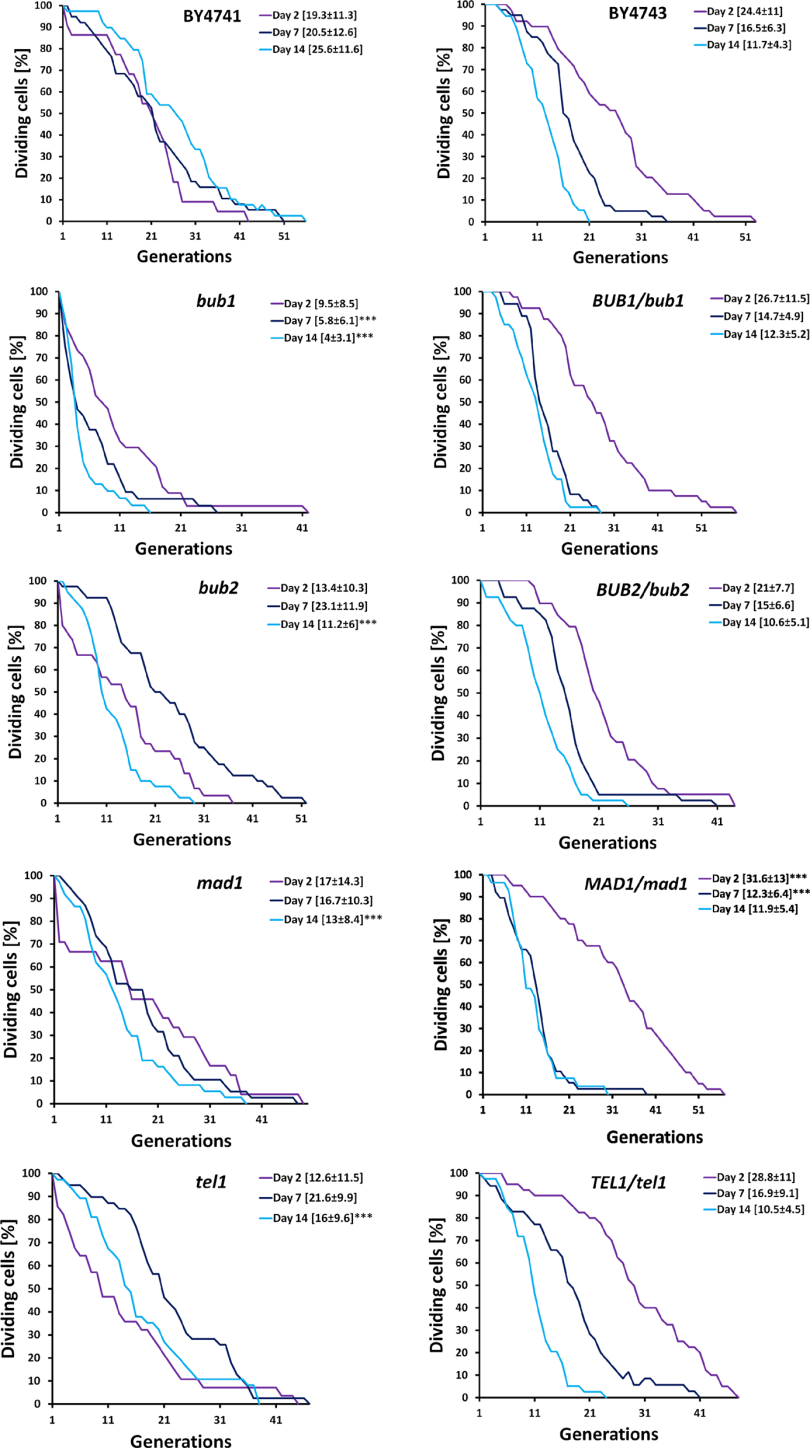
CLS to RLS assay. Replicative potential of haploid wild type strain BY4741 and isogenic *bub1*, *bub2*, *mad1* and *tel1* mutants (*left*), and diploid wild type strain BY4743 and isogenic *BUB1*/*bub1*, *BUB2*/*bub2*, *MAD1*/*mad1* and *TEL1*/*tel1* mutants (*right*) are presented. After 2, 7 and 14 days, appropriate aliquots from CA cultures were taken for replicative lifespan analysis. Mean replicative lifespan ± SD is shown in square brackets, ***p < 0.001 compared to the RLS of wild type strain (ANOVA and Dunnett’s a posteriori test)

We were also interested to see if CLS may affect RLS. Chronologically aging cells (day 2, day 7 and day 14) were subjected to reproductive potential analysis (RLS assay) (Fig. 2).

CLS-dependent RLS was evident for all diploid strains examined with mostly minor insignificant differences between the mutants and wild type (Fig. 2). In contrast, the relationship between CLS and subsequent RLS of haploid strains was much more complex. With the exception of the *bub1* cells, at day 7 of chronological culture, the RLS of other mutants was increased compared to day 2, while at day 14 RLS was moderately decreased (the *bub2* and *mad1* cells) or increased (the *tel1* cells) compared to day 2 (Fig. 2). The RLS of haploid wild type BY4741 seems to be largely CLS-independent, which may be due to more frequently observed regrowth phenomenon in the haploid state (Fig. 2). However, the RLS of the *bub1* haploid mutant was limited by CLS, and the reduction in RLS was pronounced compared to the wild type strain (*p* < 0.001) (Fig. 2). At day 14, the RLS of all haploid mutants examined was significantly decreased compared to the RLS of the wild type (*p* < 0.001) (Fig. 2).

During chronological aging, the number of dead cells was augmented (Fig. S4).

The level of dead cells accumulated more rapidly in the *bub1* background than in the wild type, e.g., at day 7 of culture—there were 38 and 64 % of dead cells in BY4741 and the *bub1* mutant, respectively (Fig. S4). Conversely, the level of dead cells in the *tel1* mutant was indistinguishable from that of the wild type strain during chronological aging culture (Fig. S4).

Because oxidative stress may limit CLS, we were interested in determining if the imbalance in intracellular redox equilibrium may be more pronounced in the mutant cells during chronological aging. We monitored the oxidation state of the chronological aging medium, intracellular superoxide production and protein carbonylation and found that all of these markers of oxidative stress were elevated during chronological aging. Nevertheless, the observed effects were strain-independent (Fig. S5).

Chronological aging was also accompanied by a decrease in rhodamine fluorescence, which is believed to be a sign of mitochondrial membrane potential (MMP, Δψm) loss (Ludovico et al. 2001); the effects were strain-independent (Fig. S6). An age-related decrease in MMP was observed after 7 and 14 days of chronological culture (Fig. S6), while at day 21 and day 28, the MMP was comparable to that of day 14 (data not shown).

Mitochondrial membrane potential is critical for maintaining the physiological function of the respiratory chain to generate ATP, and a loss of Δψm may lead to energy depletion and, in turn, to chronological aging-dependent apoptosis. We then further investigated if chronological aging may be a stimulus that promotes RNA degradation, which is common for apoptotic stimuli. Indeed, we found CA-mediated RNA degradation (Fig. S7). All diploid mutants examined were more prone to RNA degradation compared to the BY4743 wild type, whilst the effects in a haploid state were more or less comparable between strains used (Fig. S7). However, is it difficult to discriminate between increased RNA degradation in chronologically aging cells and cell death-mediated RNA degradation. Because dead cells accumulate within the chronological culture, one can speculate that RNA degradation may be due to CA-mediated apoptosis.

**Fig. 3:**
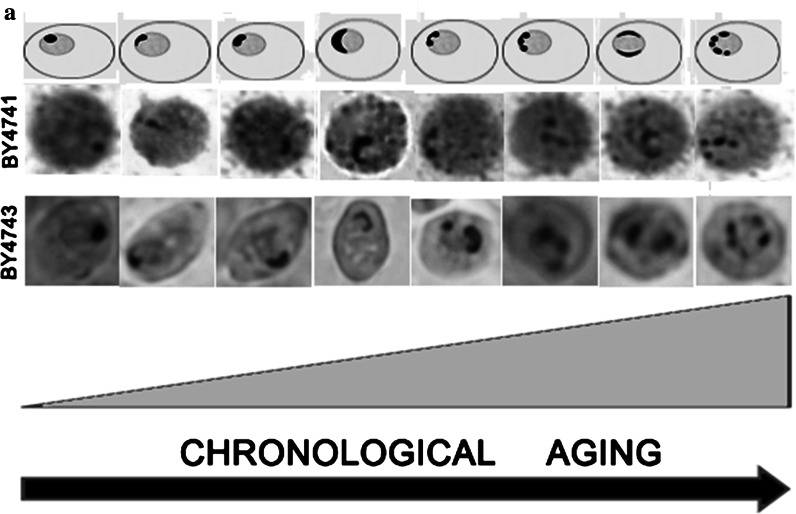

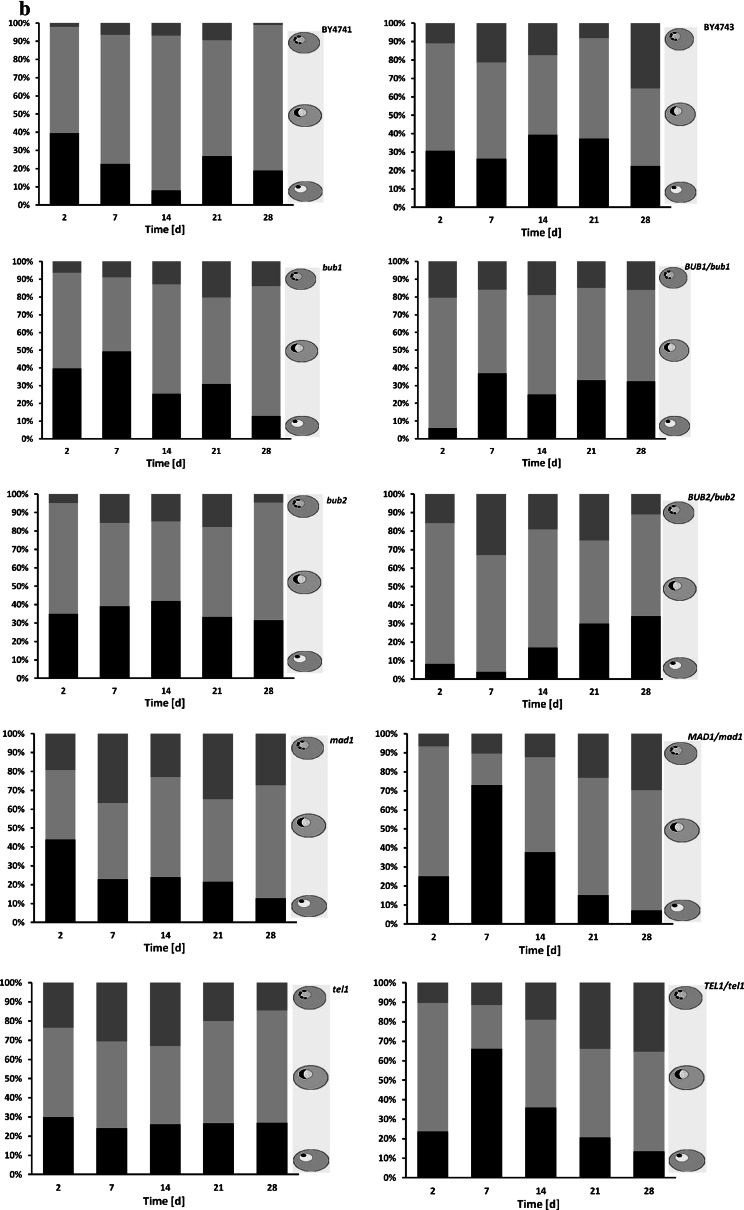
The nucleolus is fragmented during chronological aging. To visualise nucleoli, silver staining of nucleolar organiser regions (AgNOR) was performed and nucleoli images were captured with an Olympus BX61 light microscope equipped with a DP72 CCD camera and Olympus CellF software. **a** A scheme showing CA-mediated changes in nucleolar morphology. A general tendency is presented (both schematic and microscopic representations of the haploid and diploid wild type strain nucleoli are shown). **b** Graphs showing the percentage of normal (*black*), increased (*light grey*) and fragmented (*dark grey*) nucleoli in chronologically aging haploid strains (*left*) and diploid strains (*right*). A total of 100 cells were analysed, and their nucleolus morphological type was determined [%]. The results represent the mean from at least three independent experiments

Secondly, we were interested in determining if chronological aging may be accompanied by changes in nucleolus architecture and activity, which have been previously shown for replicative aging (Sinclair et al. 1997). We analysed the nucleolar shape, size and nucleolus/nucleus ratio by silver staining of nucleolar organiser region. Three main nucleolus morphological types were revealed during CA, namely the “normal” (typical) nucleolus (first schematic picture in Fig. 3a), larger nucleolus (fourth schematic picture in Fig. 3a) and the fragmented nucleolus (eighth schematic picture in Fig. 3a); these morphological types were scored [%]. Quantitative analysis demonstrated an increase in nucleolus fragmentation during chronological aging (Fig. 3b).

In the haploid state, differences in CA-mediated nucleolus fragmentation between the wild type and mutants were more pronounced than in the diploid state (Fig. 3b). Nevertheless, the effect was observed in all strains examined, both haploid and diploid, and both wild type and mutants. The percentage of fragmented nucleoli reached from 20 % to almost 40 % of the total nucleoli examined during chronological aging in haploid mutants; the *mad1* and the *tel1* mutants being the most susceptible to CA-mediated nucleolar changes (Fig. 3b). Moreover, the level of nucleolus fragmentation was augmented at day 2 of culture (control conditions) in all haploid mutants compared to the wild type (Fig. 3b). In some strains the accumulation of cells with increased nucleolus was also evident (Fig. 3b).

**Fig. 4:**
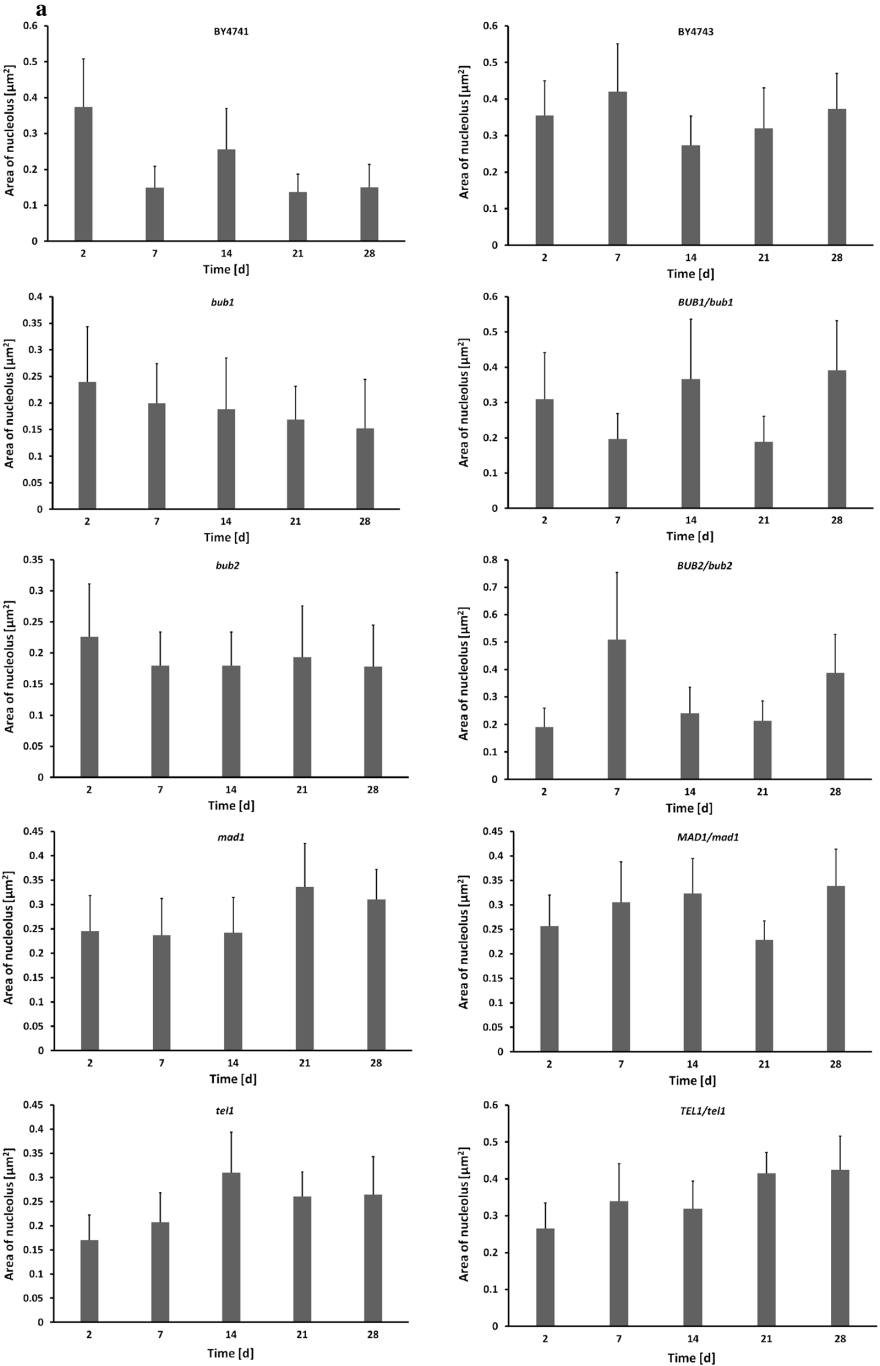

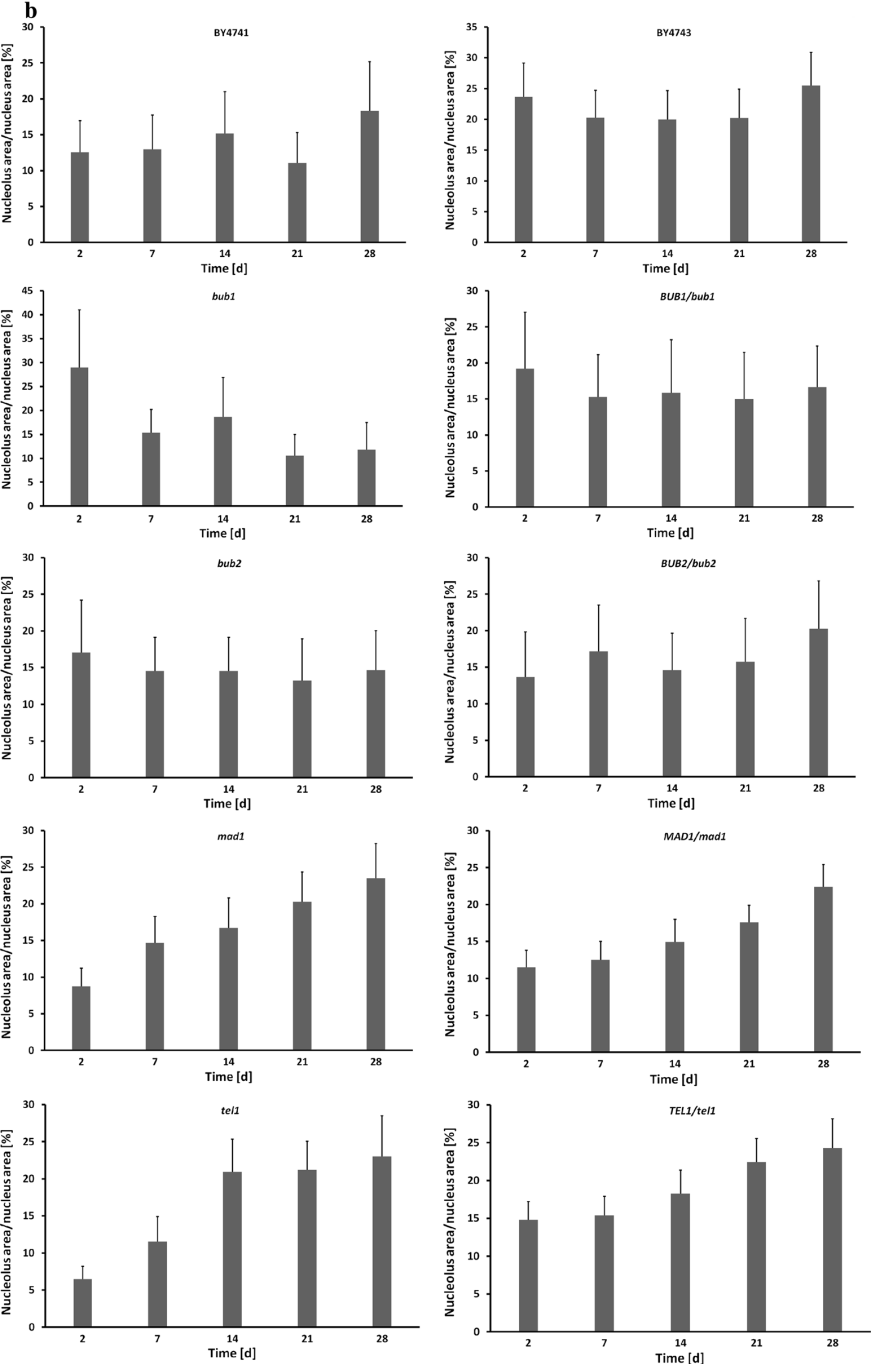
CA-mediated changes in nucleolus size and nucleolus/nucleus ratio. To visualise nucleoli, silver staining of nucleolar organiser regions (AgNOR) was performed and nucleoli images were captured with an Olympus BX61 light microscope equipped with a DP72 CCD camera and Olympus CellF software. The nucleolus size and nucleolus/nucleus ratio were calculated using ImageJ software http://rsbweb.nih.gov/ij/. **a** Nucleolus size of chronologically aging haploid strains (*left*) and diploid strains (*right*). **b** Nucleolus/nucleus ratio of chronologically aging haploid strains (*left*) and diploid strains (*right*)

Using a morphometric method, we measured nucleolus size and calculated the nucleolus/nucleus ratio during chronological aging. In the case of the *mad1* and *tel1* mutants, as well as the *MAD1/mad1* and *TEL1/tel1* cells, an increase in nucleolus size and nucleolus/nucleus ratio was the most evident and proportional to their chronological age (Fig. 4). In contrast, nucleolus size and nucleolus/nucleus ratio were diminished in the *bub1* background (Fig. 4), which may contribute to previously observed decrease in both the CLS and RLS of cells lacking *BUB1* gene in the haploid state (Figs. 1, 2). As one can observe from Fig. 2 (CLS to RLS assay), the RLS of the *tel1* mutant at day 7 was increased compared to its RLS at day 2, and the RLS of the *mad1* mutant at day 7 was comparable to the RLS at day 2. It is clear that some correlations between RLS and nucleolus size can also be found. Moreover, there were no statistically significant differences between the RLS of diploid cells showing no obvious relationship between RLS and nucleolus size.

**Fig. 5:**
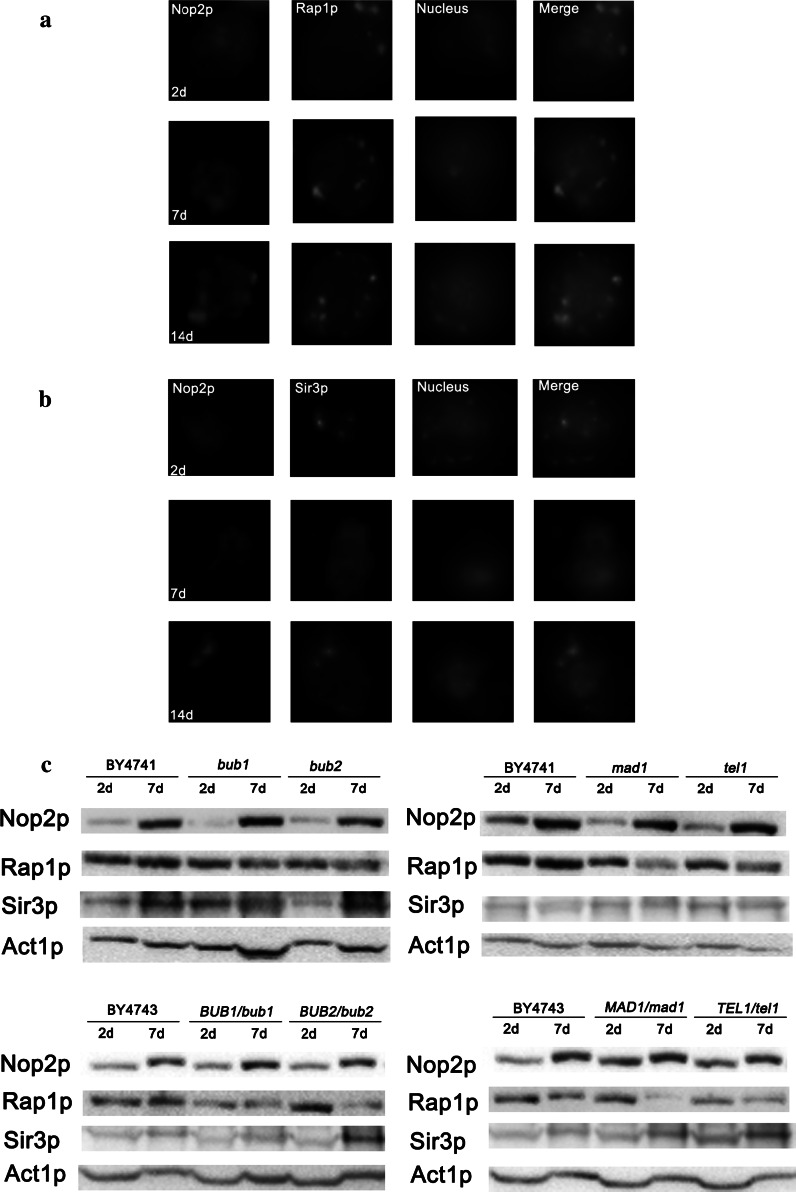
Relocation of Rap1p (**a**) and Sir3p (**b**) from telomeres to the nucleolus and changes in expression patterns of Rap1p, Sir3p and Nop2p (**c**) during chronological aging. **a, b** After 2, 7 and 14 days, Rap1p (**a**) and Sir3p (**b**) immunostaining was performed (*green*). DNA was visualised using DAPI staining (*blue*). Immunostained cells were captured with an Olympus BX61 light microscope equipped with a DP72 CCD camera and Olympus CellF software. Nop2p was used as a nucleolar marker (*red*). Typical micrographs of haploid wild type BY4741 are shown. **c** After 2 and 7 days, changes in the expression of Rap1p, Sir3p and Nop2p were revealed with western blotting. For the loading control, an antibody against actin was used. The chemiluminescence signal was detected using an ECL Plus Western Blotting Detection System (GE Healthcare) and G:BOX imaging system (Syngene). *Top* haploid strains, *bottom* diploid strains. (Color figure online)

Next, we monitored the localisation of two proteins involved in transcriptional silencing, namely Sir3p and Rap1p, during chronological aging, because the redistribution of the Sir complex from the telomeres to the nucleolus has been previously observed during replicative aging (Sinclair et al. 1997). With immunofluorescence, we were able to co-localise Sir3p and Rap1p signals with nucleolar Nop2p, which may be considered a nucleolar marker, during chronological aging (Fig. 5a, b).

Moreover, changes in nucleolar protein expression were revealed (Fig. 5c). Sir3p was upregulated, whilst Rap1p was downregulated during chronological aging. Decreased expression of Rap1p was more evident in the mutant cells compared to corresponding wild type cells (Fig. 5c). Changes in nucleolar protein expression may contribute to changes in Sir complex formation and in turn may promote Sir3p and Rap1p redistribution to the nucleolus during chronological aging (Fig. 5a, b). Surprisingly, Nop2p expression was heavily upregulated during chronological aging (Fig. 5c). The effect was strain-independent (Fig. 5).

**Fig. 6:**
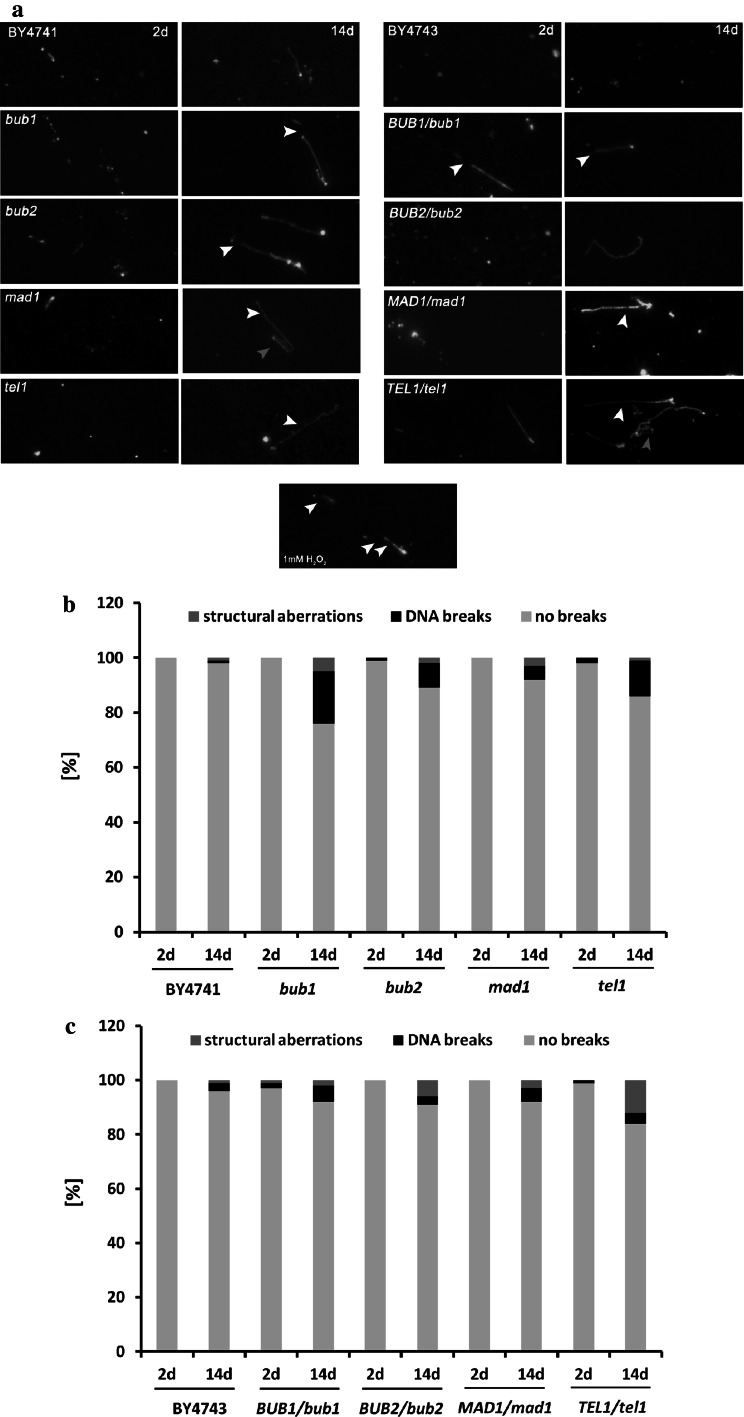
DNA breaks and structural aberrations within chromosome XII during chronological aging. After 2 and 14 days, yeast chromosomes were separated with PFGE and selected bands were cut with a razor blade. DNA breaks and structural aberrations within chromosome XII were visualised via alkaline chromosome comet assay (pH > 13). After electrophoresis, chromosomal DNA was stained with YOYO-1 (Molecular Probes) stain solution (*green*). **a** DNA breaks (*white arrowheads*) and structural aberrations (here termed as abnormal chromosome morphology) (*pink arrowheads*) were visualised using an Olympus BX61 fluorescence microscope equipped with a DP72 CCD camera and Olympus CellF software. *Left* haploid strains; *right* diploid strains; *bottom* positive control for DNA breaks, haploid wild type treated with 1 mM hydrogen peroxide for 10 min. **b, c** A total of 100 chromosomes per sample triplicate were analysed and the percentages of DNA damage and structural aberrations were calculated. **b** Haploid strains, **c** diploid strains. (Color figure online)

Because the nucleolus becomes fragmented and the functions of nucleolar proteins are affected during chronological aging, we addressed the question of whether rDNA is also unstable during CA. Chromosome XII, the largest yeast chromosome, contains the rDNA locus in yeast (Petes 1979). Thus, we decided to monitor CA-mediated chromosome XII instability. Upon conducting a chromosome comet assay, which is a modification of classical comet assay (Lewinska et al. 2014), we found DNA breaks and some minor structural aberrations (abnormal chromosome morphology) within chromosome XII during CA (Fig. 6a, white and pink arrowheads, respectively).

**Fig. 7:**
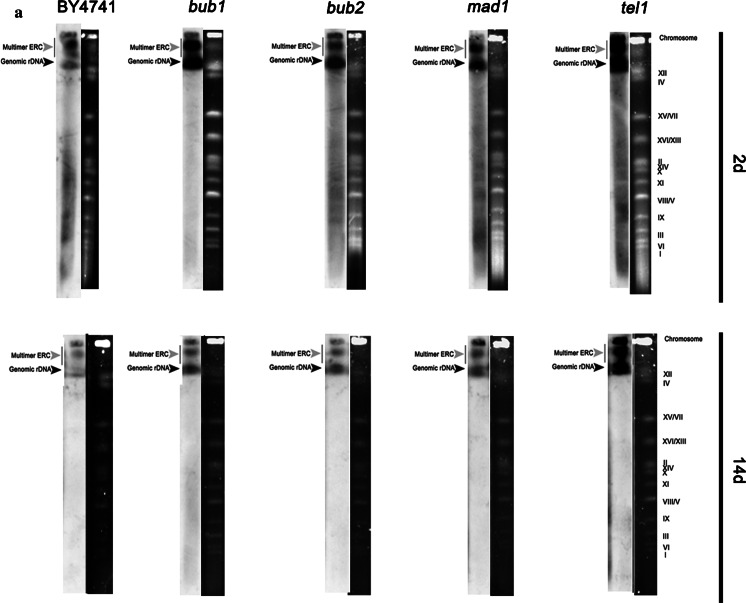

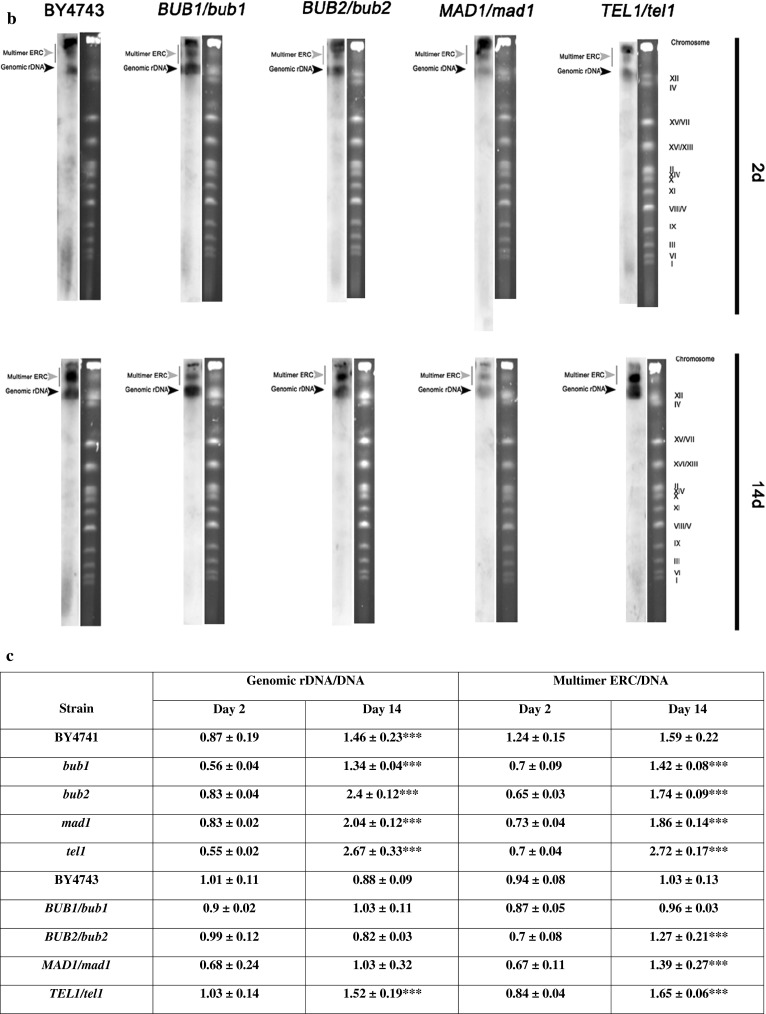
CA-associated extrachromosomal rDNA circle (ERC) accumulation. ERCs were revealed by Southern blot analysis and an rDNA specific probe. After 2 and 14 days, yeast chromosomes were separated by PFGE and were transferred onto a nylon membrane by capillary transfer. The membrane was hybridised to a digoxigenin (DIG)-labelled rDNA specific probe and rDNA was detected with an alkaline phosphatase-conjugated anti-DIG antibody. The chemiluminescence signal was detected with a substrate for alkaline phosphatase (CDP-Star) and the G:BOX imaging system (Syngene). **a** Haploid strains, **b** diploid strains. *Black arrowheads* indicate genomic rDNA, while *grey arrowheads* denote multimer ERCs. **c** Quantification of genomic rDNA and multimer ERCs. rDNA and ERCs were quantified using GelQuantNET software (http://biochemlabsolutions.com/GelQuantNET.html) using the background correction option. The amount of genomic rDNA and multimer ERCs were calculated per mean amount of DNA. *Bars* indicate SD, *n* = 3, ***p < 0.001 compared to day 2 of culture (control conditions) of a particular strain (ANOVA and Dunnett’s a posteriori test)

The DNA breakage effect was more pronounced in the mutant cells, especially in the *bub1* and *tel1* cells compared to the haploid wild type (Fig. 6b). In contrast, in diploid hemizygous mutants structural aberrations were more accentuated than DNA breaks within chromosome XII, especially in the *TEL1/tel1* cells (Fig. 6c). The accumulation of extrachromosomal rDNA circles (ERCs) is a well-established sign of rDNA instability and is considered a cause of replicative aging in yeast (Sinclair and Guarente 1997). After 14 days of chronological culture, all haploid mutants were affected by multimer ERC formation (*p* < 0.001), while the ERC level of haploid wild type BY4741 was comparable to the ERC level on day 2 of culture (Fig. 7c).

CA-mediated ERC accumulation was also observed in *BUB2/bub2*, *MAD1/mad1* and *TEL1/tel1* cells, but not in *BUB1/bub1* cells (*p* < 0.001) (Fig. 7c). Moreover, genomic rDNA was augmented during CA, which was particularly evident for all haploid cells (wild type and isogenic mutants) (*p* < 0.001) (Fig. 7c). Because chronological aging may be accompanied by rDNA instability, we were interested in determining if genomic instability may also contribute to CA. We analysed both structural and numerical aberrations. After PFGE separation, we were unable to show any gross structural abnormalities such as translocations, when cells were kept in chronological aging culture (Fig. S8). However, some minor structural aberrations within chromosome XII have been previously detected with the chromosome comet assay (Fig. 6).

**Fig. 8:**
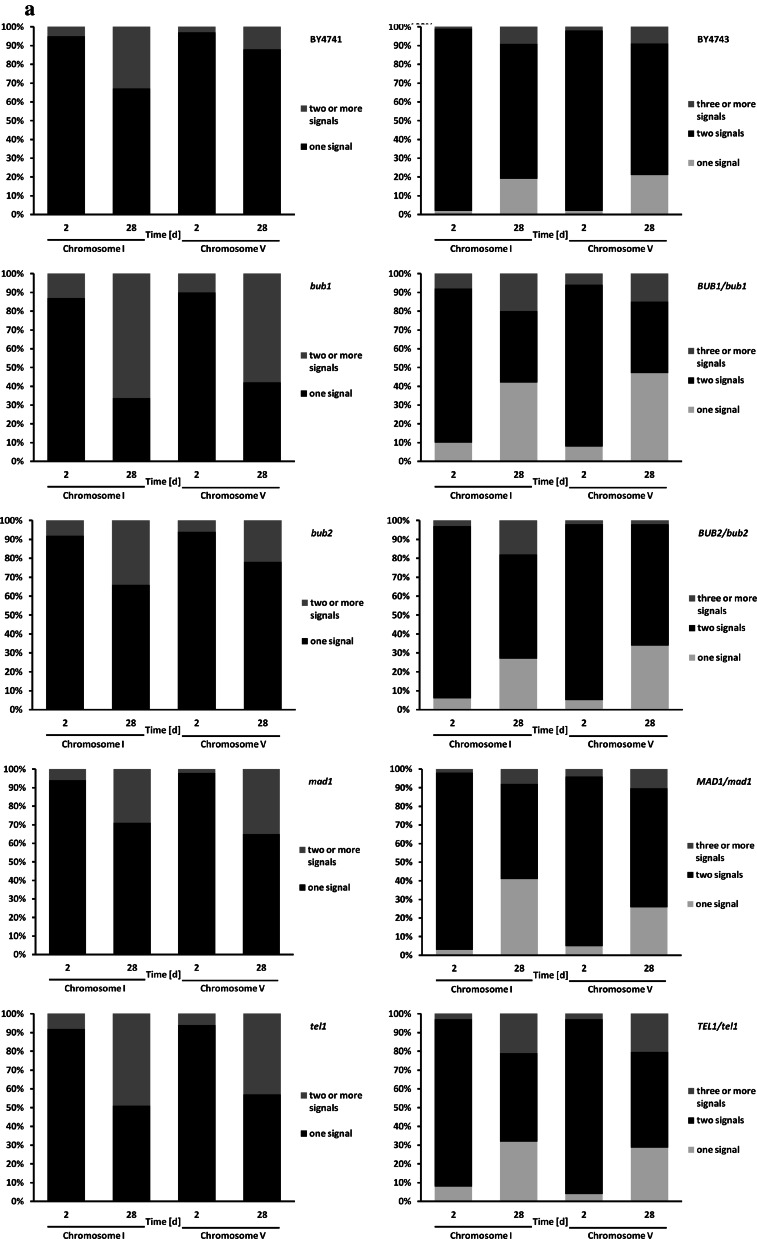

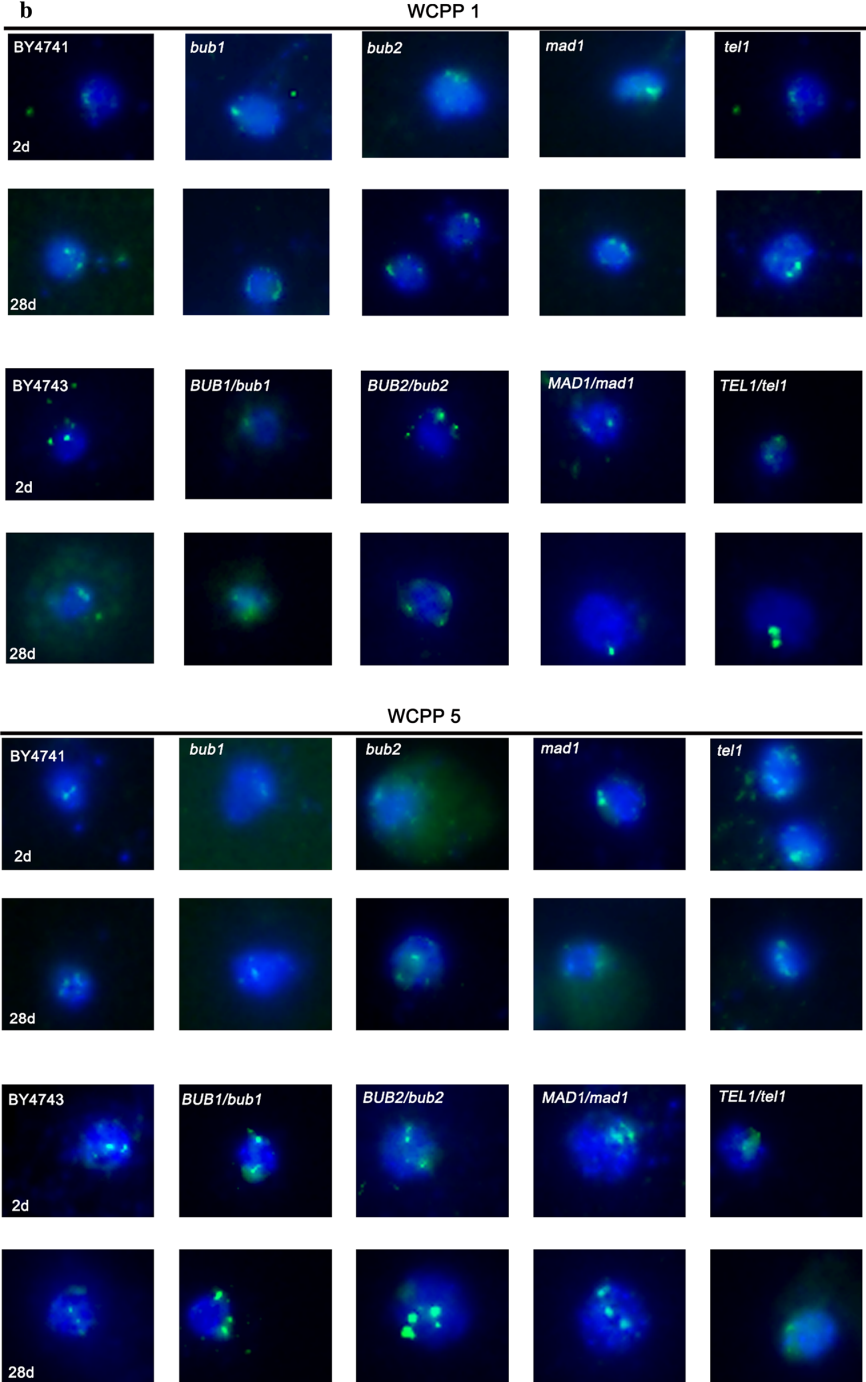
CA-induced aneuploidy. Aneuploidy was assessed using fluorescence in situ hybridisation (FISH) using whole chromosome painting probes (WCPPs). After 2 and 28 days, cells were taken from chronological aging culture and transfer to fresh YPD medium to continue mitotic growth. A biotinylated probe specific to chromosome I or chromosome V (Polish Patent Office, registration number P.404526) was added to the spheroplast-coated slide and its detection was performed with a Star*FISH^©^ Biotin Painting Kit—FITC Label (Cambio). Fluorescent signals were analysed using an Olympus BX61 fluorescence microscope equipped with a DP72 CCD camera and Olympus CellF software. **a** The frequencies of aneuploidy events (chromosome I or chromosome V): haploid strains (*left*), diploid strains (*right*). Aneuploidy events were counted and presented as a percentage of 200 total cell scores. **b** Typical micrographs showing chromosome I or chromosome V fluorescent signals are presented (*green*). DNA was visualised using DAPI staining (*blue*). (Color figure online)

Because cells in chronological aging culture are maintained in a non-dividing state, we transferred cells at indicated time points to fresh medium for growth continuation and found that surviving cells were affected by whole chromosome aneuploidy (Fig. 8).

We selected two chromosomes, namely small chromosome I and medium chromosome V, for aneuploidy analysis and created a ranking of the most aneuploidy-prone haploid mutant strains during CA: *bub1* > *tel1* > *bub2* > *mad1* for chromosome I aneuploidy and *bub1* > *tel1* > *mad1* > *bub2* for chromosome V aneuploidy (Fig. 8a). For haploid mutants, the most common aberrant signal was a disomic signal. For diploid hemizygous mutants, we observed both monosomic and trisomic signals, but monosomic signals were more frequent (Fig. 8a). The most aneuploidy-prone (both chromosome I aneuploidy and chromosome V aneuploidy) diploid hemizygous mutants were: *BUB1*/*bub1* > *TEL1*/*tel1* > *MAD1*/*mad1* > *BUB2*/*bub2* (Fig. 8a). Almost all mutants, both haploid and diploid, were more susceptible to chromosomal instability compared to the corresponding wild type cells on day 2 of culture (control conditions) (Fig. 8a).

**Fig. 9:**
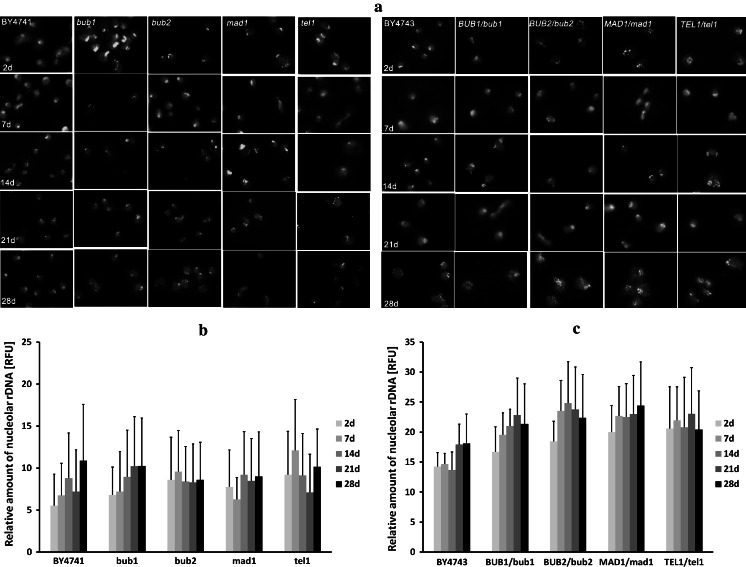
CA-mediated changes in nucleolar rDNA content. Because rDNA is located on chromosome XII, we used a chromosome XII-specific painting probe and FISH to visualise the rDNA locus and nucleolus. **a** Typical micrographs showing chromosome XII-specific fluorescent signals are presented (*green*). DNA was visualised using DAPI staining (*blue*). **b, c** Quantitation of fluorescent chromosome XII-specific signals was performed using ImageJ software http://rsbweb.nih.gov/ij/. We evaluated the integrated fluorescence density (green channel), which is the sum of all pixel values within the marked area of each cell analysed and equivalent to the product of area and mean gray value. The integrated fluorescence density is presented in relative fluorescence units (RFUs). **b** Haploid strains, **c** diploid strains. (Color figure online)

We have previously shown (Fig. 7c) that chronologically aging cells, namely all haploid strains used, accumulated more genomic rDNA than did the younger cells. Next, we aimed to examine if survivors would also accumulate more rDNA. Chronologically aging cells were transferred to fresh medium so they could re-enter the cell cycle (the same methodology as that used for the aneuploidy analyses). To visualise rDNA, a chromosome XII-specific probe was used. rDNA-specific signals were quantified and a CA-mediated increase in rDNA was observed (Fig. 9).

Nevertheless, due to high signal variability, the effects were not statistically significant. Taken together, CA-mediated aneuploidy may stimulate rDNA accumulation, which in turn may lead to nucleolus fragmentation.

## Discussion

The mechanisms of CLS regulation seem complicated and most likely involve the convergence of nutrient-sensing signalling pathways, mitochondrial respiratory capacity and stress responses (Longo et al. 2012). Because stationary phase cells kept in rich YPD medium were suggested to not undergo an aging program (Ashrafi et al. 1999) involving alterations in nucleolar architecture, the redistribution of the Sir transcriptional silencing complex and ERC accumulation (Sinclair et al. 1997; Sinclair and Guarente 1997), CA-mediated changes at the nucleolar level were not investigated. In the present study, using SDC medium containing 2 % of dextrose as the gold standard CLS assay, we have reconsidered the impact of the nucleolus on chronological aging. We were especially interested in determining if nucleolar dysfunction can promote genome instability and, in turn, affect CLS.

It is widely accepted that genome instability may contribute to cellular senescence and aging from yeast to humans (Lombard et al. 2005; Vijg and Suh 2013). We selected four haploid and diploid hemizygous mutants defective in cell cycle checkpoint control proteins, namely Bub1p, Bub2p, Mad1p and Tel1p, to create a cellular environment potentially prone to genomic instability and study correlations between aberrant nucleolus state, aneuploidy and lifespan, if any, during chronological aging. Mad1p–Bub1p–Bub3p complex formation is essential for proper spindle checkpoint function in yeast (Brady and Hardwick 2000). Bub1, a multi-task protein kinase, has also been shown to play a role in the phosphorylation of the conserved serine 121 of histone H2A in fission yeast *Schizosaccharomyces pombe*, which prevents chromosomal instability (Kawashima et al. 2010). The mouse homolog of yeast Bub1p, BubR1, was found to protect against aneuploidy and cancer (Baker et al. 2013), whilst mutations in the human *BUB1* homologues have been linked with several types of cancer (Cahill et al. 1998; Yamaguchi et al. 1999). Two yeast kinases, Tel1p (homolog of mammalian ATM kinase) and Mec1p (homolog of mammalian ATR kinase), are involved in the DNA damage/S-phase checkpoint and in telomere length regulation (Harrison and Haber 2006; Morrow et al. 1995; Ritchie et al. 1999). Chromosome rearrangements and aneuploidy were found in yeast cells lacking both Tel1p and Mec1p (McCulley and Petes 2010). Using a different methodology, the CLS of the *bub1* cells was found to be shorter compared to that of the corresponding wild type and their cell viability was much more affected. Moreover, we were able to observe the regrowth/gasping of chronologically aging cells when cell aliquots were taken late during the CLS experiments; regrowth was the most manifested result in the case of the *mad1* and *tel1* mutants. Regrowth, a common phenomenon in the CLS experiments, is considered an adaptive response of the cell subpopulation that is able to escape from quiescence and re-enter the cell cycle (Fabrizio et al. 2004; Longo et al. 2012).

There are a limited number of papers on CLS-dependent RLS (Ashrafi et al. 1999; Murakami et al. 2012; Delaney et al. 2013). Early studies of CLS to RLS assays have demonstrated that the chronological age of cells cultured in rich YPD medium determines the RLS; this effect is mediated by an unknown mechanism (Ashrafi et al. 1999). More recently, it has been shown with more the commonly used chronological aging protocol (SDC medium containing 2 % dextrose) that medium acidification (Murakami et al. 2012), metabolic state and mitochondrial function (Delaney et al. 2013) during the non-dividing period of CA may limit the subsequent RLS. We were interested in determining if chronological age may also affect RLS in our laboratory conditions using haploid and hemizygous diploid mutants and their corresponding wild type cells. Indeed, using diploid strains, we found a CA-dependent, and a more or less strain-independent, reduction in RLS. However, a link between CLS and RLS was less evident when haploid strains were micromanipulated. The only exception was the CLS-dependent RLS of the haploid *bub1* cells and the RLS of the *bub1* disruptant were dramatically decreased compared to the RLS of the BY4741 wild type.

Because oxidative stress is a well-established determinant of chronological aging (Longo et al. 1996; Jakubowski et al. 2000; Herker et al. 2004), we monitored oxidative stress parameters such as reactive oxygen species (ROS) levels in the culture medium, intracellular superoxide production and protein carbonylation during CA. Indeed, extracellular and intracellular redox equilibrium was affected during chronological aging, but the effects were strain-independent. The role of ROS during CA is much more complex. ROS may be considered signalling molecules that regulate CLS, which has been previously shown for superoxide-related apoptosis, resulting in the survival of the fittest and best adapted cells at the expense of ROS-damaged and old cells (Herker et al. 2004; Fabrizio et al. 2004). Moreover, reduced TOR signalling-dependent CLS extension has been reported to be mediated by adaptive mitochondrial ROS signalling (an increase in mitochondrial membrane potential and mitochondrial ROS production) (Pan et al. 2011). In our experimental conditions, an age-related and strain-independent decrease in rhodamine fluorescence was observed, which may suggest a loss of mitochondrial membrane potential (MMP) (Ludovico et al. 2001). However, one cannot exclude a decrease in mitochondrial mass during CA, which may also affect rhodamine fluorescence (Petit et al. 1996). Indeed, a loss of mitochondrial mass without a decrease in MMP during the aging of human epidermal cells was previously reported (Maftah et al. 1994).

In general, chronologically aging cells are prone to apoptotic cell death (Rockenfeller and Madeo 2008). It has been reported that different stimuli inducing apoptosis in yeast (e.g. oxidative stress) may also promote RNA degradation (Mroczek and Kufel 2008). Chronologically aging cells were more susceptible to rRNA specific degradation (25S and 5.8S) than were the control cells (Mroczek and Kufel 2008). Moreover, cell viability and survival were found to be related to the level of rRNA (Mroczek and Kufel 2008). We found that chronological aging was also associated with total RNA degradation and diploid mutant cells were more affected by RNA degradation than the corresponding wild type cells. In contrast, effects in the haploid state were more or less comparable between the different strains used. Because cell death is a common phenomenon during chronological aging, one can speculate that RNA degradation may be due to CA-mediated apoptosis.

In spite of certain shared molecular features of replicative and chronological aging in yeast (Kaeberlein 2010; Longo et al. 2012) and the rDNA instability-based model of replicative aging (Sinclair and Guarente 1997), CA-mediated changes in nucleolar architecture and activity have not been investigated. In the present study, we conducted a comprehensive analysis of nucleolus state, rDNA instability and overall genomic instability during chronological aging. To strengthen our results, both haploid and diploid yeast strains were used. We found CA-mediated nucleolus fragmentation and changes in nucleolus size and changes in the nucleolus/nucleus ratio. Moreover, Sir3p and Rap1p, which are involved in transcriptional silencing, were relocated to the nucleolus and their expression patterns were changed. The upregulation of Sir3p and downregulation of Rap1p may affect the formation of the Sir transcriptional silencing complex leading to protein redistribution from the telomeres to the nucleolus. The RA-mediated redistribution of silencing proteins from the telomeres to the nucleolus is thought to be an adaptive response, and the rDNA locus is considered to be a hypothetical *AGE* locus (Kennedy et al. 1997). Perhaps, the relocation of silencing proteins to the nucleolus may have a similar protective function against CA-induced detrimental changes at the rDNA locus.

Surprisingly, Nop2p, being a nucleolar marker, was heavily upregulated during CA. Nop2p has been reported to be homologous to a human proliferation-associated nucleolar protein, p120. When overexpressed, it is known to promote changes in nucleolar morphology such as fragmentation (de Beus et al. 1994). Nop2p upregulation may also contribute to CA-mediated nucleolus fragmentation. Conversely, Nop2p has been found to be a RNA methyltransferase able to methylate C2870 of the 25S rRNA (Sharma et al. 2013). Although its biological substrates and functions are largely unknown (Sharma et al. 2013), one cannot exclude the possibility that Nop2p may have a role as a methylating agent during chronological aging in yeast.

Next, we focused more on rDNA stability because silencing proteins were targeting to nucleolus to exert putative protective effects on the rDNA locus. DNA breaks within chromosome XII, which contains the rDNA locus in yeast, with increasing chronological age were revealed using the chromosome comet assay. Chromosome XII instability may be mediated by previously observed intracellular redox imbalance. Moreover, with a specific chromosome XII painting probe, we were able to visualise the nucleolus and rDNA locus and found that nucleolar rDNA was slightly increased during CA. This finding may indicate that CA-induced breaks within rDNA are subjected to DNA repair processes, e.g., by homologous recombination (HR) and/or non-homologous end joining (NHEJ). It has been shown that extrachromosomal rDNA circles (ERCs) accumulate in mother cells and are a cause of replicative aging (Sinclair and Guarente 1997). The formation of ERCs is considered a sign of high recombination rate-mediated rDNA instability (Sinclair and Guarente 1997). RLS may be elongated/shortened by the manipulation of rDNA recombination and, in turn, ERC production. This outcome has been demonstrated for the *fob1* mutant with stabilised rDNA recombination, reduced ERC production and RLS extension and for the *sir2* mutant with a high rDNA recombination rate, augmented ERC production and short RLS (Takeuchi et al. 2003; Defossez et al. 1999; Kaeberlein et al. 1999). However, there are also data on the lack of a correlation between ERC accumulation and cellular senescence (Heo et al. 1999; Kim et al. 1999; Hoopes et al. 2002; Merker and Klein 2002). Because cells with defects in DNA replication, DNA repair and transcription elongation may manifest an rDNA instability phenotype and lifespan shortage without ERC accumulation (Heo et al. 1999; Hoopes et al. 2002; Merker and Klein 2002), it may be postulated that rDNA instability itself may limit lifespan rather than ERC formation (Kobayashi 2008). In our experimental conditions, multimer ERCs were found to be accumulated in all haploid mutant strains at day 14 of culture, which may indicate that mutants with abnormal cell cycle checkpoint control may be affected by rDNA instability during CA. CA-mediated ERC accumulation in diploid mutants was less evident.

Recently, rDNA theory of aging has been proposed (Kobayashi 2008). rDNA has been suggested to be more prone to damage than other DNA regions and the protein-dense nucleolus has been shown to be more sensitive to protein damage during cellular senescence (Kobayashi 2008). Moreover, rDNA may have a role in the maintenance of genome integrity since rDNA may induce checkpoint control, which, in turn, may lead to the activation of repair enzymes, prevent apoptosis and tumorigenesis (Kobayashi 2008). Taken together, rDNA may sense DNA damage and protect the genome from damage (being both a “DNA damage sensor” and “shock absorber”) (Kobayashi 2008).

Next, we addressed the question of whether rDNA stability-mediated genome integrity may be affected during chronological aging, especially if whole chromosome aneuploidy may limit CLS. We analysed both structural and numerical aberrations. Gross structural abnormalities, such as translocations, did not accumulate during CA, whilst chronologically aging cells were largely affected by whole chromosome aneuploidy as estimated with chromosome I and chromosome V specific painting probes. The *bub1* haploid mutant was the most prone to aneuploidy events, which may contribute to its shortened CLS.

**Fig. 10:**
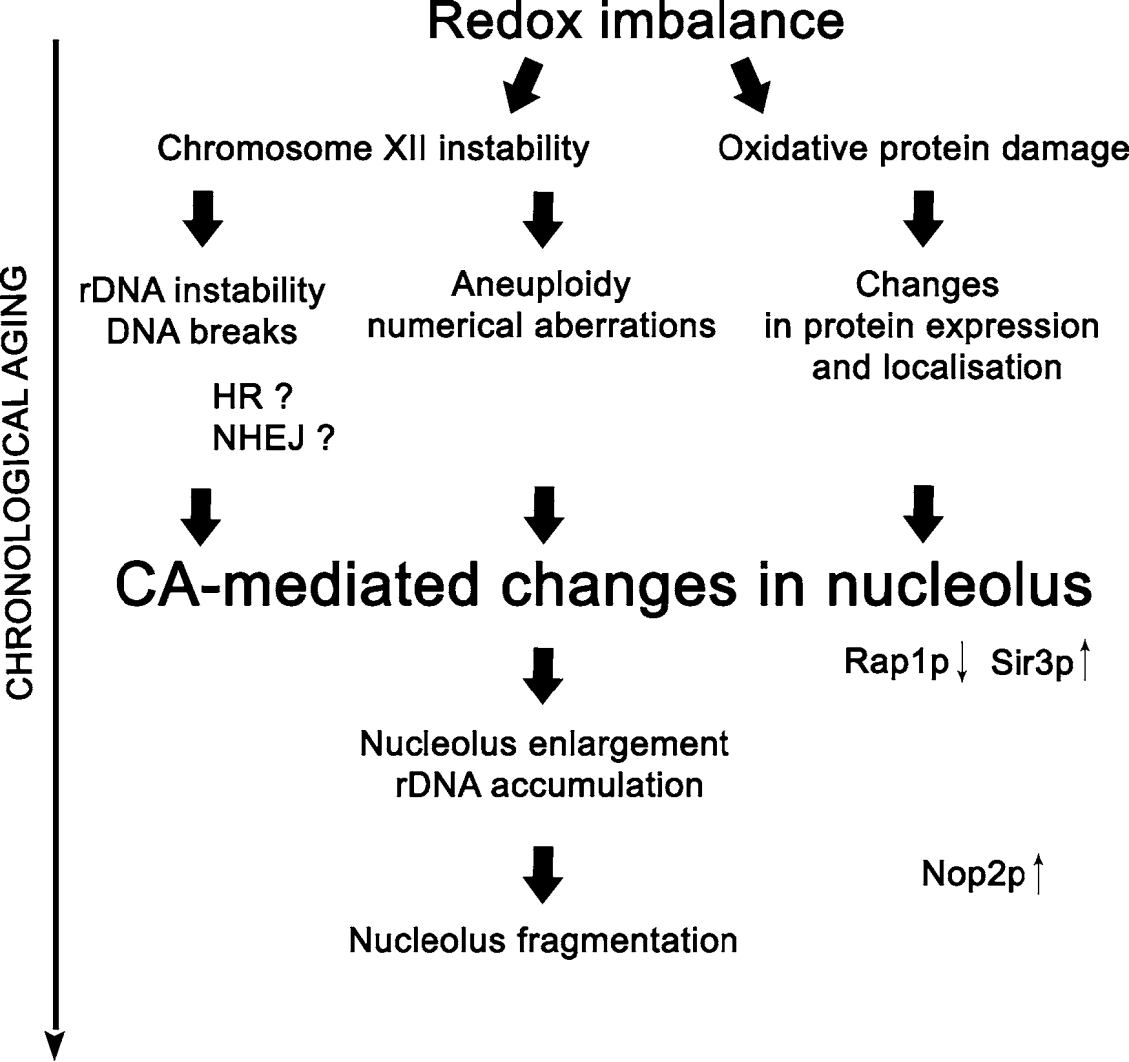
rDNA instability-based chronological aging in yeast. During CA, oxidative stress-induced chromosome XII instability may contribute to both rDNA instability and whole chromosome aneuploidy. Moreover, altered redox equilibrium may promote oxidative protein modifications leading to changes in the protein expression patterns, turnover and functions. Breaks within rDNA are subjected to DNA repair processes, e.g., by homologous recombination (HR) and/or non-homologous end joining (NHEJ), which may result in altered nucleolar architecture and activity (nucleolus enlargement, rDNA accumulation). Finally, CA-mediated nucleolus fragmentation may be a consequence of nucleolus enlargement and/or Nop2p upregulation

In conclusion, we showed for the first time that chronological aging is a stimulus that modulates nucleolus architecture and activity (nucleolus fragmentation, changes in size and nucleolus/nucleus ratio, redistribution of silencing proteins to the nucleolus and their altered expression patterns), which may be mediated by intracellular redox disequilibrium. Our data are in agreement with the rDNA theory of aging (Kobayashi 2008), because we observed CA-mediated rDNA instability as a consequence of whole chromosome XII instability. Taken together, these findings indicate that nucleolus state may be affected during chronological aging, which may promote genomic instability and in turn limit CLS, which was unequivocally observed for the *bub1* mutant. A proposed model of rDNA instability-based chronological aging in yeast is presented in Fig. 10.

**Fig. 11:**
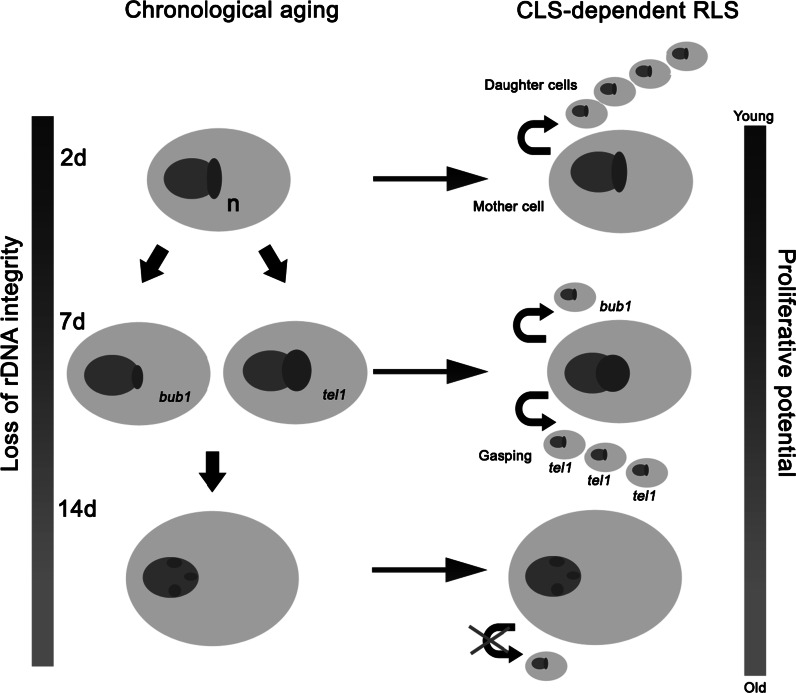
rDNA content of chronologically aging cells determines the subsequent RLS. Haploid cells lacking the *BUB1* gene were characterised by a lower amount of rDNA (nucleolus size and nucleolus/nucleus ratio) compared to the *tel1* mutant. After transferring chronologically aging cells from spent CA medium to fresh YPD medium, rDNA accumulation was more pronounced in the *tel1* mutant than in the *bub1* mutant, which in turn resulted in reduced RLS of the *bub1* cells and the gasping phenomenon of the *tel1* cells. Because similar changes in nucleolus size in diploid hemizygous mutants were not observed during CA, the subsequent RLS of diploid cells were comparable to each other. Late in the CLS experiment (day 14), a fraction of cells with fragmented nucleoli was revealed and a loss of rDNA integrity resulted in reduced RLS

Moreover, we indicated that the rDNA content of chronologically aging cells may determine the subsequent RLS (Fig. 11).

The nucleolus size of cells devoid of the *BUB1* gene in the haploid state was reduced compared to nucleolus size of the *tel1* cells during CA, which may result in a shortened RLS of the *bub1* cells. Chronologically aging cells devoid of the *TEL1* gene accumulated more genomic rDNA than did the *bub1* cells. Moreover, after transferring the *tel1* cells from spent CA medium to fresh YPD medium, more rDNA accumulation was recorded in the *tel1* cells than in the *bub1* cells (day 7 of chronological culture). This accumulation may promote the regrowth phenomenon (gasping) of the *tel1* mutant and stimulate RLS. After 14 days of CA culture, a fraction of cells with fragmented nucleoli was unable to enter the cell cycle, which was demonstrated via the reduced proliferative potential in the CLS to RLS assay.

## Electronic supplementary material

Below is the link to the electronic supplementary material.

Fig. S1 Kinetics of growth of chronologically aging cells: left panel haploid wild type strain BY4741 and isogenic *bub1*, *bub2*, *mad1* and *tel1* mutants; right panel: diploid wild type strain BY4743 and isogenic *BUB1*/*bub1*, *BUB2*/*bub2*, *MAD1*/*mad1* and *TEL1*/*tel1* mutants. After 2, 7, 14, 21 and 28 days, appropriate aliquots from CA cultures were taken for analysis. A total volume of 150 μl YPD medium with working concentration of 5×10^6^ cells/ml were cultured at 28°C and their growth was monitored turbidimetrically at 600 nm in a microplate reader every 2 h during a 12 h. Bars indicate SD, n = 3. ^***^ *p* < 0.001 compared to growth kinetics of the wild type strain (ANOVA and Dunnett’s *a posteriori* test) (DOC 804 kb)

Fig. S2 Survival of haploid wild type strain BY4741 and isogenic *bub1*, *bub2*, *mad1* and *tel1* mutants (top panel), and diploid wild type strain BY4743 and isogenic *BUB1*/*bub1*, *BUB2*/*bub2*, *MAD1*/*mad1* and *TEL1*/*tel1* mutants (bottom panel) during CA (spot assay). After 2, 7, 14, 21 and 28 days, appropriate aliquots from CA cultures were taken for analysis. Several dilutions (10^7^, 10^6^, 10^5^, 10^4^, 10^3^ cells/ml) of a yeast CA culture in a volume of 2 μl were used, inoculated on solid YPD medium and inspected after 48 h. The results shown are representative for at least three independent experiments (DOC 1381 kb)

Fig. S3 Late in the CLS experiment, a gasping phenomenon was revealed. After 21 and 28 days, appropriate aliquots from CA cultures were taken for analysis. Several dilutions (10^7^, 10^6^, 10^5^, 10^4^, 10^3^ cells/ml) of a yeast CA culture in a volume of 2 μl were used, inoculated on solid YPD medium and inspected after 48 h. Three different growth patterns of selected time points are presented (DOC 457 kb)

Fig. S4 CA-mediated cell viability (A) of haploid wild type strain BY4741 and isogenic *bub1*, *bub2*, *mad1* and *tel1* mutants (left panel), and diploid wild type strain BY4743 and isogenic *BUB1*/*bub1*, *BUB2*/*bub2*, *MAD1*/*mad1* and *TEL1*/*tel1* mutants (right panel). Cell viability was estimated with a LIVE/DEAD^®^ Yeast Viability Kit (Molecular Probes) using the standard protocol according to the manufacturer’s instructions. Percentage of live and dead cells is shown. Typically, 300 cells were used for the analysis. The results shown are representative for at least three independent experiments. B) Representative micrographs are shown: haploid wild type BY4741 (top panel), diploid wild type BY4743 (bottom panel) (DOC 880 kb)

Fig. S5 Chronological aging is accompanied by oxidative stress. A) Reactive oxygen species (ROS) level is increased in the culture medium during CA (media obtained from haploid strains - left panel, media obtained from diploid strains - right panel). After 2, 7, 14, 21 and 28 days, ROS level was measured with 2′,7′-dichlorodihydrofluorescein diacetate (H_2_DCF-DA). B) Intracellular superoxide production is augmented during CA (haploid strains - left panel, diploid strains - right panel). After 2, 7, 14, 21 and 28 days, superoxide kinetics was measured with dihydroethidium. Fluorescence intensity was monitored in a Tecan Infinite^®^ M200 fluorescence mode microplate reader. A, B) Bars indicate SD, n = 3, ^***^ *p* < 0.001 compared to day 2 of culture (control conditions) of a particular strain (ANOVA and Dunnett’s *a posteriori* test). C) Protein carbonylation is elevated during CA (haploid strains - left panel, diploid strains - right panel). After 2 and 7 days, protein carbonylation was revealed with 2,4-dinitrophenylhydrazine (DNPH) derivatisation and anti-DNP antibody (OxyBlot^™^ Protein Oxidation Detection Kit, Millipore). For every oxyblot, a negative control without DNPH derivatisation is shown (DOC 1112 kb)

Fig. S6 CA-mediated changes in mitochondrial membrane potential (MMP). After 2, 7 and 14 days, the fluorescence intensity of rhodamine G6 reflecting the mitochondrial membrane potential was monitored in a Tecan Infinite^®^ M200 fluorescence mode microplate reader. Mitochondrial membrane potential is presented as relative fluorescence units (RFUs). A) Haploid strains, B) diploid strains. Bars indicate SD, n = 3, ^***^ *p* < 0.001 compared to day 2 of culture (control conditions) of a particular strain (ANOVA and Dunnett’s *a posteriori* test). C) Typical micrographs are shown. Cells per each sample triplicate were analysed using an Olympus BX61 fluorescence microscope equipped with a DP72 CCD camera and Olympus CellF software (DOC 493 kb)

Fig. S7 CA-mediated RNA degradation. After 2, 7, 14, 21 and 28 days, RNA was isolated using an RNeasy Mini Kit (Qiagen) and RNA chip electrophoresis was performed with an Experion^™^ Automated Electrophoresis System and an Experion^™^ RNA StdSens Analysis Kit (Biorad). RQI (an RNA quality indicator) algorithm was used to assess RNA integrity by comparing the electropherogram of RNA samples to a series of standardised degraded RNA samples. RNA electropherograms were transformed to virtual gel images. Top panel: haploid strains, bottom panel: diploid strains. RNA molecular marker is also shown (Biorad) (DOC 215 kb)

Fig. S8 CA-associated structural aberrations. After 2, 7, 14, 21 and 28 days, yeast chromosomes were separated with PFGE according to the manufacturer’s instructions using a CHEF-DR^®^III Pulsed Field Electrophoresis System (Biorad). A) Haploid strains, B) diploid strains. Typical micrographs are shown (DOC 2127 kb)
