# Supplementary figures and images for: Links between nucleolar activity, rDNA stability, aneuploidy and chronological aging in the yeast Saccharomyces cerevisiae
Source: Biogerontology. 2014 Apr 8;15(3):289–316. doi: 10.1007/s10522-014-9499-y (PMC4019837; doi:10.1007/s10522-014-9499-y)

**Supplemental** Figure 1.

| 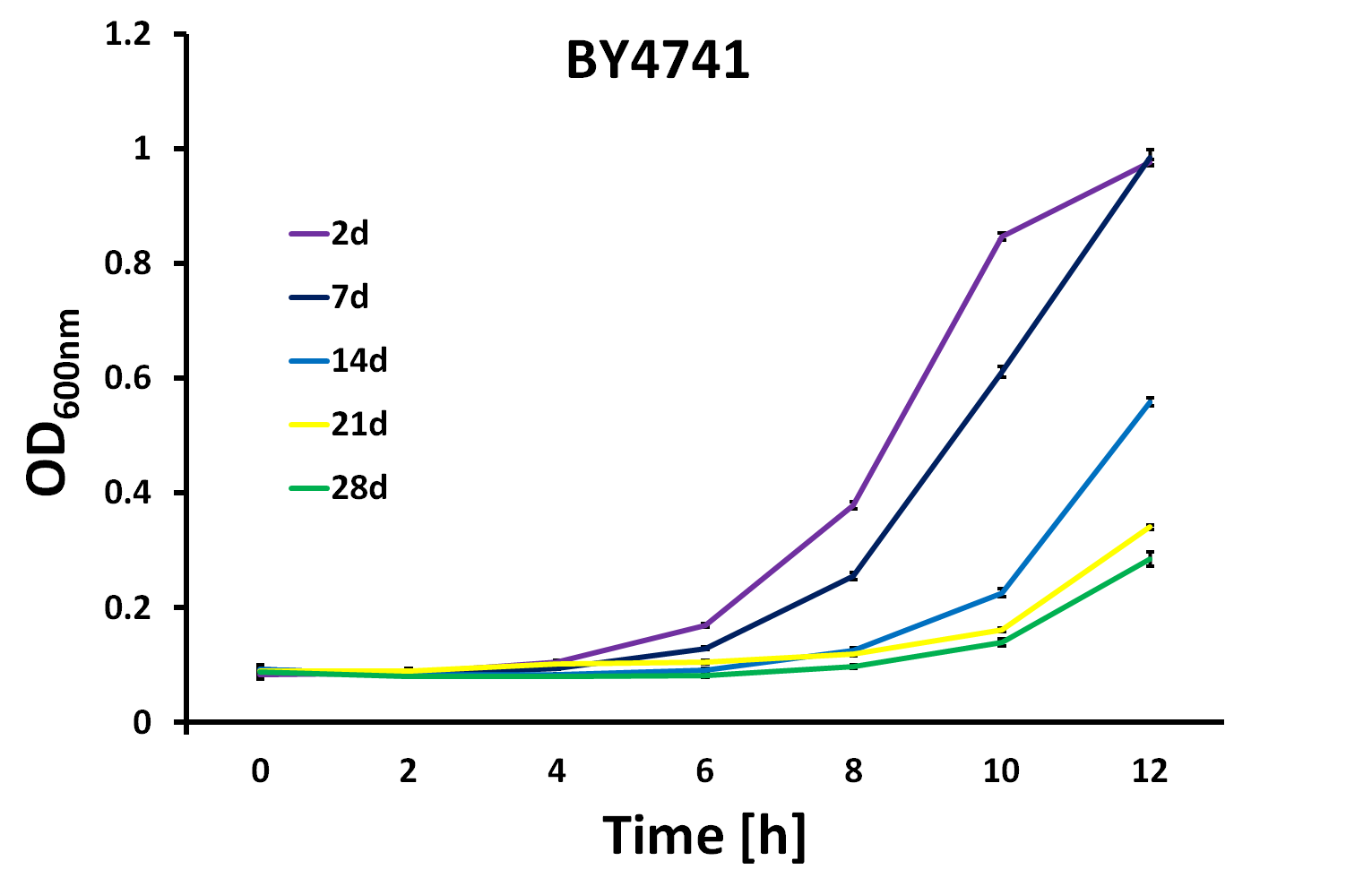 | 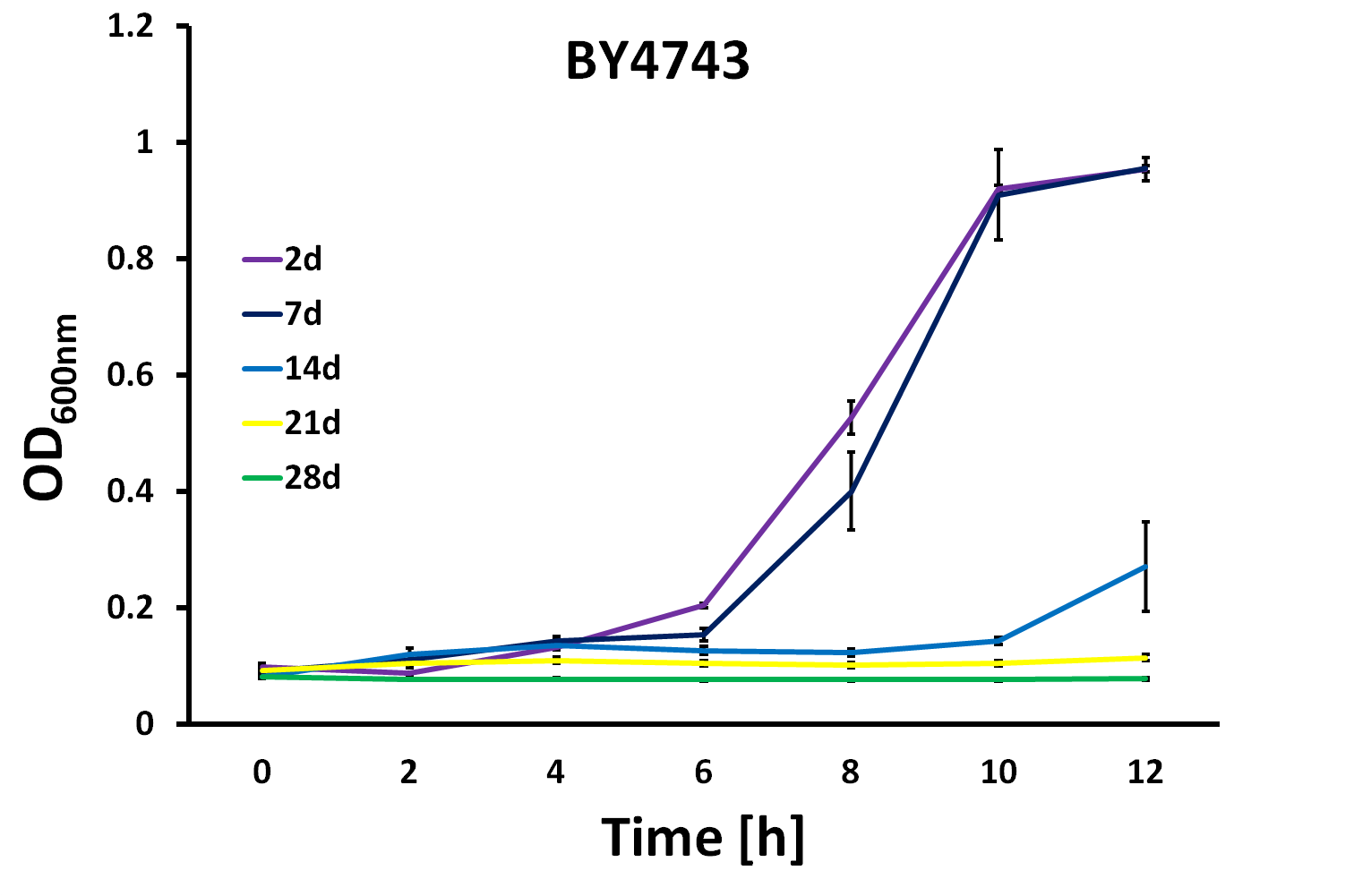 |
| --- | --- |
| 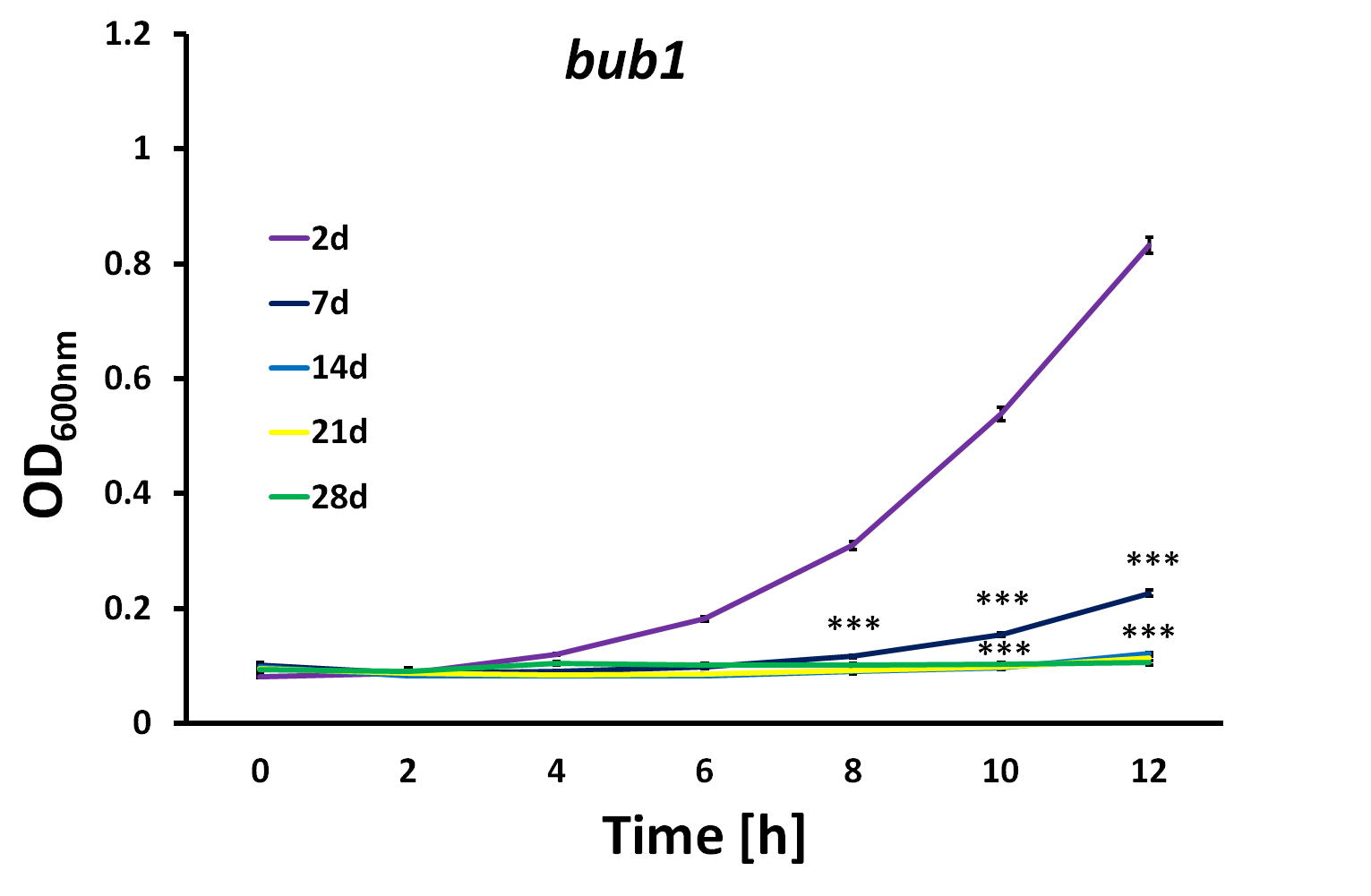 | 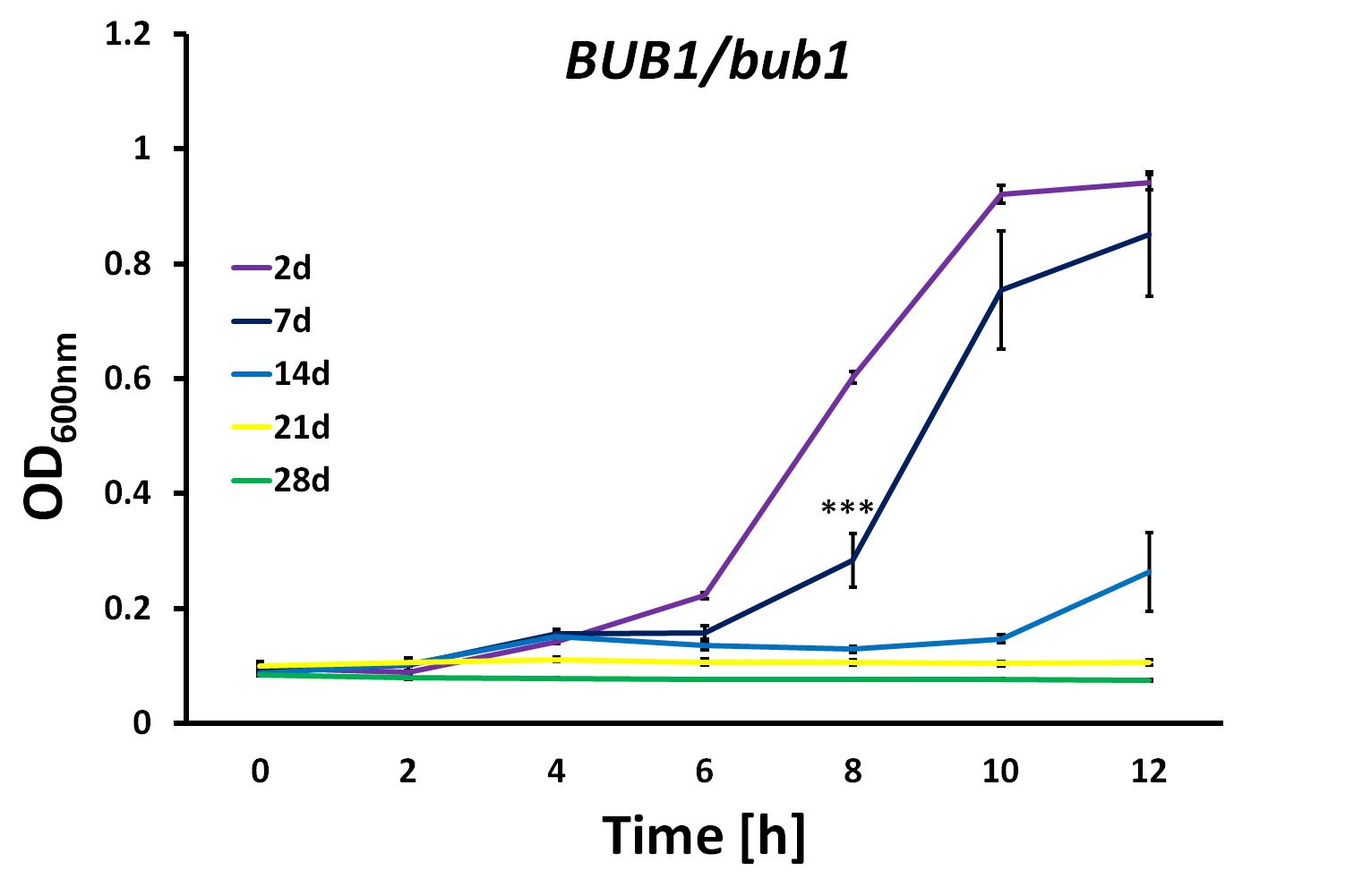 |
| 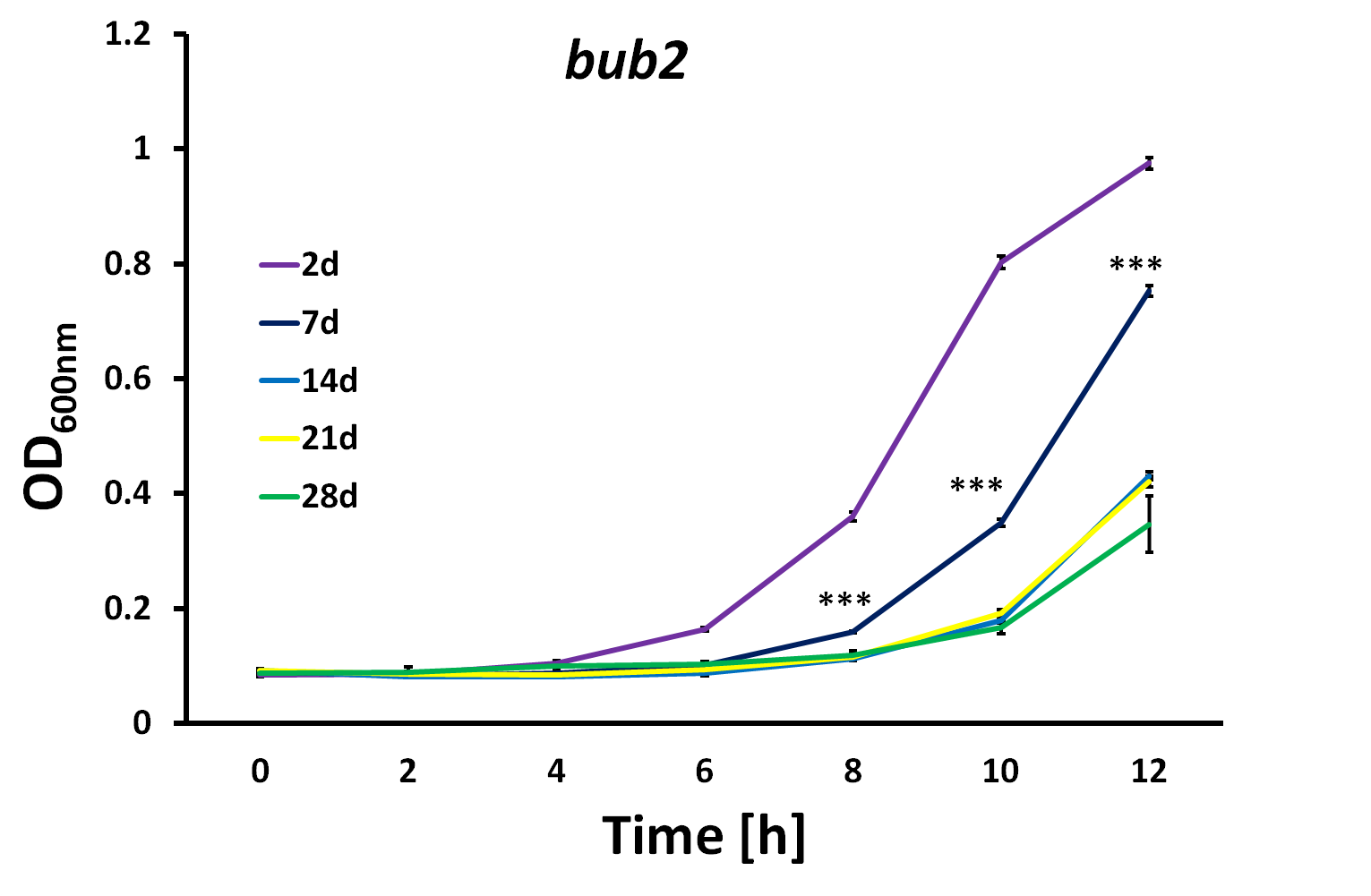 | 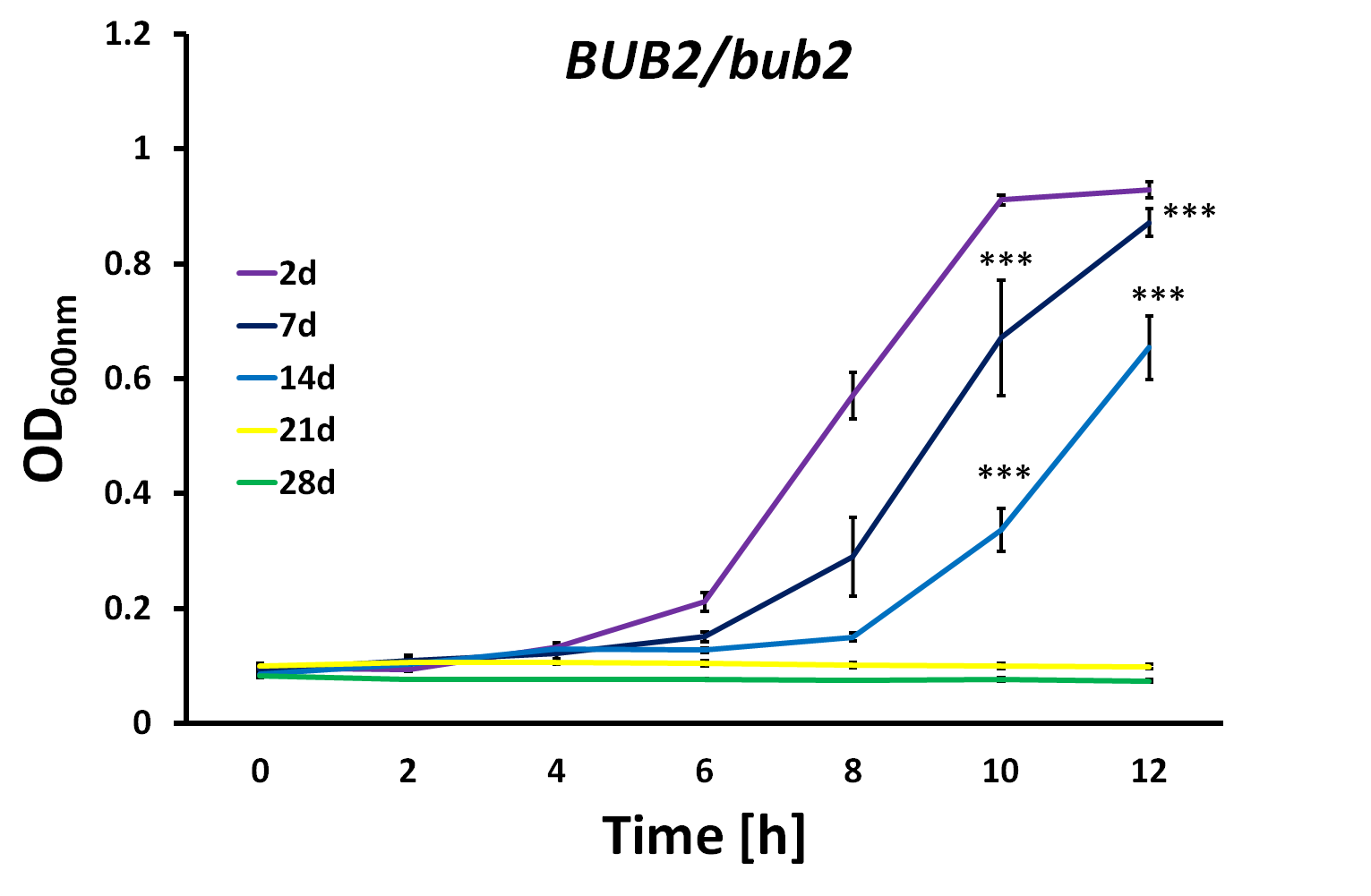 |
| 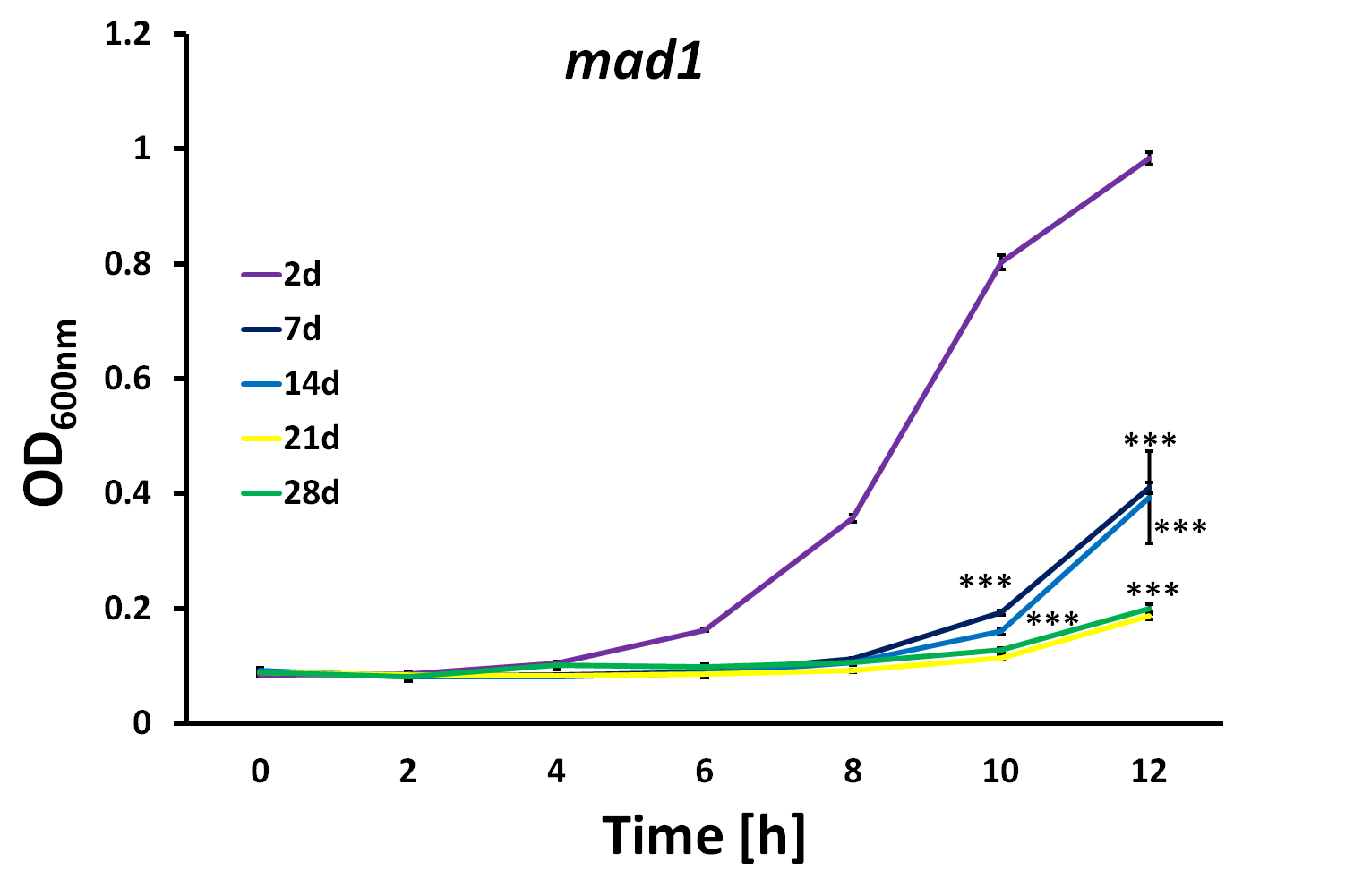 | 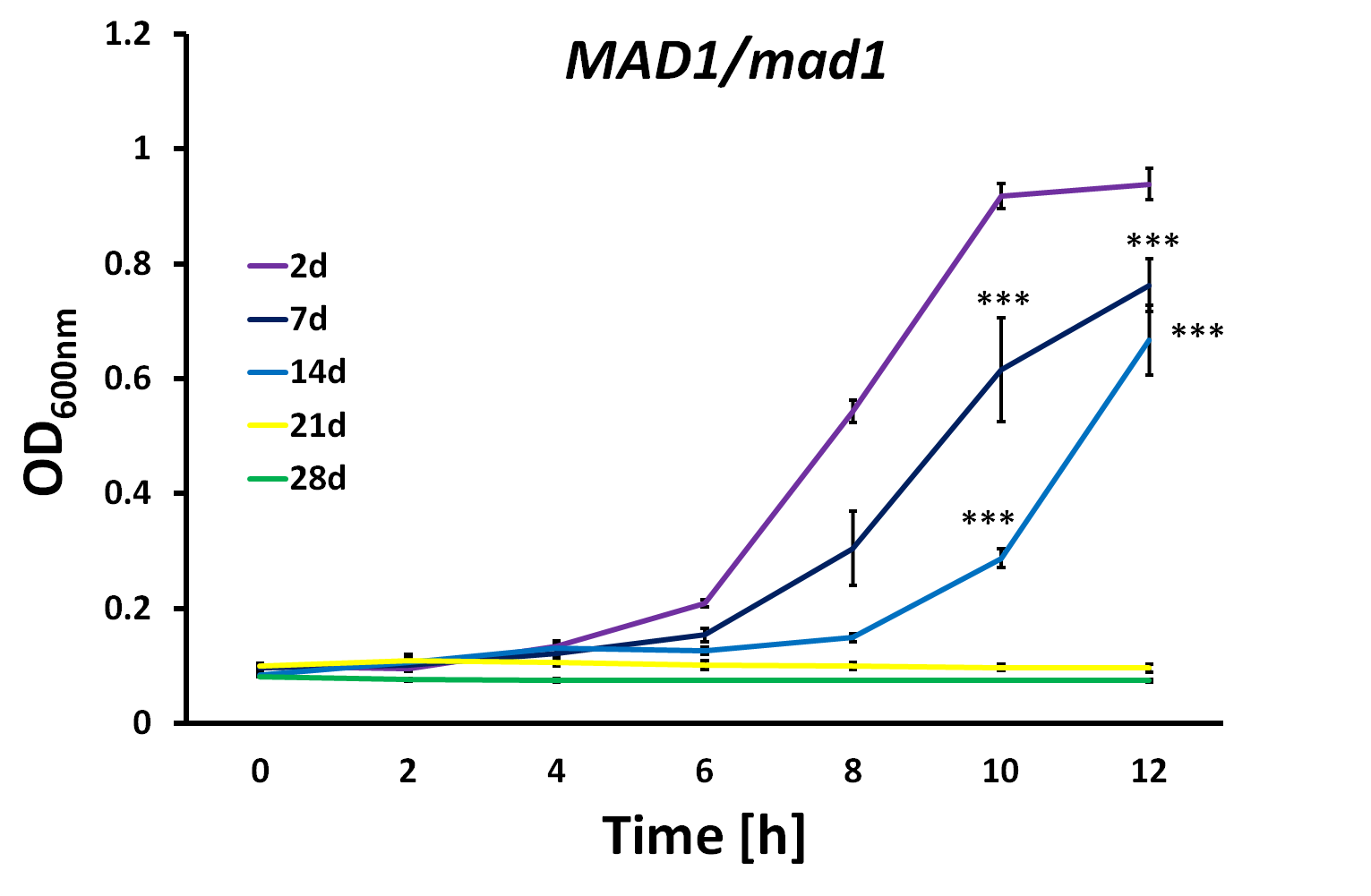 |
| 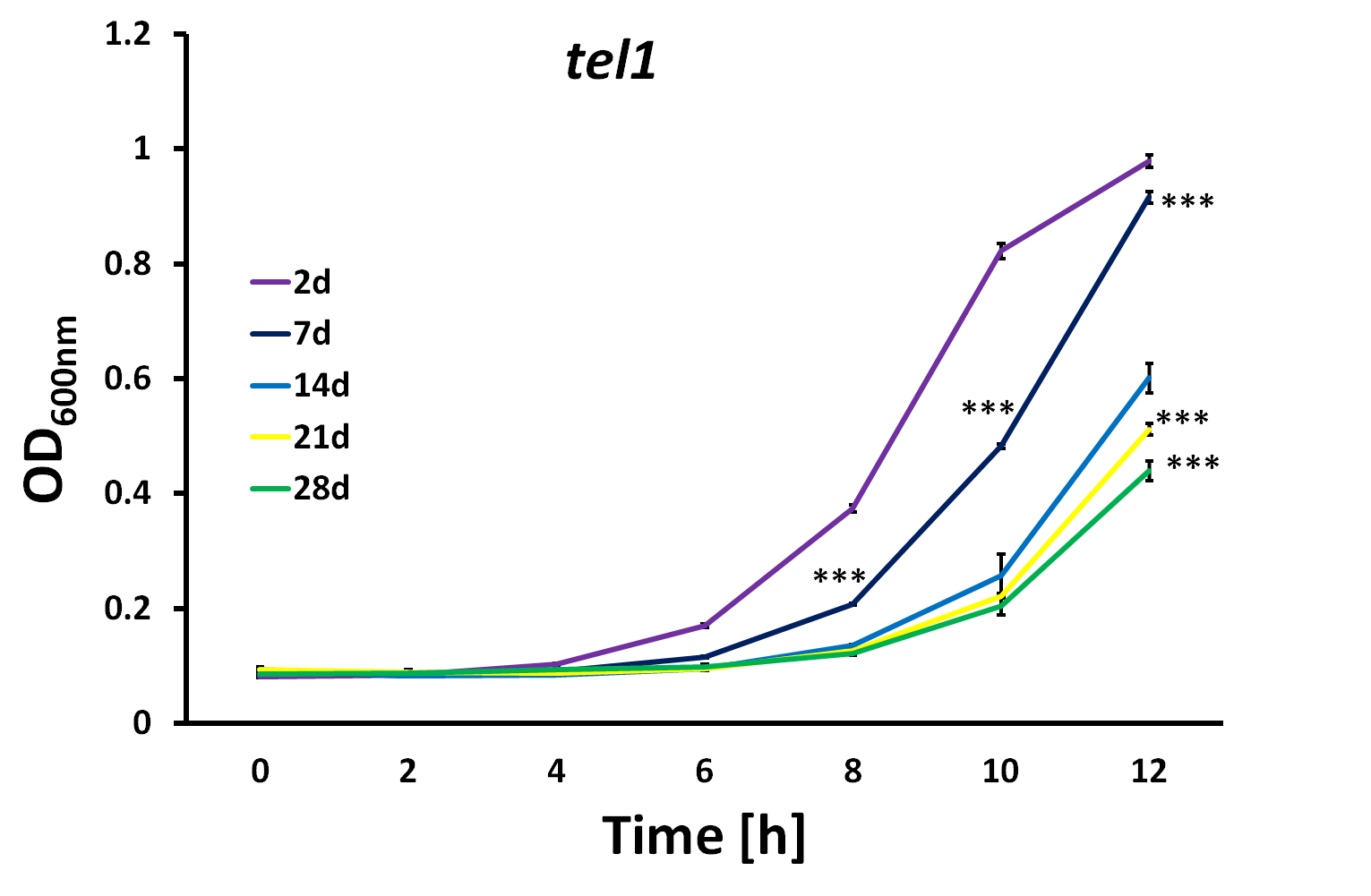 | 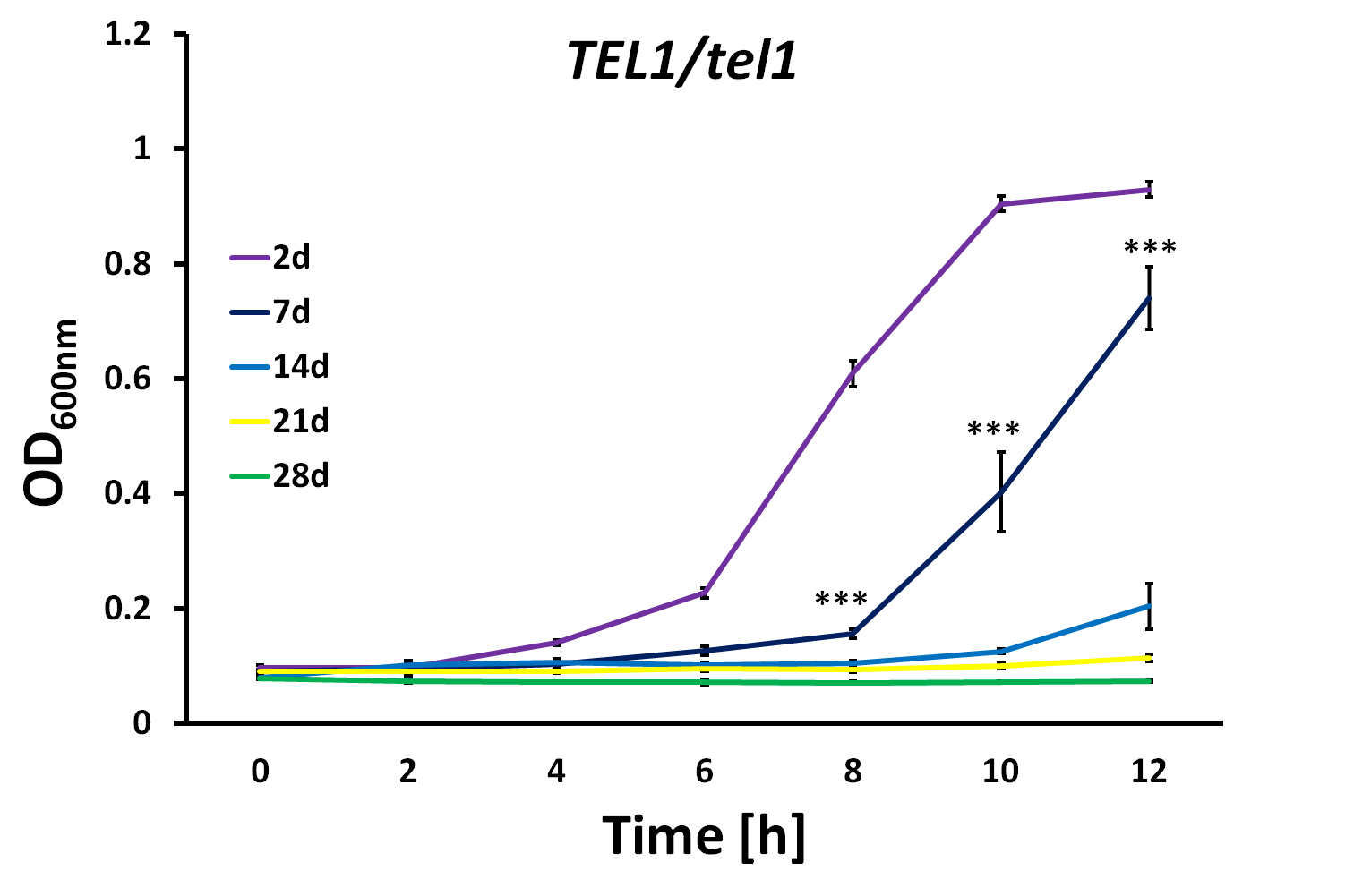 |

Supplement: Supplementary file 1 — Kinetics of growth of chronologically aging cells: left panel haploid wild type strain BY4741 and isogenic bub1, bub2, mad1 and tel1 mutants; right panel: diploid wild type strain BY4743 and isogenic BUB1/bub1, BUB2/bub2, MAD1/mad1 and TEL1/tel1 mutants. After 2, 7, 14, 21 and 28 days, appropriate aliquots from CA cultures were taken for analysis. A total volume of 150 μl YPD medium with working concentration of 5×10^6^ cells/ml were cultured at 28°C and their growth was monitored turbidimetrically at 600 nm in a microplate reader every 2 h during a 12 h. Bars indicate SD, n = 3. *** p < 0.001 compared to growth kinetics of the wild type strain (ANOVA and Dunnett’s a posteriori test) (DOC 804 kb) [file 10522_2014_9499_MOESM1_ESM.doc]

**Supplemental** Figure 2.

| 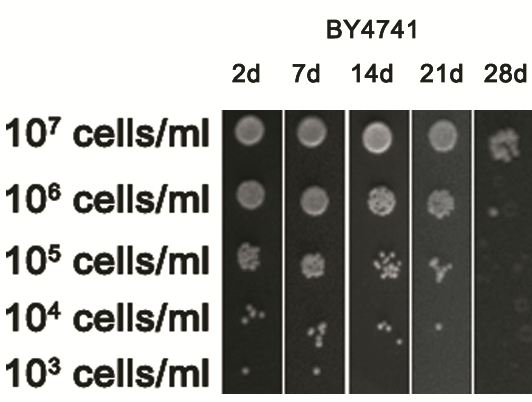 | 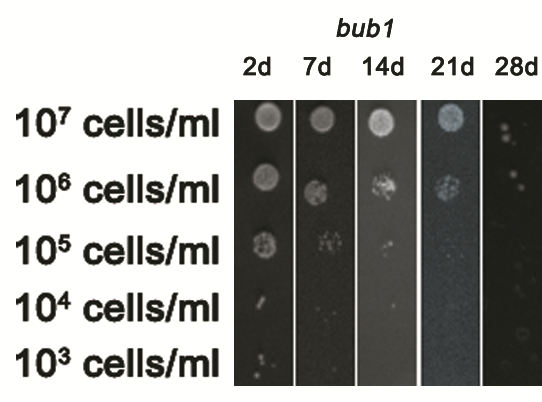 | 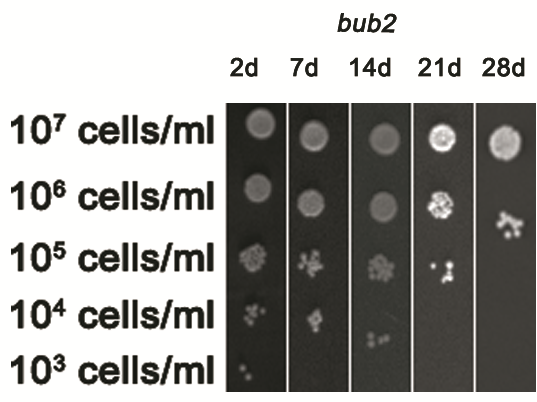 | 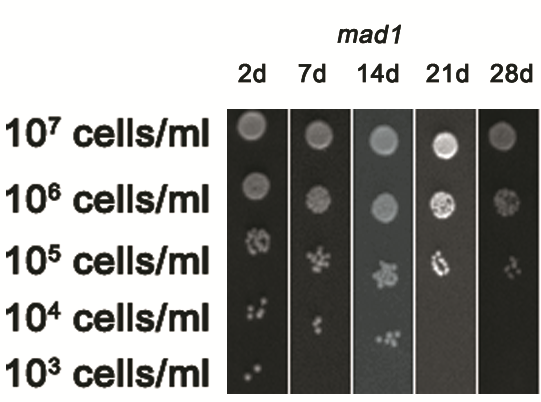 | 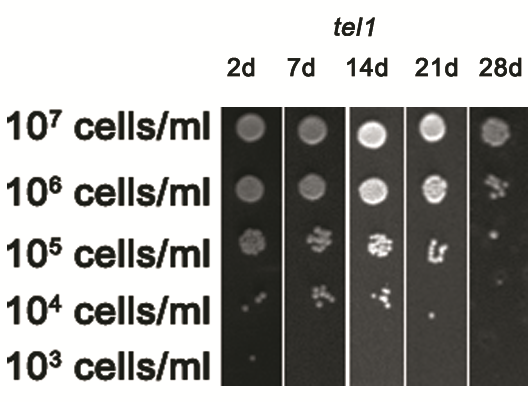 |
| --- | --- | --- | --- | --- |
| 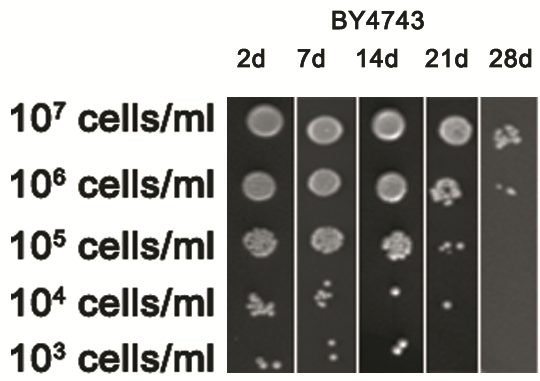 | 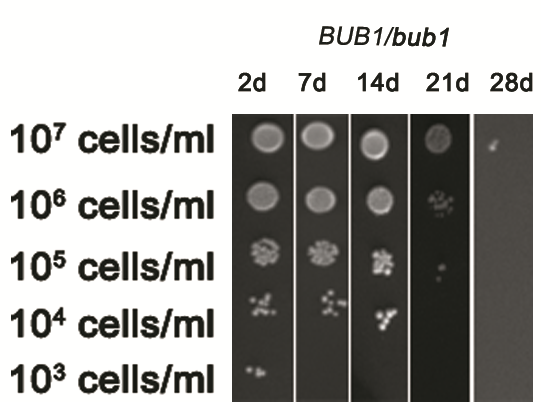 | 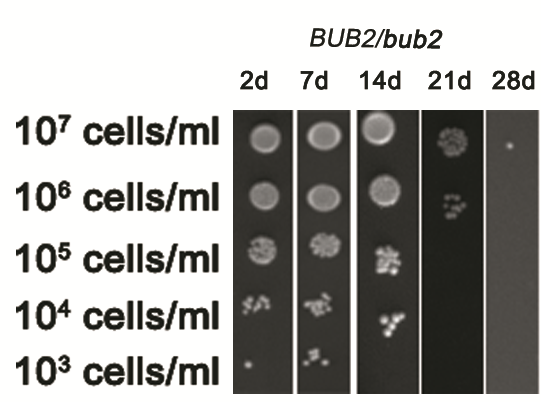 | 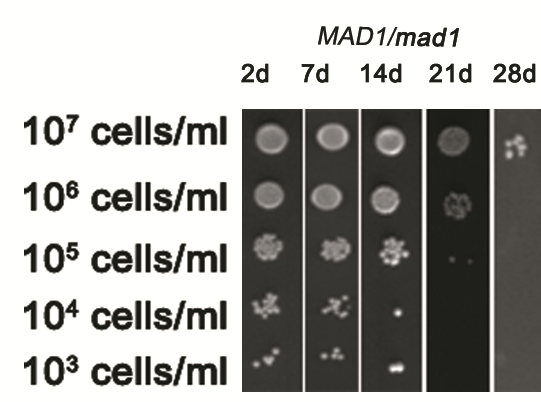 | 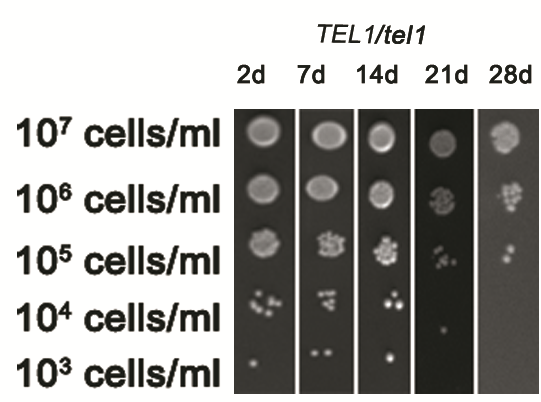 |

Supplement: Supplementary file 2 — Survival of haploid wild type strain BY4741 and isogenic bub1, bub2, mad1 and tel1 mutants (top panel), and diploid wild type strain BY4743 and isogenic BUB1/bub1, BUB2/bub2, MAD1/mad1 and TEL1/tel1 mutants (bottom panel) during CA (spot assay). After 2, 7, 14, 21 and 28 days, appropriate aliquots from CA cultures were taken for analysis. Several dilutions (107, 106, 105, 104, 103 cells/ml) of a yeast CA culture in a volume of 2 μl were used, inoculated on solid YPD medium and inspected after 48 h. The results shown are representative for at least three independent experiments (DOC 1381 kb) [file 10522_2014_9499_MOESM2_ESM.doc]

Supplemental Figure 3.

| ***mad1*** | ***MAD1/mad1*** |
| --- | --- |
| ***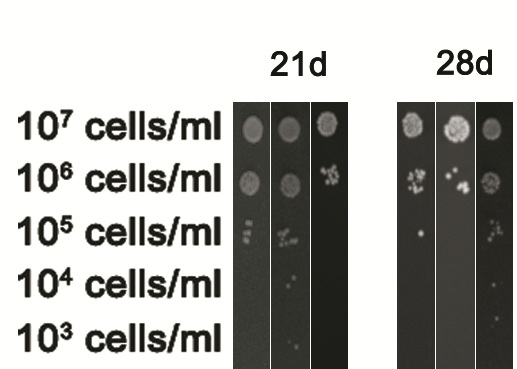*** | 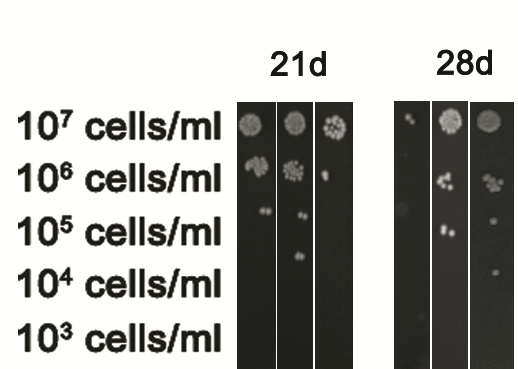 |
|  |  |
| ***tel1*** | ***TEL1/tel1*** |
| ***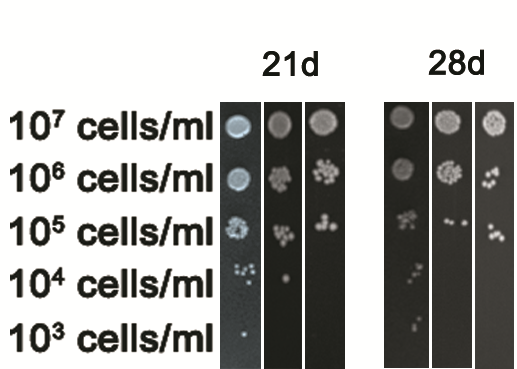*** | ***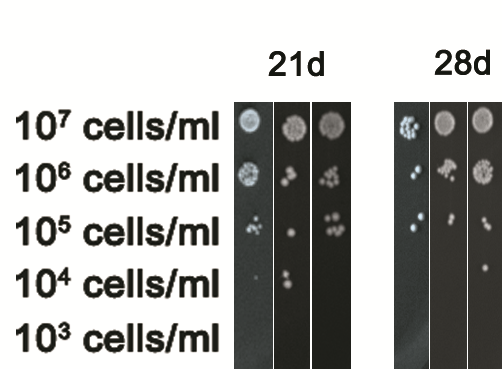*** |

Supplement: Supplementary file 3 — Late in the CLS experiment, a gasping phenomenon was revealed. After 21 and 28 days, appropriate aliquots from CA cultures were taken for analysis. Several dilutions (107, 106, 105, 104, 103 cells/ml) of a yeast CA culture in a volume of 2 μl were used, inoculated on solid YPD medium and inspected after 48 h. Three different growth patterns of selected time points are presented (DOC 457 kb) [file 10522_2014_9499_MOESM3_ESM.doc]

**Supplemental Figure 4.**

**A**

| 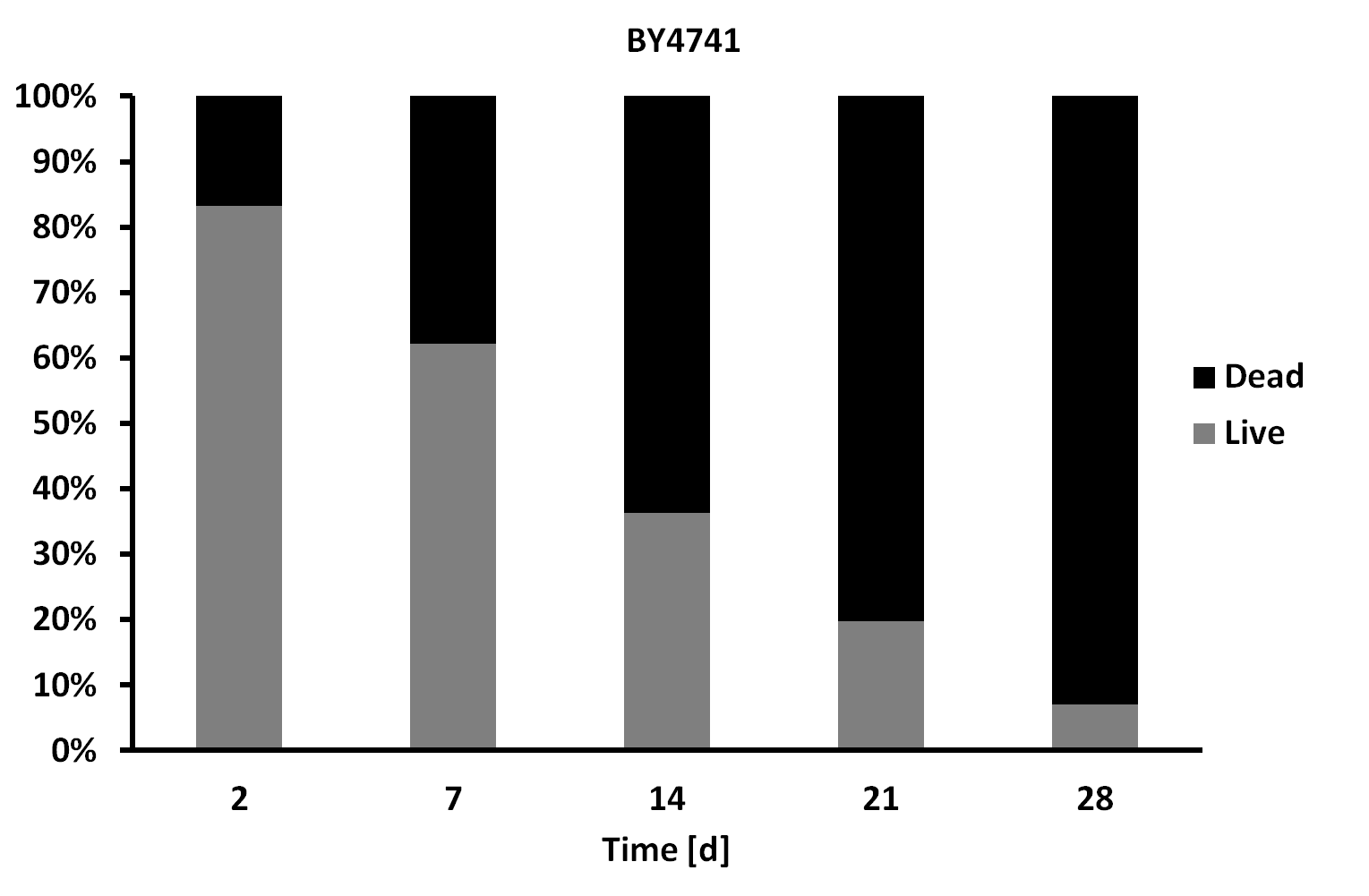 | 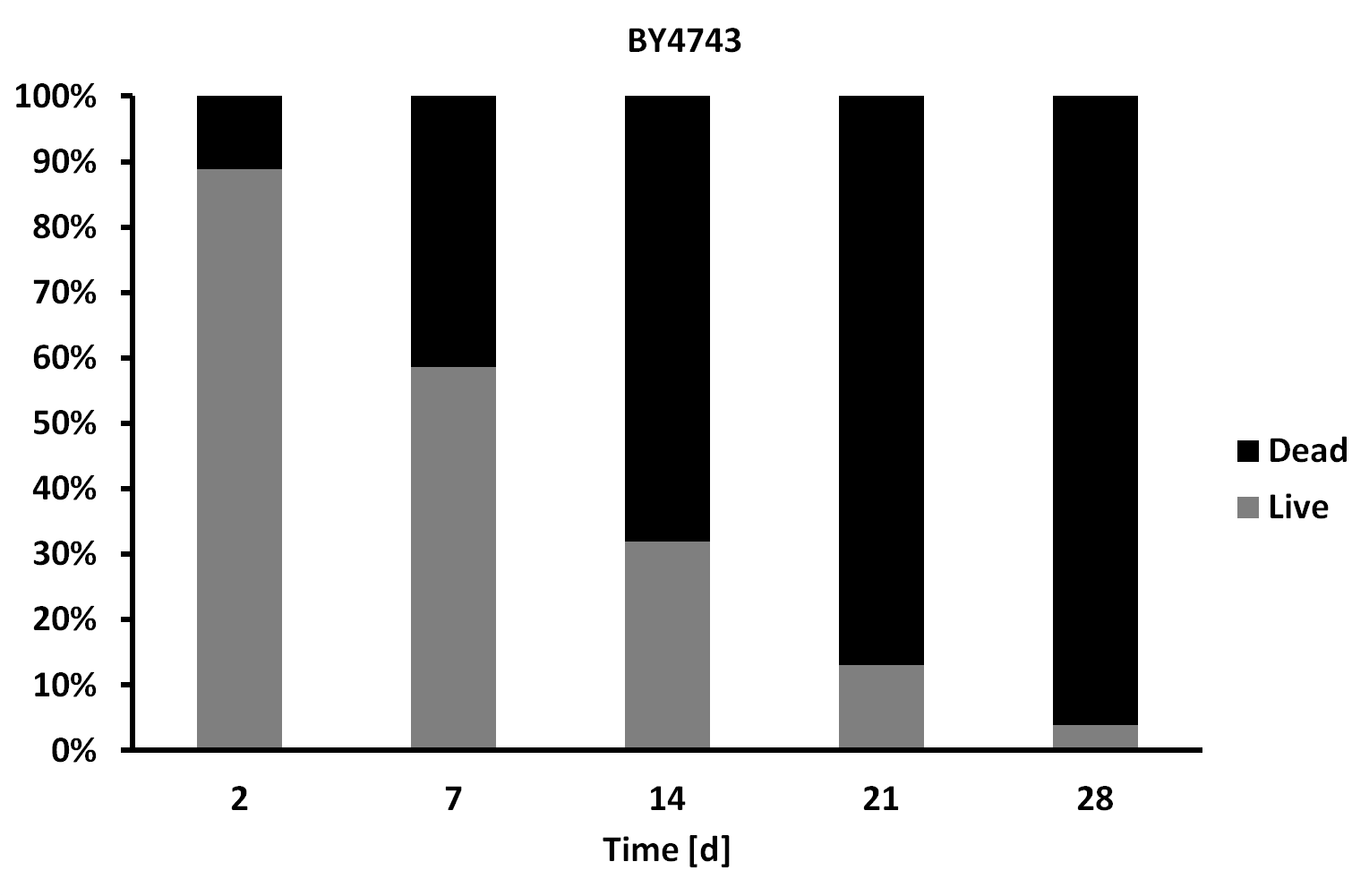 |
| --- | --- |
| 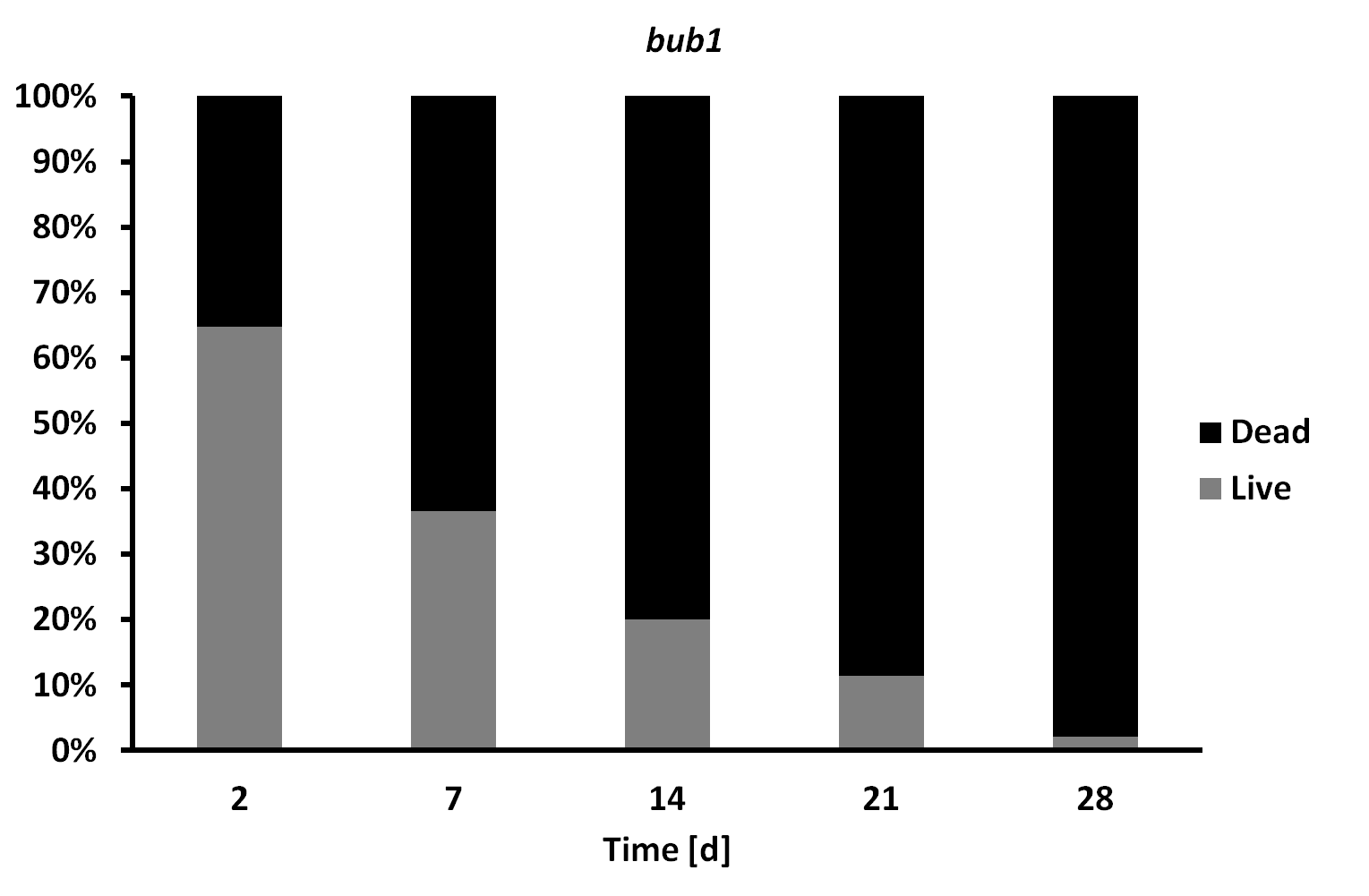 | 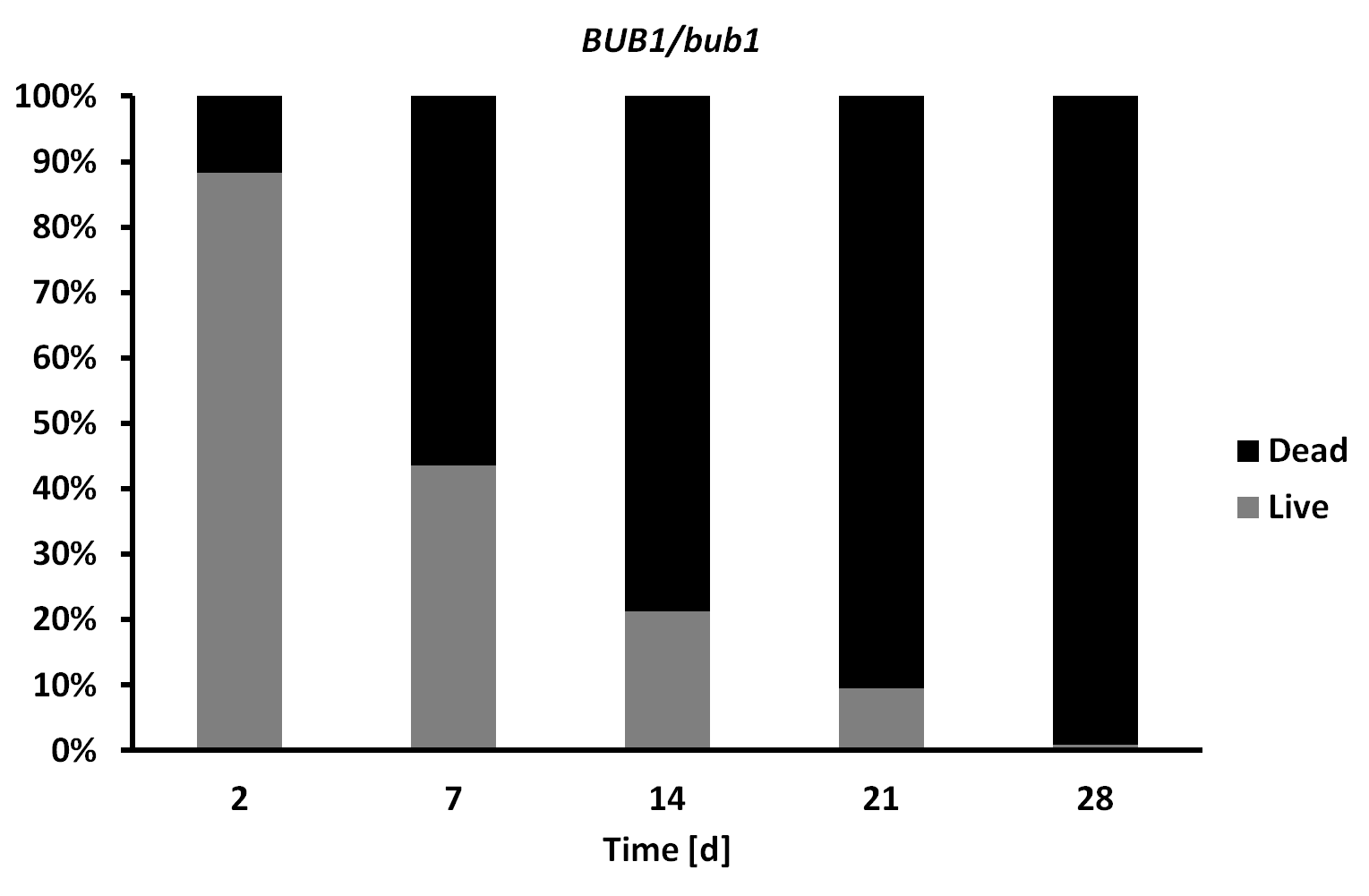 |
| 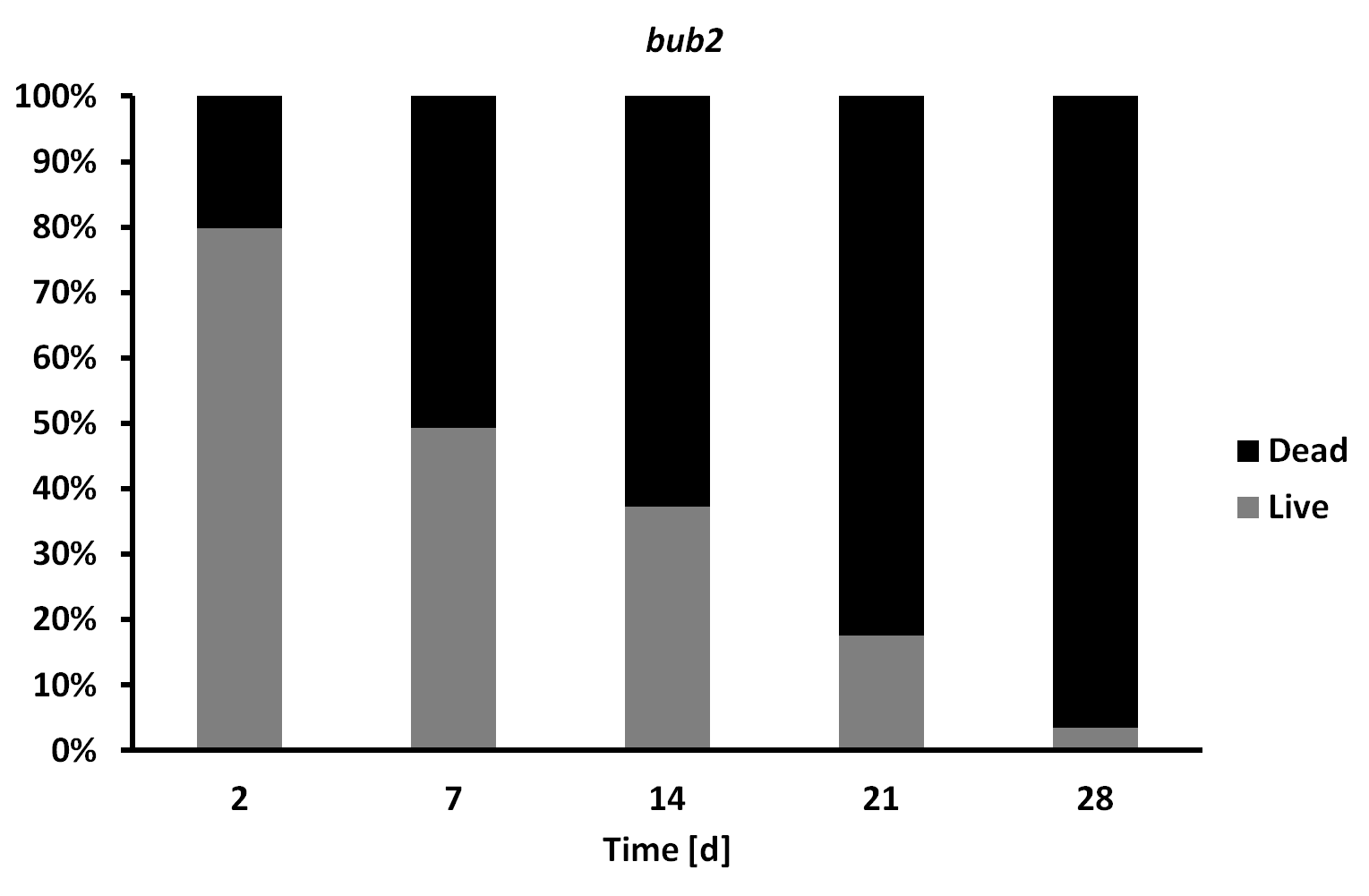 | 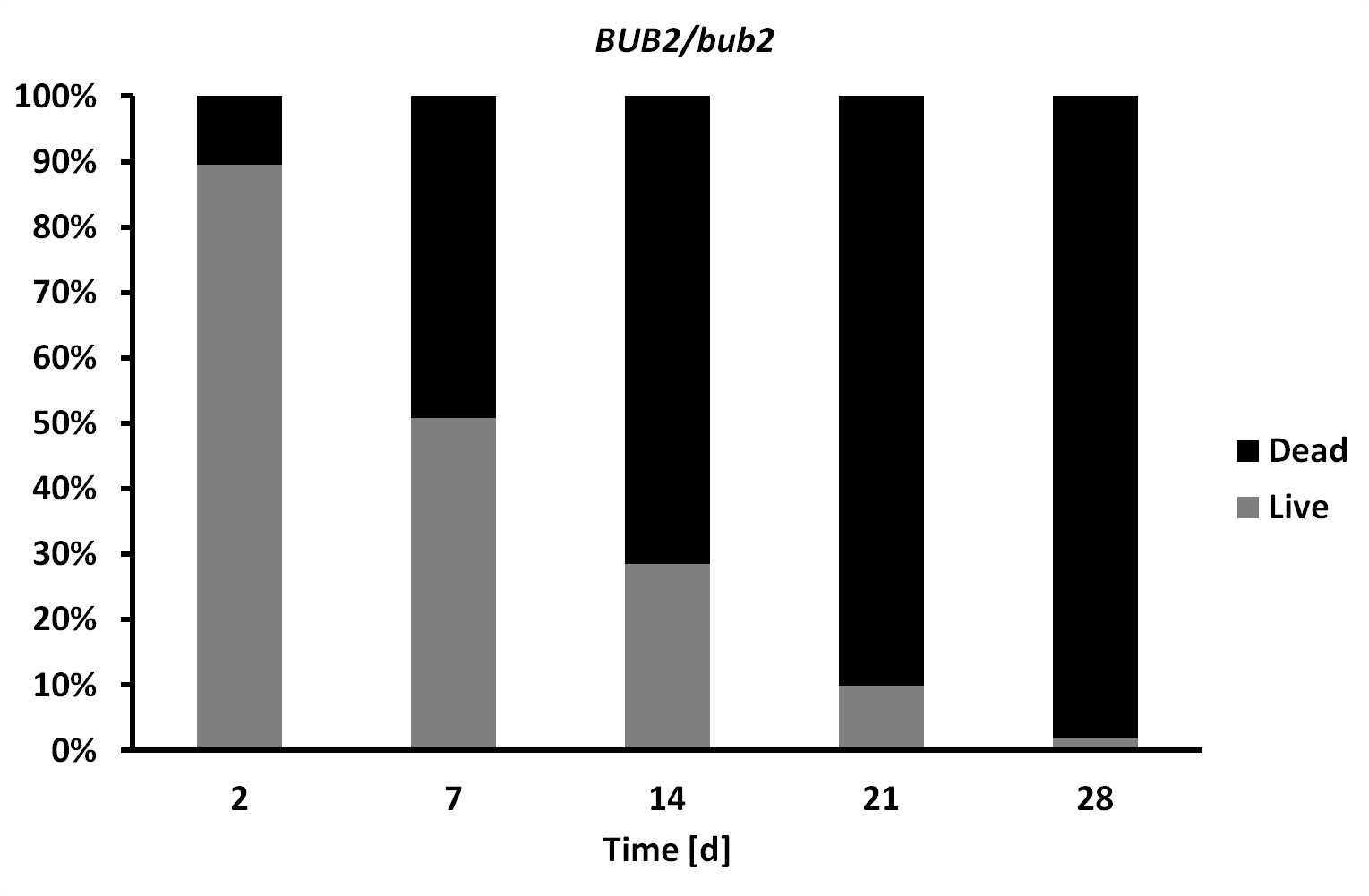 |
| 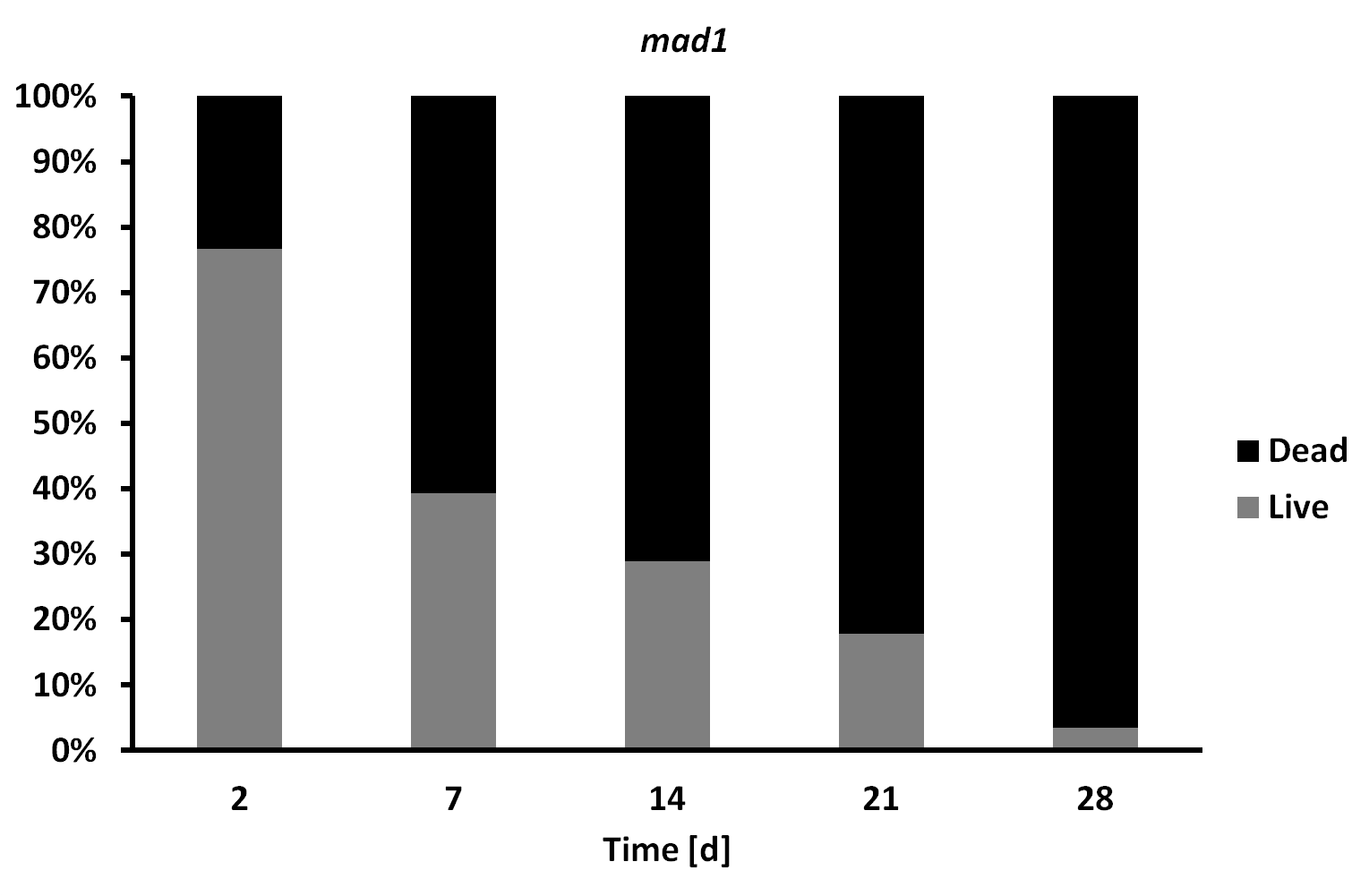 | 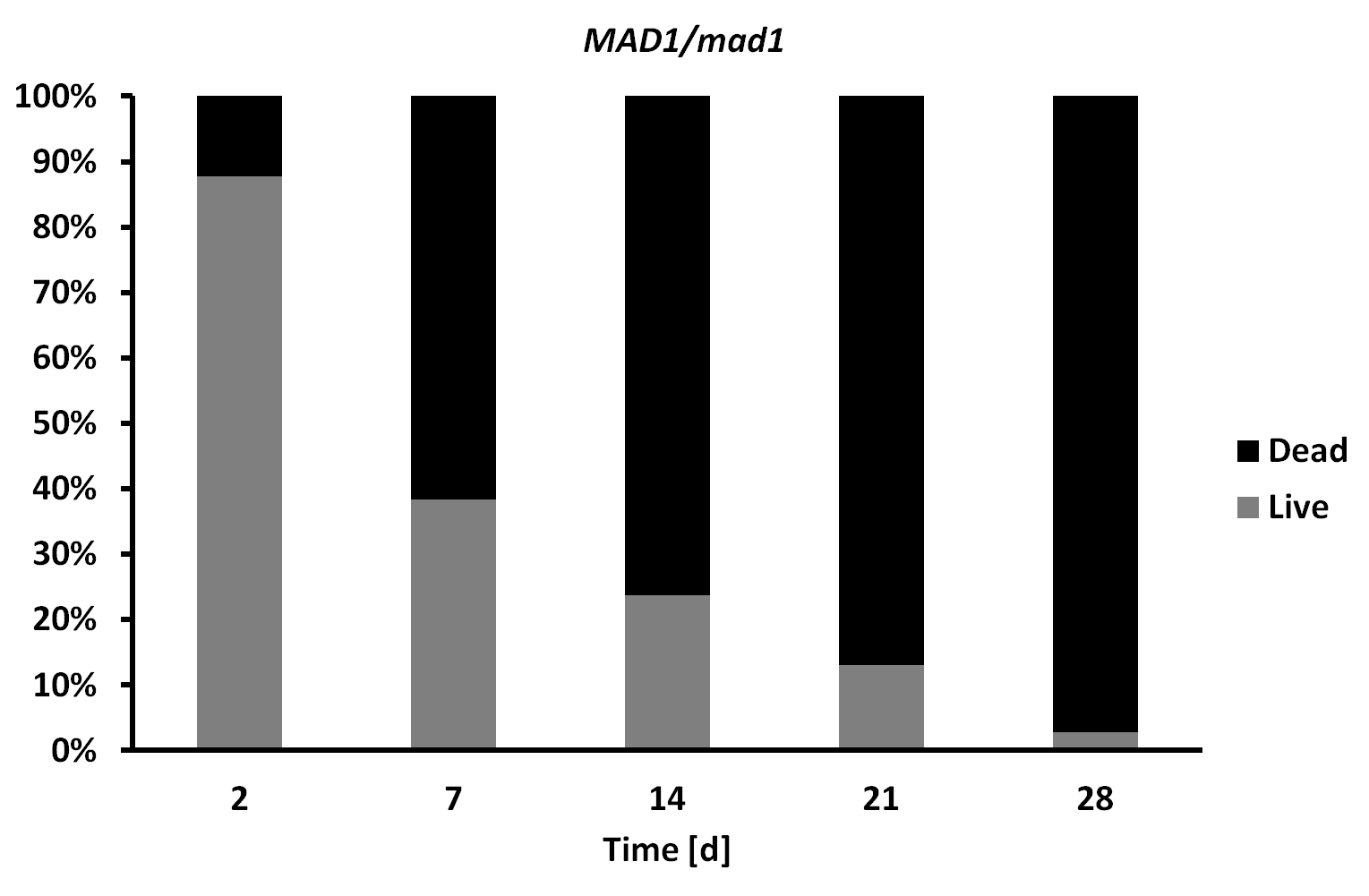 |
| 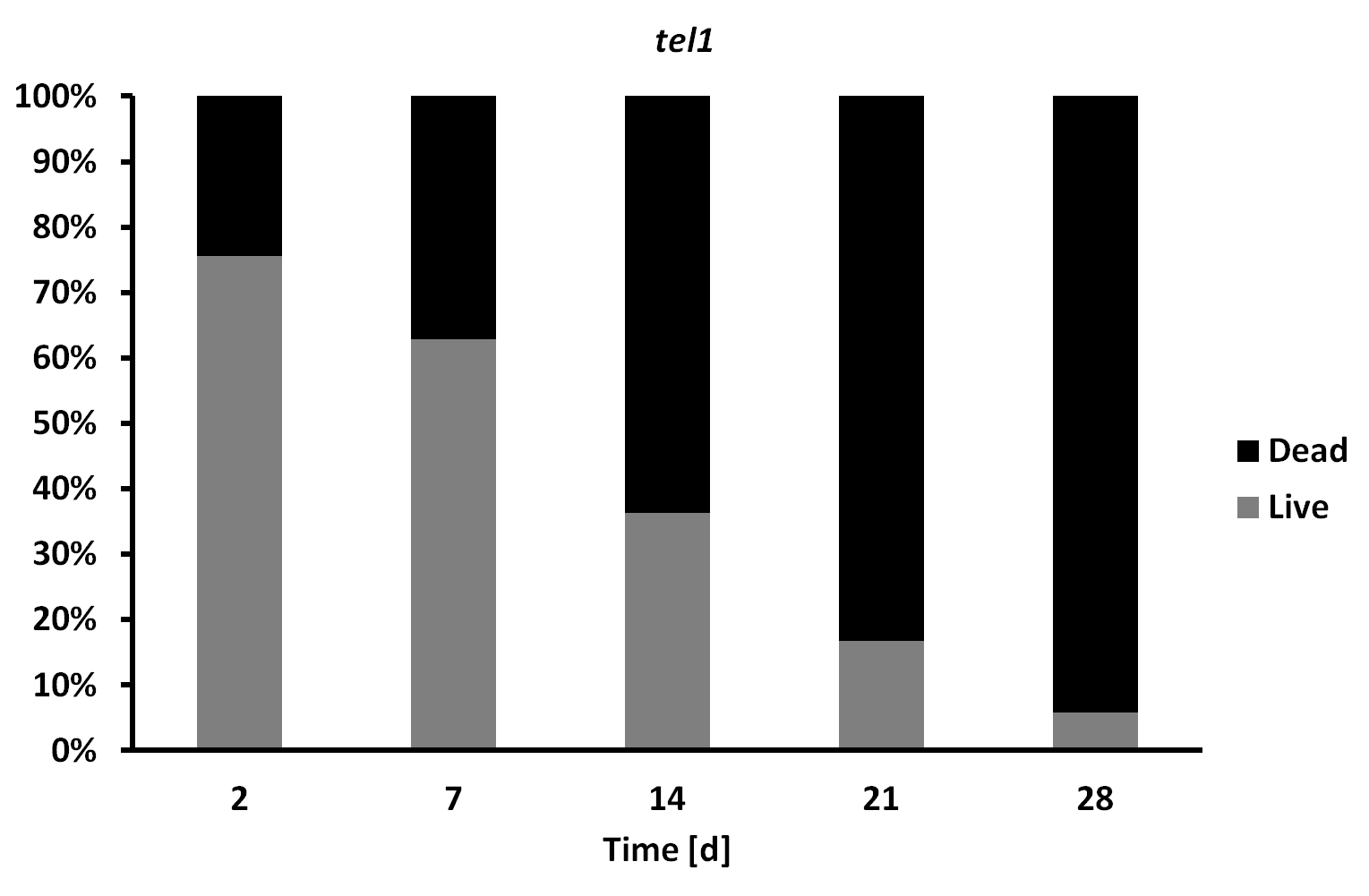 | 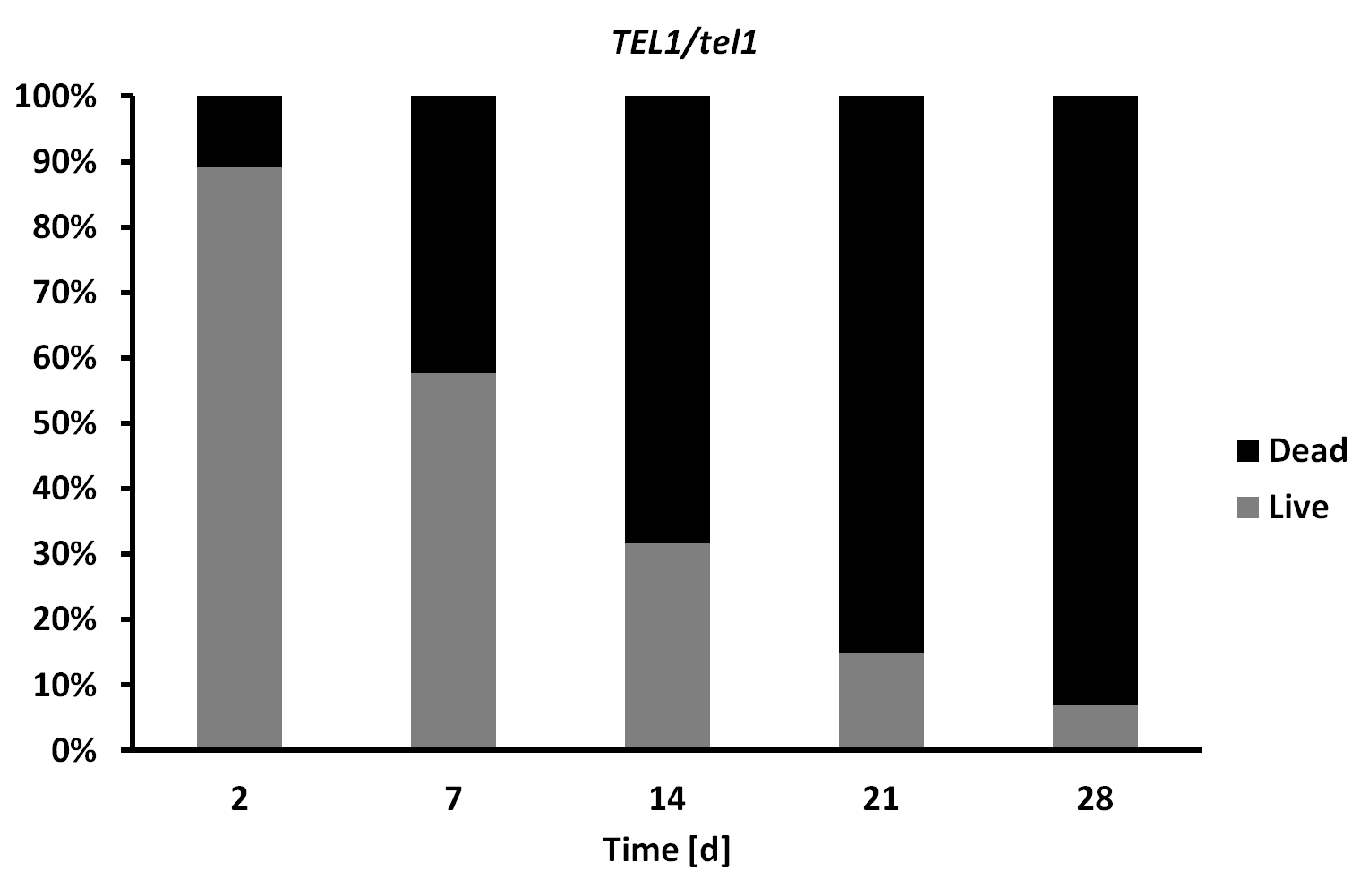 |

**Supplemental Figure 4 continued.**

**B**

**
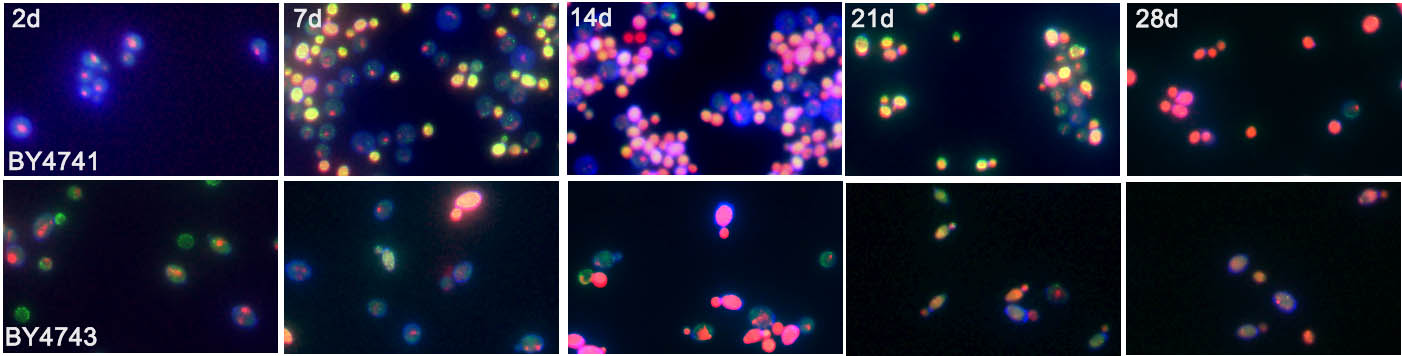
**

Supplement: Supplementary file 4 — CA-mediated cell viability (A) of haploid wild type strain BY4741 and isogenic bub1, bub2, mad1 and tel1 mutants (left panel), and diploid wild type strain BY4743 and isogenic BUB1/bub1, BUB2/bub2, MAD1/mad1 and TEL1/tel1 mutants (right panel). Cell viability was estimated with a LIVE/DEAD® Yeast Viability Kit (Molecular Probes) using the standard protocol according to the manufacturer’s instructions. Percentage of live and dead cells is shown. Typically, 300 cells were used for the analysis. The results shown are representative for at least three independent experiments. B) Representative micrographs are shown: haploid wild type BY4741 (top panel), diploid wild type BY4743 (bottom panel) (DOC 880 kb) [file 10522_2014_9499_MOESM4_ESM.doc]

Supplemental Figure 5.

| **A** | 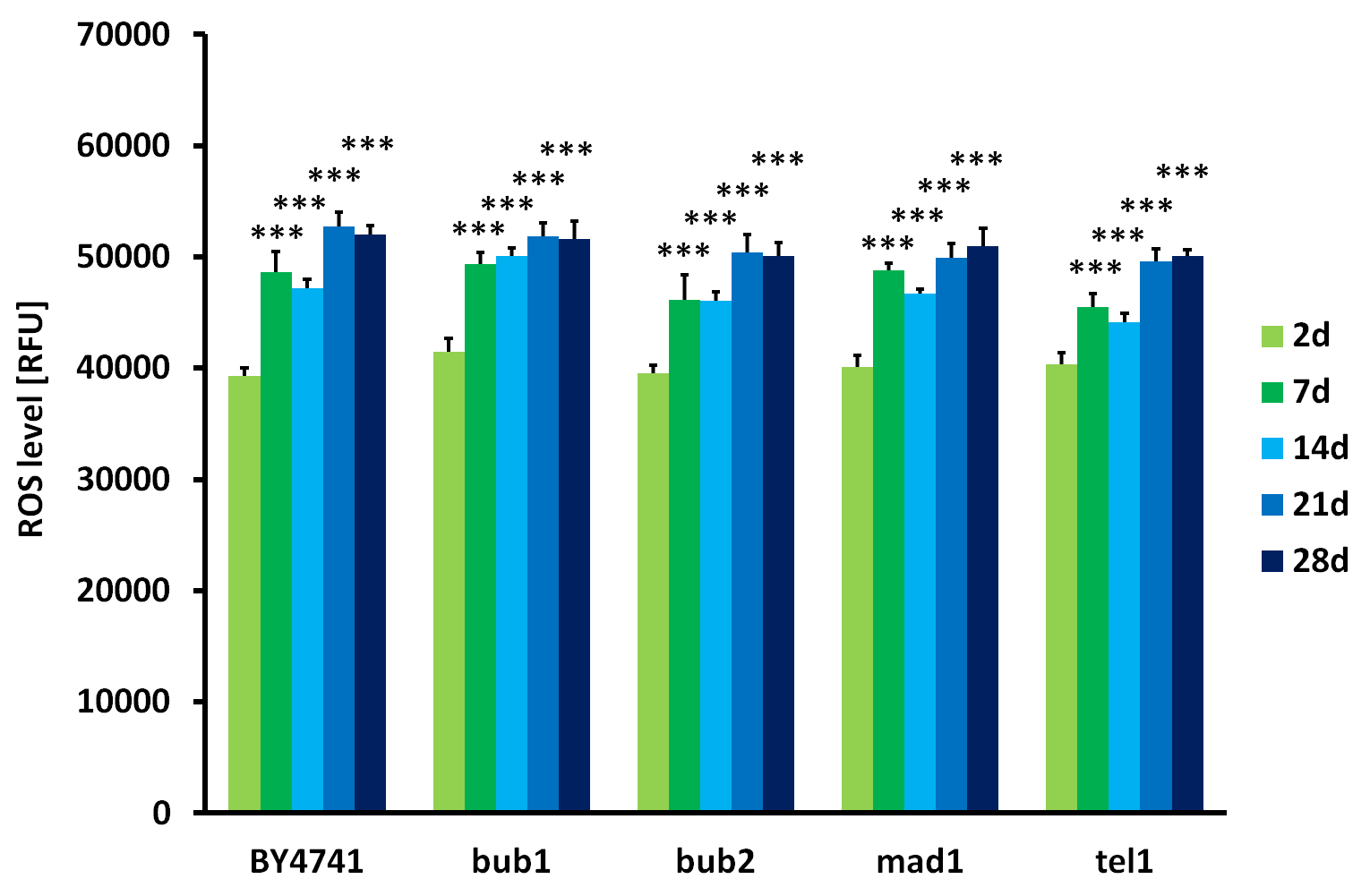 | 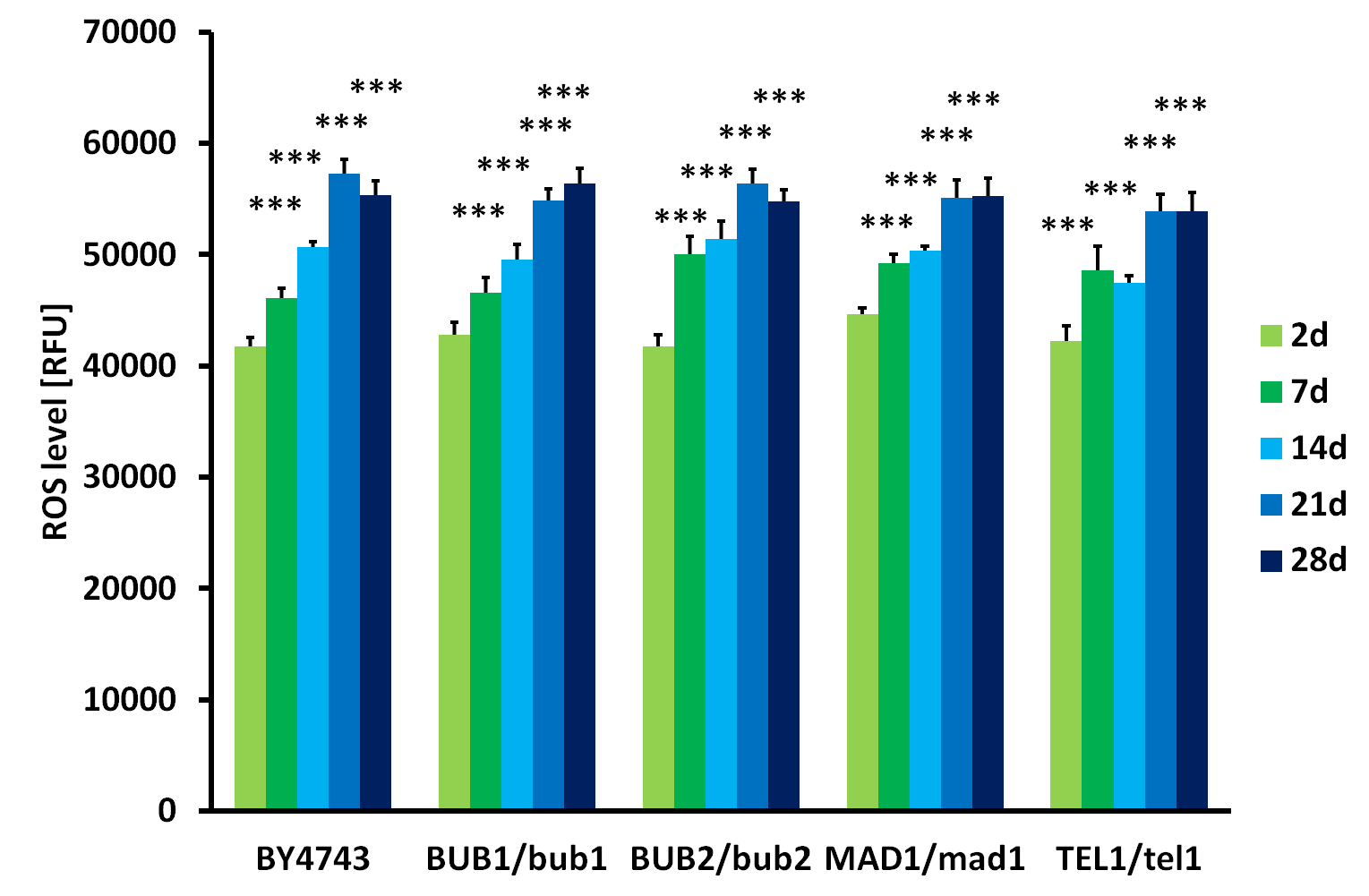 |
| --- | --- | --- |
| **B** | 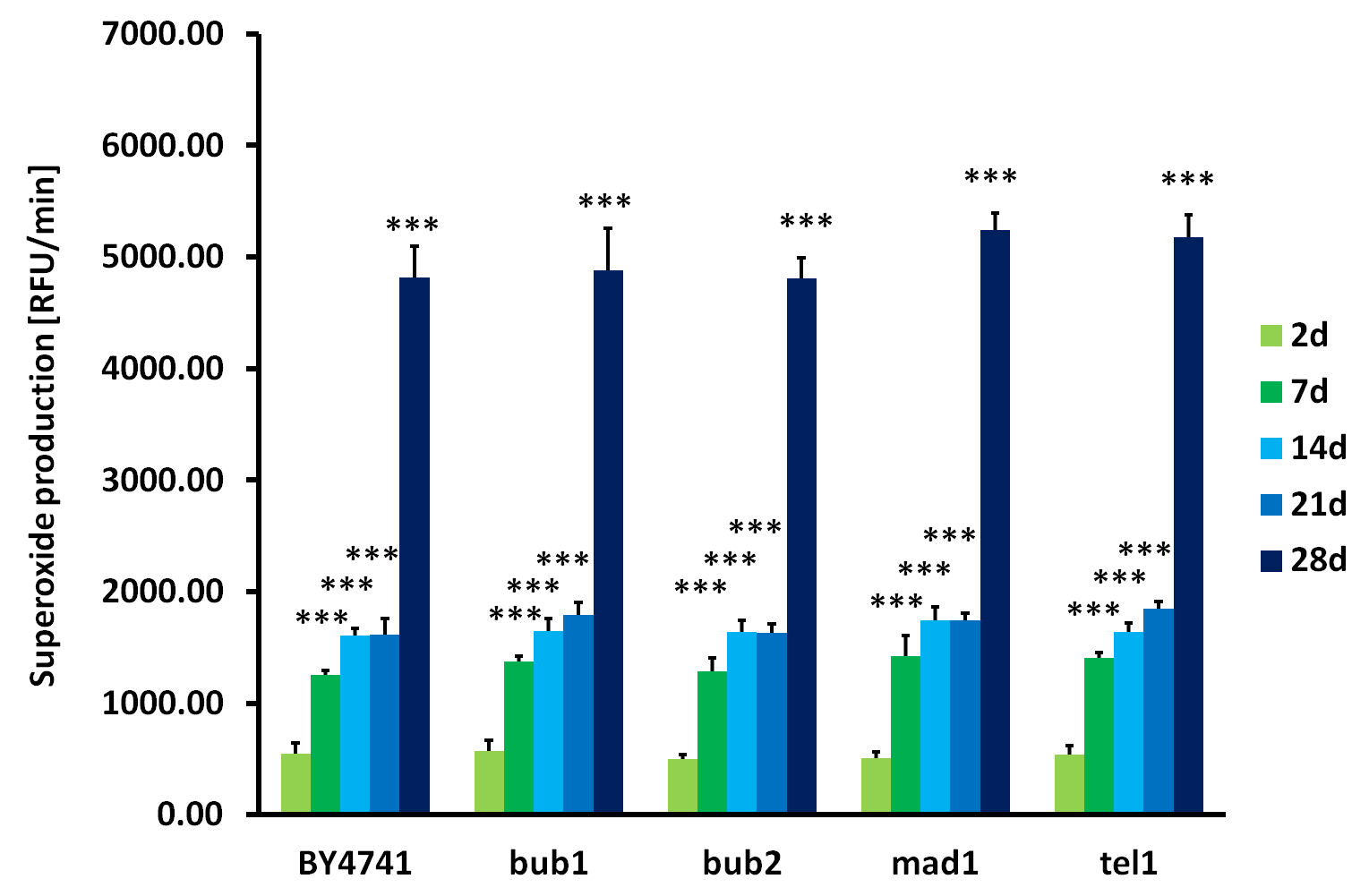 | 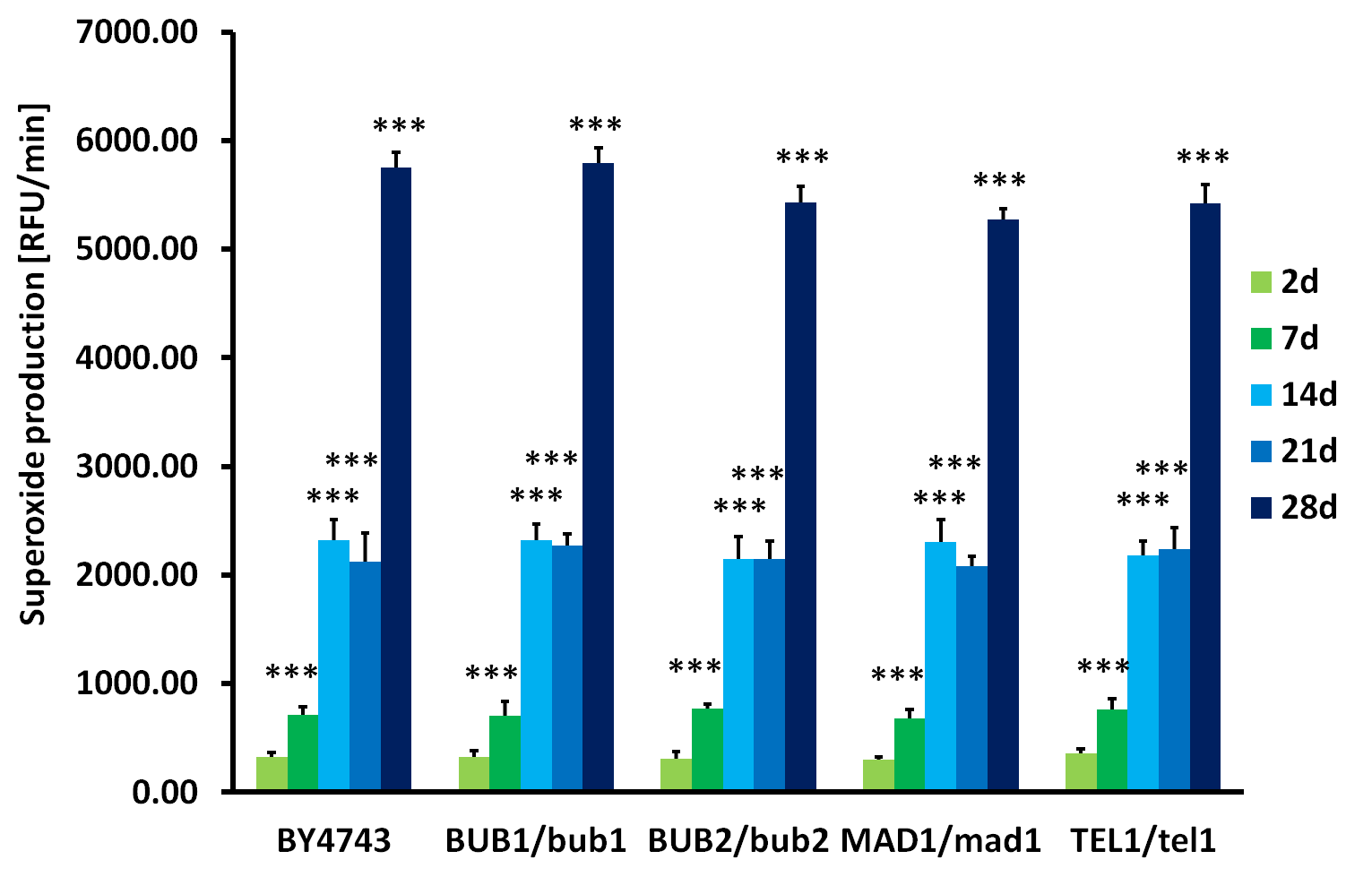 |
| **C** | 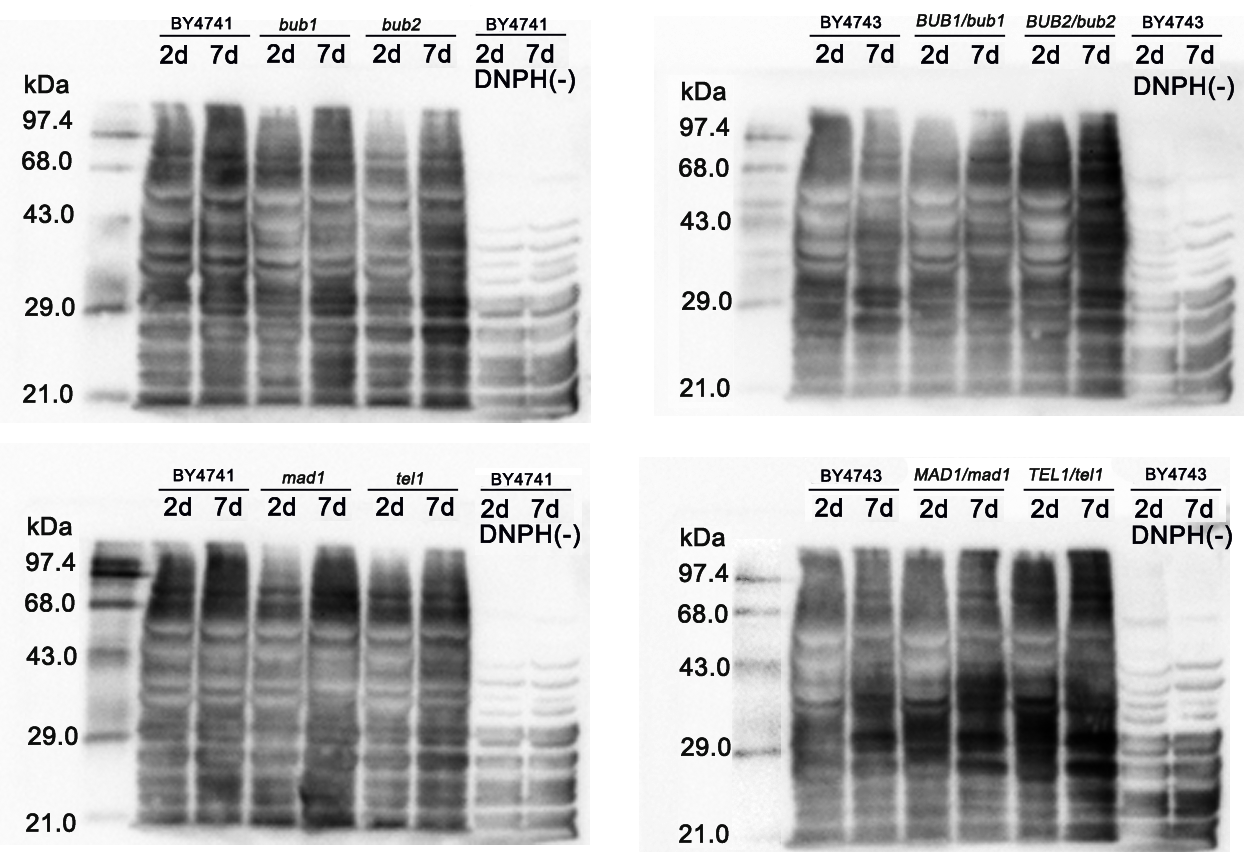 | |

Supplement: Supplementary file 5 — Chronological aging is accompanied by oxidative stress. A) Reactive oxygen species (ROS) level is increased in the culture medium during CA (media obtained from haploid strains - left panel, media obtained from diploid strains - right panel). After 2, 7, 14, 21 and 28 days, ROS level was measured with 2′,7′-dichlorodihydrofluorescein diacetate (H2DCF-DA). B) Intracellular superoxide production is augmented during CA (haploid strains - left panel, diploid strains - right panel). After 2, 7, 14, 21 and 28 days, superoxide kinetics was measured with dihydroethidium. Fluorescence intensity was monitored in a Tecan Infinite® M200 fluorescence mode microplate reader. A, B) Bars indicate SD, n = 3, *** p < 0.001 compared to day 2 of culture (control conditions) of a particular strain (ANOVA and Dunnett’s a posteriori test). C) Protein carbonylation is elevated during CA (haploid strains - left panel, diploid strains - right panel). After 2 and 7 days, protein carbonylation was revealed with 2,4-dinitrophenylhydrazine (DNPH) derivatisation and anti-DNP antibody (OxyBlot™ Protein Oxidation Detection Kit, Millipore). For every oxyblot, a negative control without DNPH derivatisation is shown (DOC 1112 kb) [file 10522_2014_9499_MOESM5_ESM.doc]

**Supplemental** Figure 6.

| **A** | **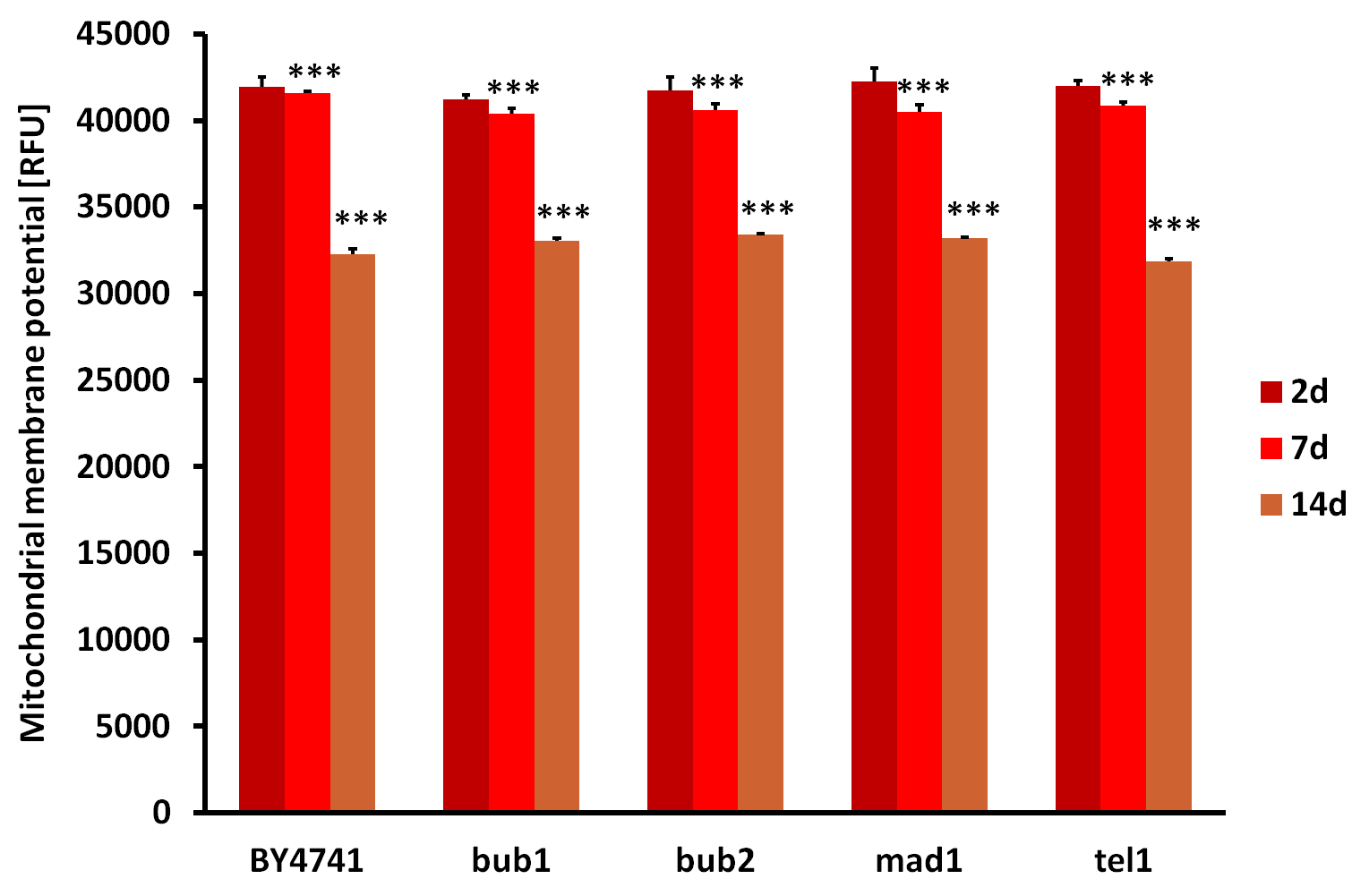** | **C**  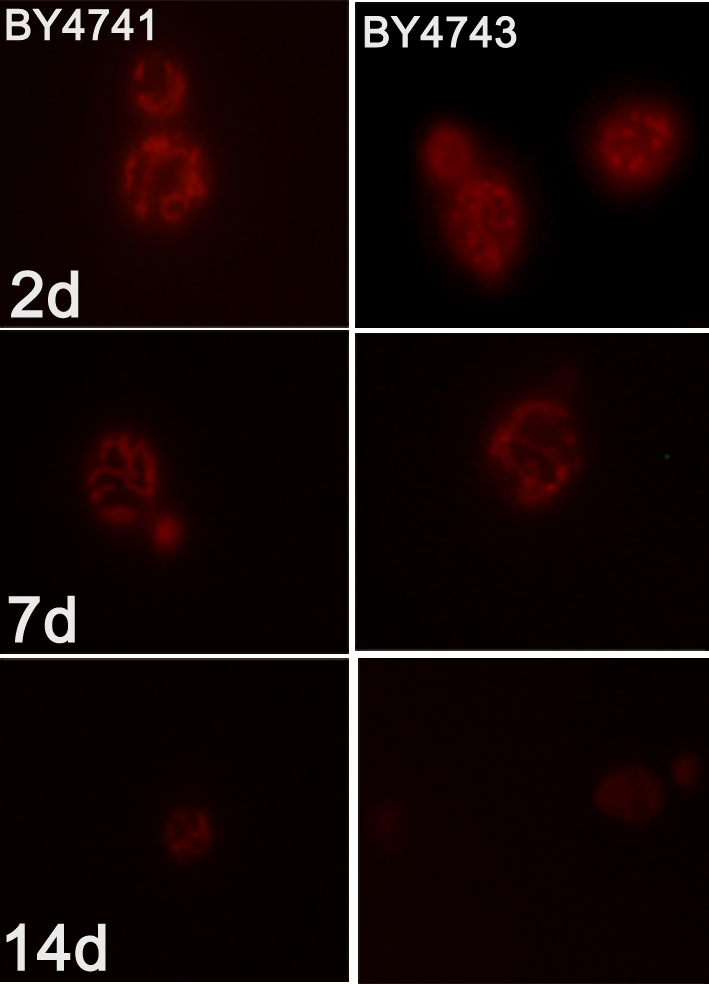 |
| --- | --- | --- |
| **B** | **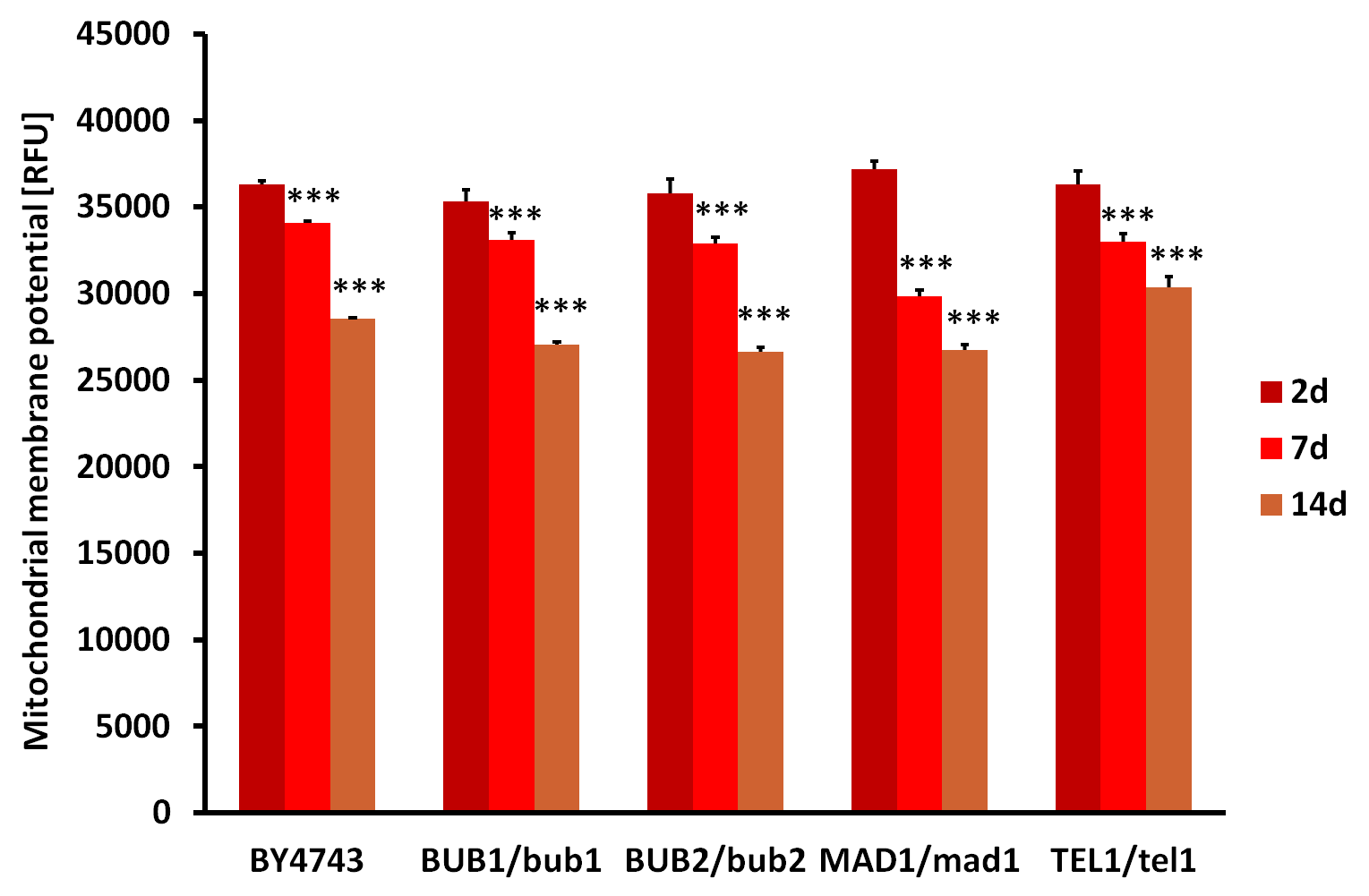** |

Supplement: Supplementary file 6 — CA-mediated changes in mitochondrial membrane potential (MMP). After 2, 7 and 14 days, the fluorescence intensity of rhodamine G6 reflecting the mitochondrial membrane potential was monitored in a Tecan Infinite® M200 fluorescence mode microplate reader. Mitochondrial membrane potential is presented as relative fluorescence units (RFUs). A) Haploid strains, B) diploid strains. Bars indicate SD, n = 3, *** p < 0.001 compared to day 2 of culture (control conditions) of a particular strain (ANOVA and Dunnett’s a posteriori test). C) Typical micrographs are shown. Cells per each sample triplicate were analysed using an Olympus BX61 fluorescence microscope equipped with a DP72 CCD camera and Olympus CellF software (DOC 493 kb) [file 10522_2014_9499_MOESM6_ESM.doc]

**Supplemental Figure 7.**


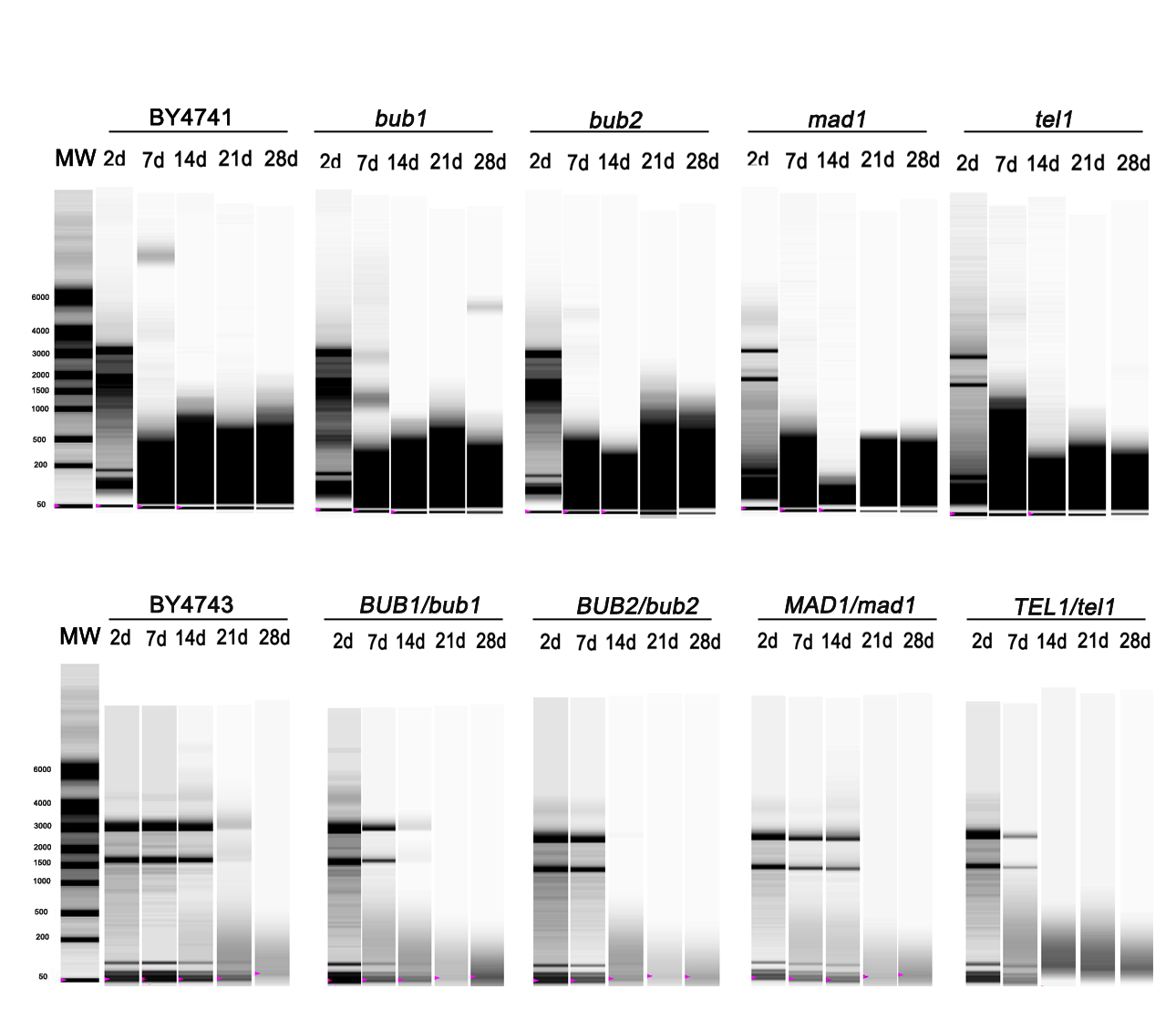

Supplement: Supplementary file 7 — CA-mediated RNA degradation. After 2, 7, 14, 21 and 28 days, RNA was isolated using an RNeasy Mini Kit (Qiagen) and RNA chip electrophoresis was performed with an Experion™ Automated Electrophoresis System and an Experion™ RNA StdSens Analysis Kit (Biorad). RQI (an RNA quality indicator) algorithm was used to assess RNA integrity by comparing the electropherogram of RNA samples to a series of standardised degraded RNA samples. RNA electropherograms were transformed to virtual gel images. Top panel: haploid strains, bottom panel: diploid strains. RNA molecular marker is also shown (Biorad) (DOC 215 kb) [file 10522_2014_9499_MOESM7_ESM.doc]

Supplemental Figure 8.

| **A** | 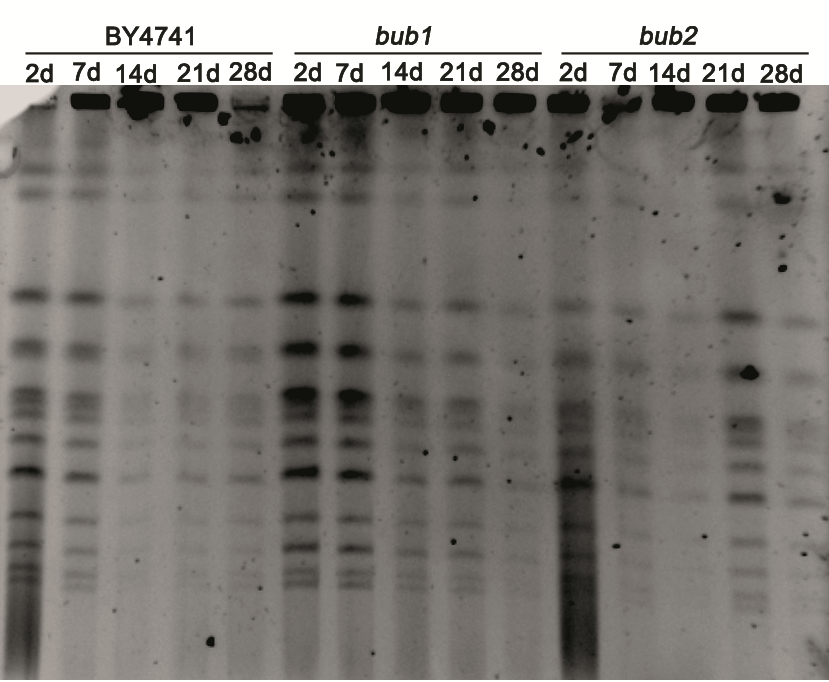 | 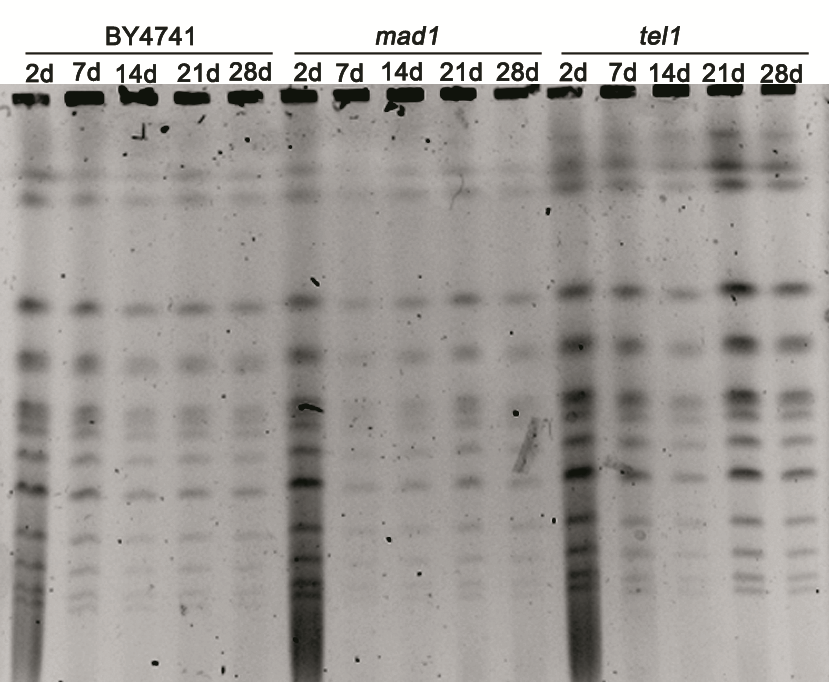 |
| --- | --- | --- |
|  |  |  |
| **B** | 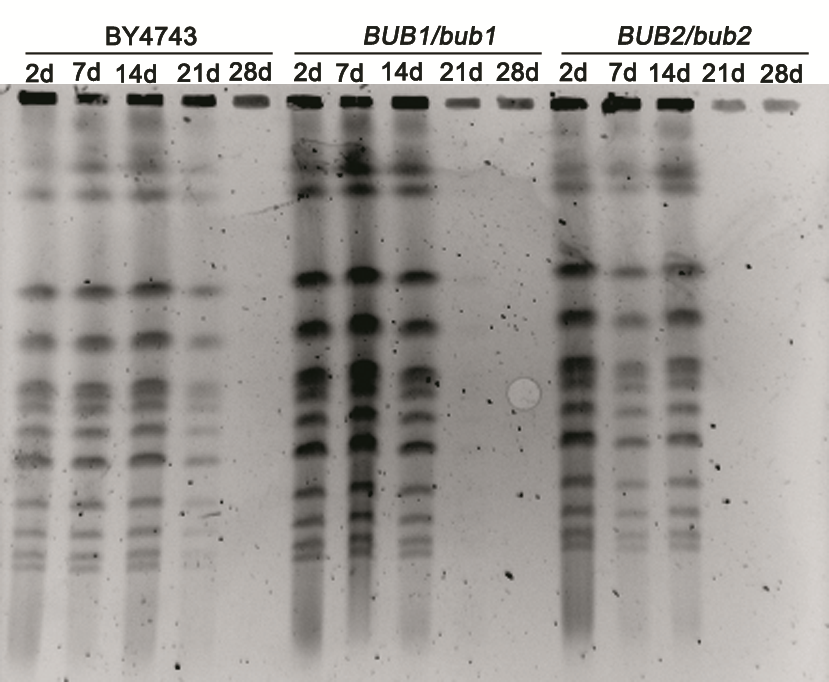 | 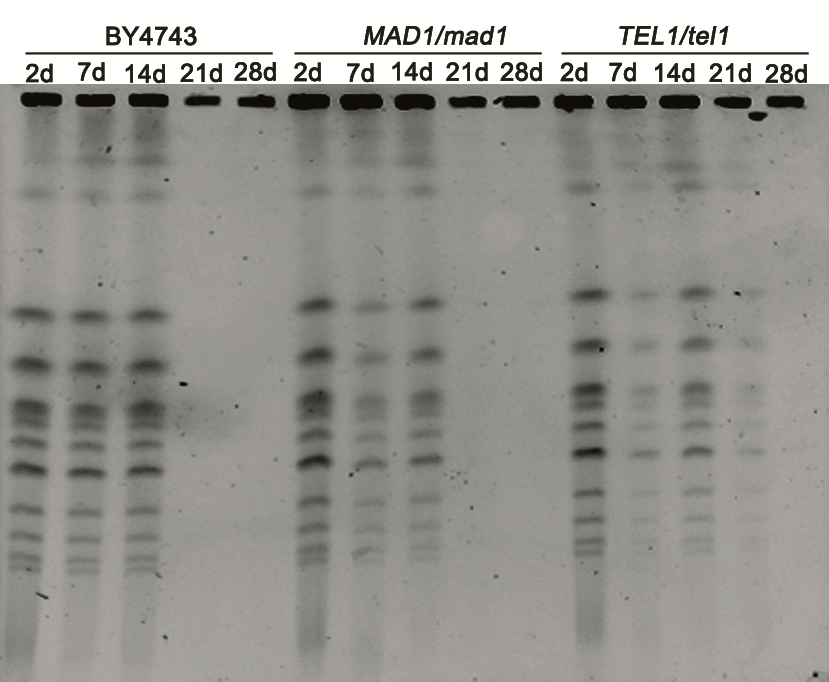 |

Supplement: Supplementary file 8 — CA-associated structural aberrations. After 2, 7, 14, 21 and 28 days, yeast chromosomes were separated with PFGE according to the manufacturer’s instructions using a CHEF-DR®III Pulsed Field Electrophoresis System (Biorad). A) Haploid strains, B) diploid strains. Typical micrographs are shown (DOC 2127 kb) [file 10522_2014_9499_MOESM8_ESM.doc]
